# *Pichia sorbitophila*, an Interspecies Yeast Hybrid, Reveals Early Steps of Genome Resolution After Polyploidization

**DOI:** 10.1534/g3.111.000745

**Published:** 2012-02-01

**Authors:** Véronique Leh Louis, Laurence Despons, Anne Friedrich, Tiphaine Martin, Pascal Durrens, Serge Casarégola, Cécile Neuvéglise, Cécile Fairhead, Christian Marck, José A. Cruz, Marie-Laure Straub, Valérie Kugler, Christine Sacerdot, Zlatyo Uzunov, Agnes Thierry, Stéphanie Weiss, Claudine Bleykasten, Jacky De Montigny, Noemie Jacques, Paul Jung, Marc Lemaire, Sandrine Mallet, Guillaume Morel, Guy-Franck Richard, Anasua Sarkar, Guilhem Savel, Joseph Schacherer, Marie-Line Seret, Emmanuel Talla, Gaelle Samson, Claire Jubin, Julie Poulain, Benoît Vacherie, Valérie Barbe, Eric Pelletier, David J. Sherman, Eric Westhof, Jean Weissenbach, Philippe V. Baret, Patrick Wincker, Claude Gaillardin, Bernard Dujon, Jean-Luc Souciet

**Affiliations:** * Université de Strasbourg, CNRS UMR7156, F-67000 Strasbourg, France; † Université de Bordeaux 1, LaBRI INRIA Bordeaux Sud-Ouest (MAGNOME), F-33405 Talence, France; ‡ INRA UMR 1319 Micalis, AgroParisTech, Bat. CBAI, F-78850 Thiverval-Grignon, France; § Institut de Génétique et Microbiologie, Université Paris-Sud, UMR CNRS 8621, F-91405 Orsay CEDEX, France; ** Institut de Biologie et de Technologies de Saclay (iBiTec-S), CEA, F-91191 Gif-sur-Yvette CEDEX, France; †† Université de Strasbourg, Architecture et Réactivité de l’ARN, Institut de Biologie Moléculaire et Cellulaire du CNRS, F-67084 Strasbourg, France; ‡‡ Institut Pasteur, CNRS URA2171, Université Pierre et Maris Curie, Paris 6 UFR927, F-75724, Paris-CEDEX 15, France; §§ Sofia University St. Kliment Ohridski, Faculty of Biology, Department of General and Applied Microbiology, 1164, Sofia, Bulgaria; *** Université de Lyon, F-69622, Lyon, France; Université Lyon 1, Villeurbanne; CNRS, UMR5240 Microbiologie, Adaptation et Pathogénie; INSA de Lyon, F-69621, Villeurbanne, France; ††† Université de Bordeaux 1, CNRS UMR5800, F-33405 Talence, France; ‡‡‡ Earth and Life Institute, Université Catholique de Louvain, B-1348, Louvain-la-Neuve, Belgium; §§§ Université de la Méditerranée, Laboratoire de Chimie Bactérienne, CNRS-UPR9043, 31 chemin Joseph Aiguier, F-13402 Marseille CEDEX 20, France; **** CEA, DSV, IG, Génoscope; CNRS UMR 8030; Université d’Evry Val d’ Essonne, 2 rue Gaston Crémieux, F-91057 Evry, France

**Keywords:** osmotolerant yeast *P. sorbitophila*, allopolyploidy, hybridization, genome evolution, loss of heterozygosity

## Abstract

Polyploidization is an important process in the evolution of eukaryotic genomes, but ensuing molecular mechanisms remain to be clarified. Autopolyploidization or whole-genome duplication events frequently are resolved in resulting lineages by the loss of single genes from most duplicated pairs, causing transient gene dosage imbalance and accelerating speciation through meiotic infertility. Allopolyploidization or formation of interspecies hybrids raises the problem of genetic incompatibility (Bateson-Dobzhansky-Muller effect) and may be resolved by the accumulation of mutational changes in resulting lineages. In this article, we show that an osmotolerant yeast species, *Pichia sorbitophila*, recently isolated in a concentrated sorbitol solution in industry, illustrates this last situation. Its genome is a mosaic of homologous and homeologous chromosomes, or parts thereof, that corresponds to a recently formed hybrid in the process of evolution. The respective parental contributions to this genome were characterized using existing variations in GC content. The genomic changes that occurred during the short period since hybrid formation were identified (*e.g.*, loss of heterozygosity, unilateral loss of rDNA, reciprocal exchange) and distinguished from those undergone by the two parental genomes after separation from their common ancestor (*i.e.*, NUMT (NUclear sequences of MiTochondrial origin) insertions, gene acquisitions, gene location movements, reciprocal translocation). We found that the physiological characteristics of this new yeast species are determined by specific but unequal contributions of its two parents, one of which could be identified as very closely related to an extant *Pichia farinosa* strain.

A new species could arise via a process of interspecific hybridization, that is, the union of different organisms across a species barrier. At the early stage, hybridization produces allodiploid or allopolyploid hybrids, depending on the ploidy of parents. This situation gives rise to a period of genome resolution where diverse genetic events act simultaneously and successively to form a stable chimerical genome. Recombination, loss of chromosomes and loss of heterozygosity (LOH) are frequent mechanisms involved in this genome shuffling that stabilizes hybrids (Belloch *et al.* 2009; Forche *et al.* 2005; Gerstein *et al.* 2006; Scannell *et al.* 2006; Sipiczki 2008). Because of the combination and modification of at least two distinct gene pools, hybrids could display better adaptive properties than their parental species, called heterosis (Arnold and Martin 2010; Belloch *et al.* 2008). The limitation on the amount of such interspecies hybrids is often related to genetic incompatibilities, leading to fitness and survival decrease and/or fertility reduction (Johnson 2008). Hybridization occurs approximately in 25% of plant species and 10% of animal species (Mallet 2005).

Recent genomic data and experimental analyses suggest that interspecies hybrids are frequent in yeast populations. Several interspecific hybrids have been described among species that belong to the genus *Saccharomyces* and play key roles in industrial fermentations (Bond *et al.* 2009; Dunn and Sherlock 2008; Nakao *et al.* 2009; Querol and Bond 2009; Rainieri *et al.* 2006; Sipiczki 2008). In contrast, only few cases of interspecific hybrids have been reported among other yeasts or fungi: the fungal pathogens *Candida albicans* and *Candida dubliniensis* can mate to produce tetraploid hybrids (Pujol *et al.* 2004), a random genomic sequencing of a *Zygosaccharomyces rouxii* wild isolate showed that it contains two different sets of genes (Gordon and Wolfe 2008), and finally, among *Basidiomycota*, anomalous *Cryptococcus neoformans* strains isolated from patients appeared to be hybrids between *C. neoformans* and *C. gattii* (Bovers *et al.* 2006). If stress conditions in cultures have been invoked to stimulate the formation of yeast hybrids (Belloch *et al.* 2008; Querol and Bond 2009), experiments show that yeast species in general tend to have no or limited prezygotic barriers (Chou *et al.* 2010; Greig *et al.* 2002; Liti *et al.* 2006; Marinoni and Lachance 2004; Murphy *et al.* 2006). In addition, only rare cases of heterosis or Bateson-Dobzhansky-Muller incompatibility are reported in yeasts (Belloch *et al.* 2008; Chou *et al.* 2010; Kao *et al.* 2010; Lee *et al.* 2008). Therefore, the role of interspecies crosses in yeast populations need to be better clarified, as well as the events involved in the hybrid genome formation and stabilization.

The yeast *P. sorbitophila* (de Miranda *et al.* 1980), a member of the “CTG” group of *Saccharomycotina* (De Montigny *et al.* 2000; Kurtzman and Suzuki 2010), has been largely studied for its resistance to osmotic and salt stress (Banuelos *et al.* 2002; Benito *et al.* 2004; Lages *et al.* 1999; Lages and Lucas 1995; Maresova and Sychrova 2003; Neves *et al.* 2004; Oliveira *et al.* 1996; Prista *et al.* 2005). This osmotolerant yeast was isolated as a contaminant of a 70% sorbitol solution. *P. sorbitophila* resists to very high NaCl concentration (4M NaCl), whereas *Debaryomyces hansenii* and *Pichia farinosa*, two closely related species, are inhibited. de Miranda *et al.* (1980) and Oliveira *et al.* (1996) reported that *P. sorbitophila* is able to form asci with ascospores. The ability of crossing clones from ascospores to each other led to propose that *P. sorbitophila* may be homothallic (Oliveira *et al.* 1996).

By the complete sequencing and the detailed analysis of its genome, we demonstrate here that *P. sorbitophila* is, in fact, a hybrid yeast. From its genome, we extracted the two subgenomes, inherited from both parents. We identified genes for specific metabolism pathways, acquired either from both parents or from a sole parent, giving now the opportunity to study possible cases of heterosis. We finally demonstrate that *P. sorbitophila* is a very recent hybrid in the process of its resolution. The genomic rearrangements characterizing the early steps of genome stabilization were distinguished from those appeared in the parental genomes before the hybridization event. The hybrid state of the *P. sorbitophila* genome raises now the question about the fertility of this yeast.

## Methods

**Figure 1:**
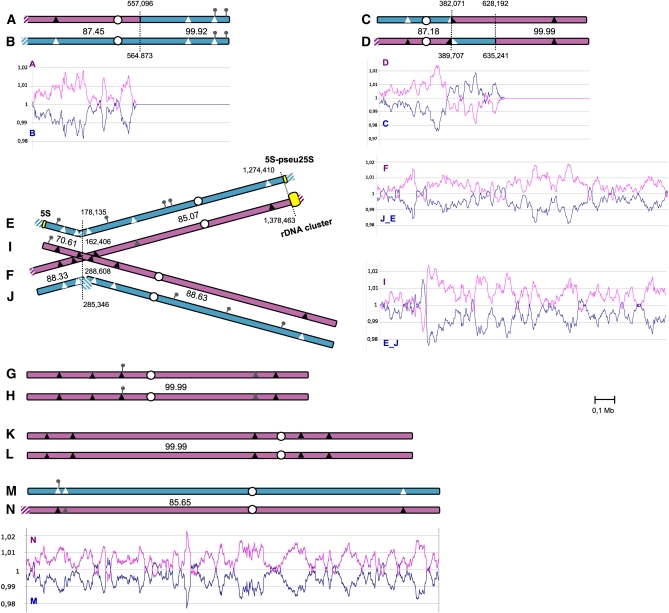
The hybrid nuclear genome of *P. sorbitophila*. The 14 chromosomes are represented by pairs according to their synteny. Chromosomes A/B and C/D are partly heterozygous, partly homozygous. Chromosomes E/F/I/J and M/N are heterozygous, and G/H and K/L are homozygous. The translocation breakpoint observed between chromosomes E, F, I, and J is represented by crossed chromosomes. Red and blue colors correspond to the proposed Pγ and Pε parental subgenomes, respectively, and hatched boxes to synteny losses between the homeologous chromosomes. Percentages of nucleotide identity between heterozygous regions are also indicated. Position of predicted centromeres (Figure S4) and of the 15 clusters of NUMT loci comprising a total of 24 NUMTs (Table S3) are shown by white dots and pins, respectively. Triangles indicate the positions of selected sequences used in *P. farinosa* CBS2001 strain (Table S4). The nucleotide identity level observed between *P. sorbitophila* and *P. farinosa* for the selected sequences is represented as followed: black triangles for 100% identity, gray for 99% and white for less than 96%. Below each chromosome pair (or at right for the crossed chromosomes) is represented the GC content variation (as shown in *Materials and Methods* and Figure S5) calculated for genes present at two allelic copies in chromosome pairs. The colors for curves correspond to subgenomes as for chromosomes.

Full methods are available in the online version with the supporting information. Here are described the methods used for the genome sequencing and assembly, and the determination of the global GC content variations between Pγ and Pε subgenomes, depicted in Figure 1.

### Sequencing and assembly

The sequencing of the nuclear genome of *Pichia sorbitophila* CBS 7064 (de Miranda *et al.* 1980) was performed using a whole-genome shotgun strategy, with two plasmid libraries (4-kb inserts and 40-kb inserts) and ABI/Sanger technology to 10X-15X depth. In a first read assembly performed using ARACHNE assembler_version 03 (Batzoglou *et al.* 2002), we obtained 17 supercontigs, from 0.144 Mb to 2.114 Mb in size, with a read coverage of 7.5X on average for 11 contigs and 14X for 6 contigs (supporting information, Figure S1). The weakly covered contigs were joined into five distinct pairs showing approximately 85% sequence identity between contigs forming a pair, reflecting the partial heterozygosity of the genome (Figure S3). The homozygous genomic regions were represented by the six contigs 14X covered. Some of the weakly and highly covered contigs could also be joined on the basis of the sequence identity shared at the end of the contigs, corresponding to partly heterozygous and partly homozygous chromosomes (Figure S1). We confirmed this assembly by reiterating all the process using ARACHNE assembler_version 04 and with a manual finishing step to join contigs and resolve low-quality sequences. Homozygous sequences were doubled, and some of them concatenated with heterozygous sequences if necessary, to be representative of the genome state. The presence of small heterozygous regions inside homozygous areas, potentially masked during the assembly process, was checked by analyzing single-nucleotide polymorphism (SNP) distribution (see Table S2 for method). No area with a complex pattern of heterozygosity/homozygosity was highlighted inside the homozygous regions. At the end of that process, 14 contigs were obtained and named in alphabetical order according to their size. Only two sequence gaps remained in chromosomes G and H telomeric repeats. This assembly was validated by comparing with the number and the size of chromosomes estimated by pulsed-field gel electrophoresis (Figure S2), one contig corresponding to one chromosome.

### GC content calculation

The GC content along chromosomes was computed using all protein coding genes present in two syntenic copies between chromosomes forming a pair and having identical sizes to minimize the effect of insertions/deletions on the GC values (2499 gene pairs considered). The two GC% obtained for a gene pair were compared with each other by calculating their ratio (dGC) from the mean GC% value: dGC_1_ = GC_1_ / ((GC_1_ + GC_2_)/2) and dGC_2_ = GC_2_/((GC_1_ + GC_2_)/2), with GC_1_ and GC_2_ corresponding to the GC% values obtained for copies 1 and 2, respectively. This normalization does not allow us to take into account the GC content variation between genes. Therefore, when both copies of a gene are identical at the nucleotide level (a situation observed in homozygous regions), the following identity relation is attributed to these two alleles: dGC_1_ = dGC_2_ = 1. Conversely, for two nonidentical copies, the gene copy that contains the highest GC content has its dGC > 1. The two dGC values obtained for each gene pair were plotted along chromosome pairs using a sliding window of 11 genes with a step of 1 for representing a moving average of 11 adjacent genes along each chromosome. Curve superposition was performed using a single chromosomal coordinate. This coordinate corresponds to the start codon of one gene located on an arbitrarily chosen chromosome of each pair. The GC content variations in all protein coding genes were also calculated using tRNA that pair with two codons (Crick 1966). The codon usage was determined for each heterozygous region, independently from its parental origin, using genes that are present in two syntenic copies (5516 gene pairs considered). According to the GC trend curves, data obtained for all heterozygous regions belonging to the same subgenome were pooled to determine the average variation observed between both subgenomes.

## Results and Discussion

### Structure of the *Pichia sorbitophila* hybrid genome

#### Size of the genome, number of chromosome pairs, and global heterozygosity:

The nuclear genome of *Pichia sorbitophila* CBS 7064 (also referenced as *Pichia* or *Millerozyma farinosa*) (Kurtzman and Suzuki 2010) was completely sequenced using a whole-genome shotgun strategy and initially assembled into 17 supercontigs (see *Materials and Methods* and Figure S1). Sequence alignments revealed nearly perfect synteny conservation and approximately 85% identity at nucleotide level between some pairs of supercontigs (11 supercontigs), suggesting a partial heterozygosity of the genome. The other supercontigs (6 supercontigs) remained unique. Interestingly, sequencing coverage was double for the latter compared with the former (Figure S1), suggesting that they represent homozygous regions with two identical or almost identical copies of sequence and that the read assembly led to the production of a single consensus sequence for each homozygous part. A manually finishing process confirmed that the *P. sorbitophila* genome is a mosaic of homologous and homeologous chromosome pairs (see *Materials and Methods*). We obtained 14 contigs (named chr. A to N), from 1.05 Mb to 2.12 Mb, representing the 14 chromosomes of the *P. sorbitophila* nuclear genome and giving a global genome size of 21.5 Mb (Table S1). Contig sizes largely match chromosome sizes estimated by pulsed-field gel electrophoresis (Figure S2). Telomeric repeats were found at the ends of almost all contigs (Table S1). As suspected, the 14 chromosomes could be gathered into seven pairs of homologous or homeologous chromosomes (Figure 1) on the basis of the sequence identity along the chromosomes (Figure S3). The chromosome pairs A/B and C/D were found to be partly heterozygous and partly homozygous, G/H and K/L homozygous, and M/N and E/F/I/J heterozygous. These last four chromosomes could not be separated into two clearly distinct pairs since a switch of identity was observed from E/I to E/F and from F/J to I/J (Figure 1 and Figure S3), suggesting that a translocation event has occurred between two of these chromosomes.

The global nucleotide sequence identity between heterozygous regions range from 70.6 to 88.6% (see Table S1). Some local loss of identity (hemizygous regions) are detected at the end of some chromosomes and in dispersed intrachromosomal areas (see Figure S3). If we do not consider these hemizygous regions, the remaining syntenic parts of the genome (11.85 Mb) shows no more than 89.16% nucleotide identity, revealing that *P. sorbitophila* is an hybrid genome derived from two distinct progenitors that have a high level of nucleotide polymorphism (10.84% of divergence) but a very well conserved synteny.

#### Homozygous regions and junctions between homozygous and heterozygous regions:

Forty percent of the genome is homozygous (two identical or almost identical copies of sequence) and result from LOH events (Figure 1 and Table S1). LOHs concern chromosome pairs either in their totality (chr. G/H and K/L) or in part (chr. A/B and C/D). We quantified the number of SNPs in homozygous regions by realigning initial reads against the consensus sequences given by the supercontigs (see Table S2 for data and method). As a whole, the polymorphism is very low (0.46 SNP per 10 kb) and reveals a LOH process recently started. According to the chromosome pairs considered, different polymorphism levels are observed, from 1 SNP per 51,474 bp within C/D to 1 SNP per 18,000 bp within G/H. The first LOH event involved probably the G/H pair, the most polymorphic region, and may have been followed by the additional events in successive order: K/L, A/B, and C/D.

We also examined the junctions between the heterozygous and homozygous regions in the A/B and C/D chromosome pairs. In both cases, they are located inside protein coding genes and the two allelic copies of these genes are highly conserved at the nucleotide level (95.7% for A/B and 99.3% for C/D). It contrasts with the average identity observed for the others heterozygous genes (90.5%) and for the surrounding heterozygous regions (91%). No repetitive element or low complexity sequence is present at these junctions.

#### Position of centromeres:

In *Debarymoyces hansenii* (Dujon *et al.* 2004), the most closely related yeast to *P. sorbitophila* (Figure 3), each chromosome has a unique island of highly repeated and degenerated sequences of retrotransposons Tdh5 with a poor GC content, which probably corresponds to the centromere (Butler *et al.* 2009; Dujon 2010; Lynch *et al.* 2010). In *P. sorbitophila*, we did not find any repetitive elements such as retrotransposons, but we identified for each chromosome a unique island with a poor GC content (10.8% less than the global GC content, see Figure S4), corresponding likely to the centromere position. These GC-poor regions range from 2.2 to 3.1 kb in size, are devoid of protein-coding genes or other features and are at equivalent positions in chromosomes forming a pair (Figure 1 and Figure S4). For homeologous pairs of chromosomes, all the proposed centromeres are located in heterozygous regions and share a very weak sequence identity or none, although surrounding allelic regions are well conserved (see Figure S3).

### Extraction of Pγ and Pε parental genomes

In the case of *P. sorbitophila* and contrary to the analyses conducted so far on other yeast hybrids (Bovers *et al.* 2006; Dunn and Sherlock 2008; Gordon and Wolfe 2008; Nakao *et al.* 2009; Rainieri *et al.* 2006; Sipiczki 2008), the extraction method of the two parental subgenomes cannot be based on knowledge of the genomic sequence of at least one species closely related to one parent. Therefore, we used two complementary strategies to extract the parental subgenomes. A first strategy, determined by the GC content, led us to propose a subdivision of the genome. The proposed assignation of genomic regions to each subgenome was then tested and completed by a comparative analysis performed with the species *Pichia farinosa* CBS 2001.

#### GC variation and bias in codon usage:

The global GC content calculated along chromosomes systematically revealed two different GC tendencies in heterozygous regions: each homeologous pair shows one chromosome with a greater GC content (average dGC = 1.006; σ = 0.012) and one chromosome with a lower GC content (average dGC = 0.994; σ = 0.012), as shown in Figure 1 using protein coding genes present in two syntenic copies between chromosomes forming a pair (Method described in the *Materials and Methods*). We hypothesized that it may reflect the subtle differences in nucleotide composition between the two parental genomes at the origin of the *P. sorbitophila* hybrid genome. We arbitrarily named “Pγ” the parental subgenome with the highest GC content and “Pε” the parental subgenome with the lowest one. We tested that hypothesis by analyzing the GC content variations in all protein coding genes (independently of their size) using tRNAs that pair with two different codons (Crick 1966). As shown in Table 1, genes in heterozygous regions defined as Pγ contain on average 1.57% more codons with a C at the third position than genes of Pε regions (mean = 1.56 [1.08-1.95]), allowing to propose an assignation of heterozygous regions to each subgenome (Figure 1, see also Figure S5). Applied to homozygous regions, the GC-based analyses suggested that both G/H and K/L pairs belong to Pγ subgenome (Figure S5). As for A/B and C/D homozygous parts, the discrimination was not obvious.

**Table 1:** Bias in codon usage between Pγ and Pε subgenomes

Amino Acid	Codon	No. Codons		Usage, %		Pγ-Pε
		Pγ	Pε	Pγ	Pε	
Phe	TTT	35,134	37,411	50.56	53.55	2.99
	TTC	34,354	32,448	49.44	46.45	
Val	GTT	29,941	30,325	59.98	61.56	1.58
	GTC	19,977	18,937	40.02	38.44	
Ser	TCT	34,498	36,153	64.45	66.26	1.80
	TCC	19,030	18,412	35.55	33.74	
Pro	CCT	24,501	25,360	66.37	67.88	1.51
	CCC	12,414	11,999	33.63	32.12	
Thr	ACT	25,044	26,245	58.24	60.78	2.55
	ACC	17,961	16,932	41.76	39.22	
Ala	GCT	30,768	31,479	61.14	63.54	2.4
	GCC	19,557	18,060	38.86	36.46	
His	CAT	20,343	20,787	58.51	60.07	1.56
	CAC	14,426	13,818	41.49	39.93	
Asn	AAT	48,995	51,063	53.57	55.28	1.71
	AAC	42,467	41,316	46.43	44.72	
Asp	GAT	55,556	56,476	57.36	58.56	1.21
	GAC	41,307	39,959	42.64	41.44	
Cys	TGT	10,196	10,161	55.71	55.19	0.52
	TGC	8105	8250	44.29	44.81	
Ser	AGT	21,219	21,365	50.48	50.81	0.33
	AGC	20,817	20,682	49.52	49.19	
Gly	GGT	30,801	29,869	64.52	63.82	0.70
	GGC	16,937	16,931	35.48	36.18	
Avg.						1.57
Mean						1.56
Q1-Q3						[1.08-1.95]

For tRNA species that pair with two codons, the usage % of each codon was determined as follows: (number of one codon/number of both codon ×100). The values were calculated for all chromosomal regions defined as belonging to Pγ or Pε.

**Figure 2:**
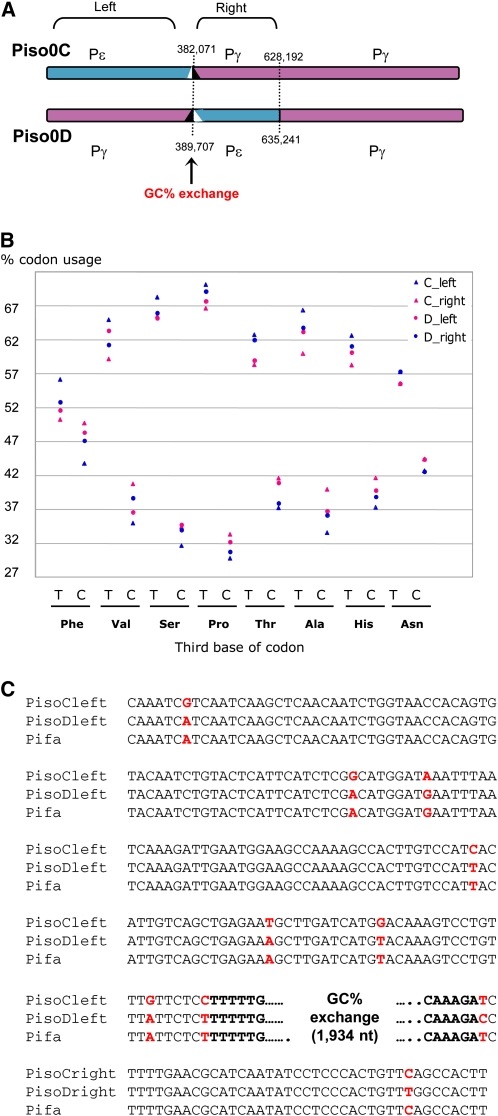
Analysis of the GC trend curve exchange between chr. C and D. (A) Position of the GC exchange determined by the global GC content analysis along chromosomes using a sliding window of 10 kb and a step of 1 kb. (B) Distribution of the codon usage percentages calculated from tRNA species that pair with two codons and showing more than 1.5 variation between both codons (extracted from Table 1, lane Pγ-Pε). Values for each tRNA were calculated for the left and the right regions of the C/D chromosomal exchange, respectively. (C) Multiple alignments of *P. sorbitophila* and *P. farinosa* CBS 2001 sequences around the GC trend curve exchange. This exchange area is characterized by a 100% identical region (1934-nt long) between chr. C and D. Positions of SNPs between *P. sorbitophila* chr. C, chr. D and *P. farinosa* CBS 2001 are indicated in red. As shown, *P. farinosa* chromosomal sequence is first identical to *P. sorbitophila* chr. D sequence and then to chr. C (after the 1934-nt long sequence), confirming that the GC exchange ensues from a reciprocal translocation event.

We finally observed a large exchange between GC% trend curves in the C/D chromosomal pair (Figure 1), with a bias of 2.92% (mean = 2.44 [1.39-4.36]) in the codon usage between upstream and downstream regions (Figure 2). We checked that it was not relevant to a sequence assembly error by confirming both chromosomal sequences using polymerase chain reaction amplifications and resequencing. Thus, the GC trend curve profile suggests that a reciprocal exchange of sequences took place between both chromosomes. The left border of this chromosomal exchange (Figure 2) is located into a putative isoleucine tRNA synthetase coding sequence, with 100% sequence identity between both alleles. Other GC% trend curve exchanges were not taken into account in the following analyses, because they were of limited size and some of them likely corresponded to noise.

#### Pγ subgenome allelic sequences in *P. farinosa* CBS 2001:

A taxonomical study of the *Pichia (Millerozyma) farinosa* group of species (S. Mallet *et al.*, unpublished results) revealed that several sequences from the *P. sorbitophila* Pγ subgenome were nearly 100% identical to the haploid yeast *Pichia farinosa* var. *farinosa* CBS 2001. To confirm the origin of each part of the *P. sorbitophila* genome, we completed this study by sequencing 17 other chromosomal sites in *P. farinosa* CBS 2001 (Table S4). Special attention was paid to cover the E/F/I/J translocation breakpoint as well as the C/D chromosomal exchange and NUMTs (NUclear sequences of MiTochondrial origin) positions (see section *Unequal acquisition of mitochondrial DNA sequences*, below). We observed that all markers in regions defined as belonging to Pγ subgenome shared 99% to 100% identity with *P. farinosa* CBS 2001, whereas all markers in regions attributed to Pε subgenome shared only 90% to 96% identity (Figure 1 and Table S4). Sequencing data for chr. A/B and C/D showed also that their homozygous regions belonged to Pε and Pγ, respectively. As a whole, these results confirm that: (1) the *P. sorbitophila* genome is an admixture of two parental subgenomes (Pγ and Pε); (2) the A/B, C/D, G/H, and K/L pairs were subjected to LOH processes; and (3) a C/D chromosomal exchange took place in the *P. sorbitophila* hybrid genome after its formation. Indeed, SNPs identified between both *P. sorbitophila* subgenomes and the genome of *P. farinosa* CBS 2001 (Figure 2C) are strongly correlated with the GC% trend curves exchange observed for chr. C/D (Figure 2A).

#### Phylogenetic position of Pγ and Pε:

**Figure 3:**
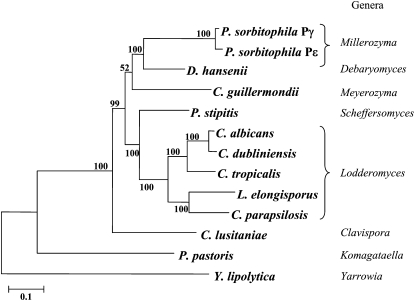
Phylogenetic positions of the two subgenome sequences identified in *P. sorbitophila* hybrid. *Yarrowia lipolytica* is used as outgroup to root the CTG tree. The tree was built from the alignment of 233 protein families (87,181 amino acids per species) having a single member in each analyzed species. Amino acid sequences for each family were aligned with MAFFT (Katoh *et al.* 2009) and cleaned with Gblocks (Talavera and Castresana 2007). The tree was built from the resulting alignment with the maximum likelihood method using PHYML with a JTT substitution model corrected for heterogeneity among sites by a Γ-law distribution using four different categories of evolution rates (Guindon and Gascuel 2003). The proportion of invariable sites and the α-parameter of the Γ-law distribution were optimized according to the data. Bootstraps were calculated from 100 replicates. They are indicated before each node and the scale for branch length at the bottom of the figure.

The sequence identity shared by *P. farinosa* CBS 2001 and Pγ for several positions on the genome suggests that one of the *P. sorbitophila* progenitors belongs to the *Millerozyma* group of species and is closely related to *P. farinosa* CBS 2001. We determined the phylogenetic position of Pγ and Pε in the CTG group of yeasts (Butler *et al.* 2009; Dujon *et al.* 2004; Jackson *et al.* 2009; Jeffries *et al.* 2007; Jones *et al.* 2004) (Table S5), using protein coding genes simultaneously present in both subgenomes (Figure S6). As shown in Figure 3, most of the branches of the phylogenetic tree obtained agree with previously published trees (Butler *et al.* 2009; Kurtzman and Suzuki 2010). Pγ and Pε are positioned in distinct strongly supported branches (bootstrap value = 100), with a phylogenetic distance corresponding to the separation of the species and with *D. hansenii* as the nearest known yeast of the CTG group with a fully sequenced genome. According to recent phylogenetic studies (Kurtzman and Suzuki 2010; S. Mallet *et al.*, unpublished results) Pγ and Pε correspond to two distinct species of the *Millerozyma* (*Pichia) farinosa* group of species.

### Comparison between Pγ and Pε subgenomes

#### Divergence of protein-coding gene alleles:

The genome of *P. sorbitophila* proves to be one of the smallest identified in the CTG group according to its size (10.75 Mb) and the number of annotated genes (5626 protein-coding genes), if we consider the equivalent haploid genome (Table 2 and Table S7). The gene redundancy is limited for both subgenomes compared to *D. hansenii* with a total of 33.2% protein-coding genes belonging to multigene families against 51.5% and three times less tandemly duplicated gene arrays (Table S8). In each chromosomal pair, a protein coding gene is present in most cases in two allelic copies coming either from both parents (in heterozygous regions) or from a sole parent (in homozygous regions), giving a total of 11,252 loci (Table 2 and supporting information). When genes with introns are located in heterozygous regions, both alleles of the genes contain the same number of introns, almost identical in size (Table S6).

**Table 2:** *P. sorbitophila* genomic features in Pγ and Pε subgenomes

Parental Contribution	Chromosomal Region	Total No. ProteinCoding Genes				Total No. Noncoding RNA					Total No. Other Elements NUMTs Loci
		CDS Without Intron	CDS With Introns	Pseudo-Gene	Total Gene	tRNA	snoRNA	snRNA	Pol III ncRNA	Ribosomal DNA*^a^*	
Pγ	Solo	95	2	7	104	0	0	0	0	73	1
	Heterozygous*^b^*	2973	219	13	3205	88	21	3	5	0	0
	Homozygous	3834	246	10	4090	92	26	2	0	0	2
	Total	6902	467	30	7399	180	47	5	5	73	3
Pε	Solo	106	4	6	116	0	0	0	0	0	8
	Heterozygous*^b^*	2950	218	37	3205	88	21	3	5	0	0
	Homozygous	482	46	4	532	20	6	0	0	0	4
	Total	3538	268	47	3853	108	27	3	5	0	12
Total genome		10,440	735	77	11,252	288	74	8	10	73	15
Haplotype equivalent		5,220	367.5	38.5	5626	144	37	4	5	36.5	7.5

CDS, CoDing Sequence; Pol III, polymerase III; NUMTs, NUclear sequences of MiTochondrial origin.

a Tandemly repeated units.

b Loci (3205 pairs) containing both parental genes (a total of 6410 genes). They correspond to CDS/CDS pairs for 3161 pairs, to CDS/pseudogene pairs for 38 pairs, and to pseudogene/pseudogene pairs for 6 pairs (supporting information).

Among the 3425 genes in heterozygous regions, 3205 (93.6%) are represented by two nonidentical coding-alleles (Table 2) demonstrating on average 92.1% of identity at the protein level (Id_prot_) and a mean ratio of nonsynonymous substitutions per synonymous substitutions (dN/dS) of 0.121 (Figure S9). Three subsets of heterozygous genes are particularly interesting. First, genes showing highly conserved alleles (44 genes, dN/dS < 0.0046, Id_prot_> 99.8%) likely encode for essential functions (Table S9). Second, some genes with highly divergent alleles (15 among 90 genes) correspond to “*Millerozyma*-specific genes” found only in Pγ and Pε subgenomes but not in the other yeasts of the CTG group (Table S10). They probably appeared in the Pγ and Pε common ancestor after its separation with the ancestor of *D. hansenii* and are under a relaxed selection pressure. The third subset is composed of 38 CDS-pseudogene allele pairs (CDS or CoDing Sequence is defined here as the region of nucleotides that corresponds to the sequence of amino acids in the predicted protein and that is not interrupted by internal frameshift or stop codon). The allele distribution for these genes is not homogeneous since almost all pseudogene alleles (81.6%) are located in the Pε subgenome whereas the corresponding coding alleles are in the Pγ. Finally, the two groups of genes with highly divergent alleles (90 CDS-CDS pairs and 38 CDS-pseudogene pairs) may constitute an interesting pool of genes probably required for specific adaptations to environmental conditions, since they show bias in Gene Ontology frequencies in favor of transporters, phosphatases, oxidoreductases, and cell wall proteins (Table S11 and Table S12).

Only a limited number of genes (220 genes) in one subgenome has no allelic counterpart in the other subgenome. Among them, 102 correspond probably to “dubious open reading frames” (Figure S8), reducing the number of single allele genes to 118. They are located in three different genomic areas: 83 genes (70.3%) at the end of contigs, 12 genes (10.2%) in the E/F/I/J translocation breakpoint and 23 genes (19.5%) in the heterozygous regions. The distribution of single allele genes between Pγ and Pε is slightly in favor of Pε with 78 genes (66.7%) against 40 for Pγ (33.3%). Despite their small number (2% of the total number of genes), they are at the origin of some metabolic pathways in the *P. sorbitophila* hybrid as described in the section *Unilateral acquisition of genes for sugar degradation*.

#### Uniparental conservation of ribosomal RNA genes:

**Figure 4:**
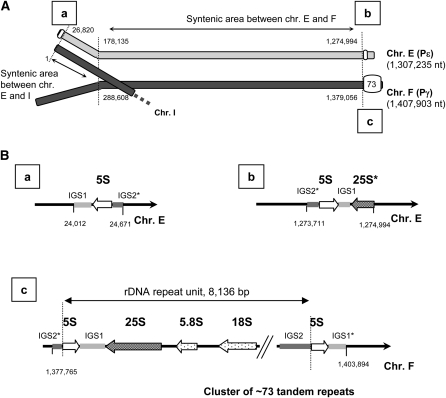
Location and organization of the ribosomal DNA clusters. rDNA sequences were identified by comparison to other yeast genomes (Table S5). Three loci were identified, one at the left border of chr. E (“a” in A and B), a second at the right border of chr. E (“b” in A and B) and a third at the left border of chr. F (“c” in A and B), the latest one containing approximately 73 tandem repeats. (A) Indicates the position and coordinates of each locus on chromosomes. (B) Describes the loci organizations, incomplete elements are indicated by stars. These three organizations were also checked by polymerase chain reaction amplification, end sequencing and PFGE hybridization (Figure S2).

Despite the fact that the *P. sorbitophila* nuclear genome has three different ribosomal DNA (rDNA) loci, the rDNA is at a hemizygous state with a sole cluster of repeats (Figure 4). The latter is located on the right arm of chr. F (Pγ) and contains the 5S and 35S transcript units (precursor of 18S, 5.8S, and 25S RNAs), repeated around 73 times (Figure 4B). At the allelic position on chr. E (Pε, right arm), a whole 5S unit and a deleted 25S unit are present, but without repetition. Finally, another relic of rDNA cluster (a single 5S rDNA locus) is at the opposite left arm of chr. E (Pε). It seems therefore that, after the hybrid formation, the Pε-like parental chromosome (chr. E) underwent an entire loss of the rDNA repeats. The two rDNA relics on chr. E and issued from Pε share 90.3% sequence identity (Figure S10) suggesting that they have already been present in the Pε parental genome before the hybrid formation. These two loci are located in highly polymorphic subtelomeric regions (Figure S11), that could have contributed to the loss of the rDNA repeats. The hemizygous state of the ribosomal DNA contrasts with the distribution of other noncoding RNA genes (ncRNA and tRNA), which are all represented by two well-conserved alleles (see supporting information).

#### Unequal acquisition of mitochondrial DNA sequences:

Most eukaryotic nuclear genomes contain pieces of mitochondrial sequences (Lenglez *et al.* 2010; Sacerdot *et al.* 2008), designated as NUMTs for NUclear sequences of MiTochondrial origin. They result from the transfer of fragments of mitochondrial DNA into chromosomes. The number and size of the NUMTs vary significantly between yeasts within the monophyletic group of hemiascomycetes (Sacerdot *et al.* 2008). For the *P. sorbitophila* nuclear genome, we observed a highly unequal distribution of NUMTs (Figure 1 and Table S3). First, of the 24 NUMTs identified from its extant mitochondrial genome (Jung *et al.* 2009), 20 are exclusively located in the subgenome deriving from the Pε parent. Therefore, most of the NUMTs (14 NUMTs) are at a hemizygous state with the equivalent allelic sequence being devoid of NUMT. Three arguments lead us to propose that the mitochondrial sequences have been inserted into the nuclear genomes of both parents before the hybridization event: (1) all NUMTs in heterozygous regions are at a hemizygous state; (2) the present *P. sorbitophila* hybrid is separated from its ancestral hybrid by only few thousand successive generations (estimated from the polymorphism level in homozygous regions and based on the method described by Rolland and Dujon 2011), whereas the number of NUMTs insertions is equivalent to what observed for other yeast genomes; and (3) the NUMT located on the G/H homozygous pair in two identical alleles is also present in *P. farinosa* CBS 2001 (Table S4), closely related to the Pγ progenitor. The nonuniform distribution of NUMTs reveals also that Pε has undergone four times more mitochondrial DNA insertions than Pγ. The sequence divergence between the *P. sorbitophila* and *P. farinosa* CBS 2001 mitochondrial *COX2* genes suggests that the mitochondrial genome (mtDNA) of *P. sorbitophila* was inherited from the Pε progenitor (see supporting information).

#### Chromosomal rearrangements in parental genomes:

Three distinct chromosomal areas of the *P. sorbitophila* genome were analyzed to identify molecular events at the origin of synteny breaks: (1) E/F/I/J chromosomes, (2) subtelomeric regions, and (3) internal chromosomal positions where single allele genes are located.

Gene orders at the E/F/I/J translocation breakpoint were compared with the orthologous regions identified in *D. hansenii* and *Candida guillermondii* (Figure S12). The fact that the gene orders on chr. I and F (Pγ) are identical or almost identical to those of *D. hansenii* and *C. guillermondii* suggests that the chromosomal arm exchange likely took place between the two other chromosomes, chr. E and J (Pε). The short evolutionary period of *P. sorbitophila* since its formation suggests that this event appeared in Pε before the hybridization event, although we cannot exclude that it was part of the early chromosomal rearrangements occurred in the hybrid genome. The translocation breakpoint is particularly enriched in tandemly duplicated genes. Among the 12 single-allele genes located in chr. E and J, eight are tandemly duplicated and form four distinct tandem gene arrays (Figure S12). Insertion and amplification of those duplicated genes probably either induced the chromosomal exchange or were initiated by the exchange.

The plasticity of subtelomeric regions and their capacity to harbor large gene families make their comparison between subgenomes extremely difficult. However, we found that some of the 83 single allele genes located in subtelomeric regions were the result of gene location movements in parental genomes. An example of such cases is given in Figure S13.

The remaining 23 single allele genes in heterozygous regions are mainly concentrated in 10 internal chromosomal areas (Figure S3). Compared with the corresponding gene order with *D. hansenii*, *C. guillermondii*, *Pichia stipitis*, and *C. albicans*, the single allele genes are missing in all other species at these specific locations whereas the surrounding regions are highly syntenic (Figure S14). Consequently, most of these genes were probably inserted into the corresponding genomic areas in one parental genome after the separation of both parents from their common ancestor. These insertions may result from diverse molecular events: gene location movement as observed in subtelomeric regions, gene acquisition (four single-allele genes are species-specific), gene duplication in cases of multigene families (eight single-allele genes), and tandem duplication as observed for chr. F (Figure S14) or more complex events.

### Acquisition of metabolic pathways

#### Unilateral acquisition of genes for sugar degradation:

Analysis of single-allele genes at the level of their putative encoded functions shows that some key genes for *P. sorbitophila* metabolism have been acquired from the Pε parent. They are still conserved in *P. sorbitophila* hybrid genome, although the Pε subgenome constitutes no more than 32% of the totality. This is noteworthy for maltose degradation (Table 3): *P. sorbitophila* is able to hydrolyze maltose (de Miranda *et al.* 1980) in contrast to *P. farinosa* CBS 2001 (strain closely related to Pγ). The *MAL* genes (Alves *et al.* 2008; Chow *et al.* 1989) are exclusively single allele genes inherited from Pε and exist in several copies at four different areas of synteny break in heterozygous regions: for example, the E/F/I/J translocation breakpoint contains two tandemly duplicated *MALX3* genes on chr. E and two tandemly duplicated *MALX2* genes on chr. J (Figure S12). At a synteny breakpoint between chr. M and N, four of the five additional genes on chr. M are *MAL* genes, with the corresponding gene order *MALX2-MALX1-MALX3* (pseudogene)–*MALX3* (Figure S14). As a whole, the Pε subgenome encodes three *MALX1* permeases, four *MALX2* maltases and three *MALX3* activators whereas Pγ has none (Table 3).

**Table 3:** Distribution of single-allele genes between Pγ and Pε for sugar degradation and other transports

Putative Function	Gene Name	Locus in Pγ	Locus in Pε
Sugar metabolism			
Maltose permease	MALX1		PISO0M16930g
			PISO0J21547g
			PISO0M00166g
Maltase	MALX2		PISO0M00188g
			PISO0M16886g
			PISO0J03551g
			PISO0J03441g
MAL activator	MALX3		PISO0E02028g
			PISO0E02050g
			PISO0M16974g
			PISO0J35007g*^a^*
Invertase	SUC2		PISO0J03639g
			PISO0J03573g
Sorbitol dehydrogenase	SOR1	PISO0N22123g	PISO0M21880g
			PISO0E00180g
		PISO0K00604g/ PISO0L00605*^b^*	
Gluthatione metabolism			
5-oxoprolinase	OXP1	PISO0I02648g	PISO0J04431g
		PISO0C10078g/PISO0D10145g*^b^*	
			PISO0E04690g
			PISO0J03595g
Allantoate transport			
Allantoate permease	DAL5		PISO0A12958g/ PISO0B13025g*^b^*
			PISO0M24916g
			PISO0M17480g
		PISO0K23022g/ PISO0L23023g*^b^*	
			PISO0J21503g
		PISO0I08192g	PISO0J10019g
			PISO0E04646g
Nicotinic acid transport	NTA1	PISO0I02626g	PISO0J04409g
			PISO0J03617g
			PISO0J03485g
		PISO0K00318g/ PISO0L00319g*^b^*	
		PISO0K00406g/ PISO0L11407g*^b^*	
		PISO0N15105g	PISO0M14708g

a Pseudogene.

b Identical alleles located in homozygous regions.

We also observed a bias in favor of the Pε subgenome for the invertase *SUC2* gene (Carlson *et al.* 1983; Taussig and Carlson 1983) with one tandem array of two *SUC2* genes at the translocation breakpoint (Figure S12) and no copies in Pγ subgenome. The Pε subgenome also contributes for seven of the ten allantoate permease genes and the Pγ for three of five sorbitol dehydrogenase genes (Table 3). In conclusion, a limited number of genes have been deleted or acquired in both parents since their separation from their common ancestor. These minor differences may however have significant consequences on *P. sorbitophila* metabolism.

#### Biparental acquisition of genes for stress resistance, mating type, and meiosis:

*P. sorbitophila* was isolated as a contaminant of a 70% sorbitol solution (de Miranda *et al.* 1980). Compared with *S. cerevisiae* and *D. hansenii*, *P. sorbitophila* is more tolerant to NaCl (4M), LiCl (0.8 M), and KCl (2.5 M) (Maresova and Sychrova 2003). Previous studies on Na^+^ intracellular limitation pathways in *P. sorbitophila* allowed the identification of three key transporters: the *NHA1* and *NHA2* cation/H^+^ antiporters (Banuelos *et al.* 2002), which correspond to two alleles of the same gene; the P-type ATPase (Benito *et al.* 2004); and a H^+^/glycerol symport activity (Lages and Lucas 1995). To complete those data, we searched for the presence of more than 50 known genes involved in ion, glycerol, and water transport or in osmotolerance-related pathways (Table S18), identified in *S. cerevisiae* (obtained from Saccharomyces Genome Database, http://www.yeastgenome.org/) and also well documented for *D. hansenii* (Prista *et al.* 2005). We found that, in contrast to the metabolism of sugar degradation, osmotolerance genes were not specific to one subgenome but rather identified with their two related coding alleles in heterozygous regions.

To achieve Na^+^ efflux, several *ENA* genes (“Exitus NAtru” genes) encoding Na^+^-ATPases are found in *D. hansenii* and *S. cerevisiae* (Benito *et al.* 2004), whereas only one *ENA* gene has been identified in *P. sorbitophila*. The latter also lacks the *HAL1* gene (Rios *et al.* 1997), which decreases intracellular Na^+^ via *ENA1*. Potassium is the most abundant intracellular cation in living cells and plays important roles in biological processes. The *NHA1-2* Na^+^/H^+^ antiporter is not specific for Na^+^ but also mediates K^+^ efflux in addition to Na^+^, allowing a possible intracellular K^+^ depletion (Benito *et al.* 2004). In *P. sorbitophila*, this depletion may be intensified by the presence of *TOK1* (Table S18) a permeable channel for potassium efflux (Ketchum *et al.* 1995; Rios *et al.* 1997) missing in *D. hansenii*. To counterbalance the K^+^ efflux at high NaCl concentrations, it has been proposed that an efficient K^+^ uptake system must exist in *P. sorbitophila* (Banuelos *et al.* 2002). We identified the two potassium transporters *HAK1* (high-affinity K transporter) and *TRK1* (TRansport of K) and the P-type ATPase *ACU1* (Benito *et al.* 2004). *P. sorbitophila* lacks the *PHO89* Na^+^/Pi cotransporter (Martinez and Persson 1998) in contrast to *D. hansenii*, which has *PHO89* but not *ACU1*. Osmoregulation is also achieved by the production of glycerol or other osmolytes (*e.g.*, arabitol, erythritol) and the capacity to maintain them into the cells (Kayingo *et al.* 2001; Lages and Lucas 1995; Lages *et al.* 1999). Glycerol leaks through the plasma membrane and its retention therefore needs an active transport system. In *P. sorbitophila*, H^+^/glycerol symport allows the intracellular accumulation of glycerol (Lages and Lucas 1995) and we also show that the aquaglyceroporin *FPS1*, the glycerol permease responsible for glycerol leakage (Tamás *et al.* 1999), is missing.

As for stress resistance, *P. sorbitophila* genes involved for mating, meiosis, and spore formation are in two allelic versions, suggesting a conservation of these genes from the common ancestor of Pγ and Pε progenitors until the present hybrid. In *P. sorbitophila*, we found two similar mating-type loci at the heterozygous allelic positions in chr. M and N (Figure S15). Both contain *MATa2* and *MATalpha1* genes but lack *MATa1* and *MATalpha2*. Thus, as *P. stipitis* and *D. hansenii* (Butler 2010; Fabre *et al.* 2005; Jeffries *et al.* 2007; Lee *et al.* 2010), *P. sorbitophila* has its mating-type loci fused with no *MATalpha2*. *MATa1* is missing only in *P. sorbitophila*, a gene loss that likely took place in the common ancestral species of both parents. We also searched for the presence of 227 orthologs of *S. cerevisiae* genes involved for mating, meiosis and spore formation since key genes for the sexual development and meiosis in *S. cerevisiae* are missing in *Candida* species (Butler *et al.* 2009), and we also revisited the gene annotations of the 2^nd^ version of the *D. hansenii* genome (Souciet *et al.* 2009). All genes identified in *D. hansenii* (187 genes) were detected in two copies in *P. sorbitophila* (Table S19). Three additional genes are present in *P. sorbitophila*: *DIT1* and *DIT2* involved in dityrosine synthesis (Briza *et al.* 1994) and *YEL023C* which may participate in this process (Butler *et al.* 2009). Dityrosine is the major component of the outer layer of spore wall. It forms an insoluble scaffold on the surface of the spore (Briza *et al.* 1988, 1990) and contributes to its resistance. There are no data available for spore resistance concerning *P. sorbitophila*. However, we can speculate that the presence of these three genes may contribute to the resistance of spores in specific environmental conditions.

## Conclusion

Allopolyploid hybrids, which are the result of cell fusions between different species, undergo generally rapid and extensive genomic modifications after their formation (Rainieri *et al.* 2006; Sipiczki 2008). With the aim of deciphering hybrid genome evolution, these posthybridization rearrangements have to be distinguished from those which took place in the parental genomes during the long period that preceded the hybrid formation. The complete sequencing of the genome of the osmotolerant yeast *P. sorbitophila* strain CBS 7064 (de Miranda *et al.* 1980) revealed that it is a hybrid genome. Despite the fact that no complete genome sequence closely related to that of one or both parents is available so far, a detailed analysis of its genome led us to retrace its evolution. The *P. sorbitophila* nuclear genome (21.5 Mb) is actually composed of seven pairs of chromosomes issued from two progenitors named Pγ and Pε, with Pγ being closely related to *P. farinosa* CBS 2001. In contrast, the origin of the Pε parent remains unknown because of the lack of close sequence similarity among existing yeasts. The sequence divergence observed between both subgenomes (10.84% at the nucleotide level between syntenic regions) is equivalent to the one described between the genomes of *S. cerevisiae* and *S. paradoxus*, two distinct species of the genus *Saccharomyces* (Cliften *et al.* 2001).

In addition, it reflects the long evolutionary period that separates the Pγ and Pε parents from their common ancestor. During this long evolutionary period, the genomes of both parents underwent very few genomic reshaping events: some gene acquisitions and gene location movements, rare gene duplications, differential levels of NUMTs insertions and probably one chromosomal arm translocation, allowing conservation of large syntenic gene blocs. However, these events might have been decisive for the *P. sorbitophila* hybrid evolution and adaptability, as shown by the uniparental acquisition of *MAL* and *SUC* genes. At the opposite, genes involved in osmotic stress resistance or spore resistance were already acquired by the Pγ and Pε common ancestor. For these classes of genes, heterosis might still result from interaction between distinct alleles. *P. sorbitophila* offers therefore a unique case to study acquisitions of novel functional properties originating from the admixture of the parental genetic contributions.

**Figure 5:**
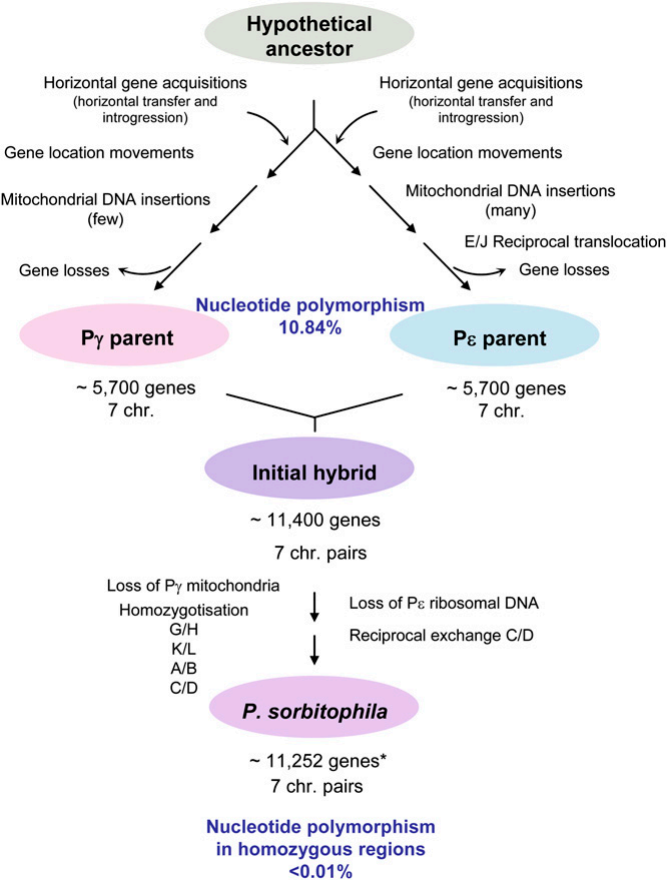
Genomic reshapings identified since the separation of Pγ and Pε parents from their common ancestor to the current *P. sorbitophila* genome. Position of rearrangements in each part of the flowchart (before or after hybridization) is not relevant of their chronology. (*) The 11,252 genes in *P. sorbitophila* are in fact 3205 gene pairs coming from both parents, 2045 pairs coming from only Pγ parent, 266 pairs from Pε parent, and 116 and 104 are single-copy genes derived from either Pγ or Pε, respectively.

The *P. sorbitophila* genome also provides an interesting snapshot of the genomic evolutionary events after an interspecific hybridization in eukaryotes (as summarized in Figure 5). On one hand, we observed a diploid level for this genome, in contrast to aneuploid situations reported for hybrids of the genus *Saccharomyces* (Querol and Bond 2009), probably attributable to the hybridization event that generated directly a strict allodiploid hybrid (Gerstein *et al.* 2006). On the other hand, LOH appears to play a prominent role. In total, 40.3% of this genome has only one parental origin (35.5% from Pγ and 4.8% from Pε). For all concerned chromosomes, LOH extends up to telomeres, a phenomenon also observed in *Candida* species (Butler *et al.* 2009; Diogo *et al.* 2009; Forche *et al.* 2005). In the partly homozygotized chromosomal pairs, LOH origin ignores gene borders. The limited but existing sequence polymorphism observed between pairs of homozygotized regions indicates that at least four successive LOH events took place in this hybrid. Assuming the same mutational rate as in *S. cerevisiae* and considering that the few mutational changes were essentially neutral in homozygotized regions, we can estimate that LOH process started only few thousand generations ago (estimated at nearly 185,000 generations from method described in Rolland and Dujon 2011), and that the formation of the hybrid was just anterior to the beginning of this process. According to Fay and Benavides (2005) and to Rolland and Dujon (2011), we can speculate that the hybrid formation occurred in the last centuries. This estimated time is consistent with the fact that *P. sorbitophila* was isolated from a manufacturing product (a highly concentrated sorbitol solution).

In contrast to the extent of LOH, uniparental gene loss played a limited role during the evolution of the *P. sorbitophila* genome, with the notable exceptions of rDNA (inherited solely from Pγ) and mtDNA (inherited from Pε). The unilateral loss of rDNA was also observed in other hybrid eukaryotic genomes. In the allotetraploid grass *Zingeria trichopoda*, the *Z. biebersteiniana* like parental chromosomes would have undergone a massive loss of 45S rDNA (Kotseruba *et al.* 2003). In the lager brewing yeast *S. pastorianus*, the rDNA from the *S. cerevisiae*-type subgenome is approximately 20 times more represented than its *S. bayanus*-type counterpart (Nakao *et al.* 2009), whereas both of the parental rDNA types were retained in the *Zygosaccharomyces* allopolyploid (Gordon and Wolfe 2008). The consequence of this unilateral loss, that is, the transcription of rDNA genes inherited from a sole parent, is comparable with the nucleolar dominance observed in numerous plant interspecific hybrids. In this latter case, rDNA loci inherited from both parents are conserved but rDNA genes derived from one progenitor are silent (Lewis *et al.* 2007; Pikaard 2000). These observations raise the problems of the origin of the rDNA instability and the viability of meiotic products. Our preliminary results of sporulation experiments show that *P. sorbitophila* is able to produce asci containing one to four ascopores, as observed previously by de Miranda *et al.* (1980) and Oliveira *et al.* (1996). Results also suggest that at most two spores per ascus are viable (data not shown). These preliminary results could be consistent with the hemizygous state of the rDNA if we assume that *P. sorbitophila* is able to undergo meiosis, a hypothesis that requires additional data to be confirmed.

The reconstruction of the complete parental subgenomes allowed us to decipher the recent *P. sorbitophila* genome history and, therefore, to depict, gene by gene, how two divergent genomes put together into a viable hybrid are rearranged during the process of genome stabilization. Our results also show that interspecies hybrids, because of poor prezygotic barrier, are widespread in the *Saccharomycotina* group of species. Human activities in industrial contexts provide unusual substrates (70% sorbitol, for example) that may act as bottlenecks for the selection of particularly resistant species of yeasts and fungi or hybrids. This was probably the case for *P. sorbitophila*. Its genome analysis gave us therefore the opportunity to highlight, in the present times, the early steps of genome evolution after the formation of a hybrid.

## Supplementary Material

Supporting Information
