# Supplementary material for: Pichia sorbitophila, an Interspecies Yeast Hybrid, Reveals Early Steps of Genome Resolution After Polyploidization
Source: G3 (Bethesda). 2012 Feb 1;2(2):299–311. doi: 10.1534/g3.111.000745 (PMC3284337; doi:10.1534/g3.111.000745)
Supplement: Supporting Information [file supp_2_2_299__index.html]

Supporting Information 

# *Pichia sorbitophila*, an Interspecies Yeast Hybrid, Reveals Early Steps of Genome Resolution After Polyploidization

## Supporting Information for Leh Louis *et al.*, 2012

**Files in this Data Supplement:**

- Figure S1 - Sequencing coverage (X) along scaffolds (PDF, 600 KB)
- Figure S2 - Pulse-field gel electrophoresis profile of *Pichia Sorbitophila* chromosomes (PDF, 321 KB)
- Figure S3 - Nucleotide sequence identity along chromosomes (PDF, 1.1 MB)
- Figure S4 - Proposed positions of centromeres (PDF, 1.4 MB)
- Figure S5 - Bias in codon usage between P*γ* and P*ε* subgenomes(PDF, 1.4 MB)
- Figure S6 - Flowchart for the prediction and the annotation of each chromosomal feature in *P. sorbitophila* genome (PDF, 936 KB)
- Figure S7 - Splicing pattern determined for spliceosomal introns detected in *P. sorbitophila* genome (PDF, 361 KB)
- Figure S8 - Flowchart for gene analyses and comparisons (PDF, 867 KB)
- Figure S9 - Distribution of dN/dS values according to the protein sequence identity (PDF, 445 KB)
- Figure S10 - Comparison of 5S rDNA sequences (PDF, 116 KB)
- Figure S12 - Comparison of synteny maps at the E/F/I/J reciprocal translocation between CTG yeasts (PDF, 1.9 MB)
- Figure S11 - Synteny conservation around the rDNA clusters located on the left arm of P. sorbitophila chr E and F (PDF, 727 KB)
- Figure S13 - Gene location movement between two subtelomeric regions (PDF, 538 KB)
- Figure S14 - Comparison of synteny maps at single allele gene positions between CTG yeasts (PDF, 3.8 MB)
- Figure S15 - Comparative organization of the MTL loci in *P. sorbitophila, D. hansenii* and *P. stipitis* (PDF, 429 KB)
- Table S1 - Heterozygous and homozygous parts of *P. sorbitophila* genome (PDF, 86 KB)
- Table S2 - Sequence polymorphism in homozygous regions of the *P. sorbitophila* genome (PDF, 72 KB)
- Table S3 - NUMTs in the nuclear genome of *Pichia sorbitophila* (PDF, 98 KB)
- Table S4 - Sequence identity between *P. farinosa* CBS 2001 and *P. sorbitophila* subgenomes (PDF, 116 KB)
- Table S5 - Databases for yeasts species used in this study (PDF, 70 KB)
- Table S6 - Distribution of introns in protein-coding genes (PDF, 68 KB)
- Table S8 - Characteristics of tandemly duplicated genes arrays (PDF, 81 KB)
- Table S14 - tDNA numbers per chromosome in *P. sorbitophila* (PDF, 79 KB)
- Table S9 - Gene ontology categories for conserved alleles (PDF, 97 KB)
- Table S10 - Hypervariable alleles in heterozygous regions (PDF, 116 KB)
- Table S11 - Gene ontology categories for hypervariable alleles (PDF, 74 KB)
- Table S12 - Gene ontology categories for pseudogenes (PDF, 74 KB)
- Table S13 - Gene ontology categories for single allele genes (PDF, 74 KB)
- Table S15 - Pairs of potentially co-transcribed tRNA genes in *P. sorbitophila* (PDF, 78 KB)
- Table 16 - Codon and tRNA gene usages in *P. sorbitophila* and *D. hansenii* (PDF, 78 KB)
- Table S17 - List of ncRNA genes (PDF, 92 KB)
- Table S18 - *P. sorbitophila* genes in osmotic stress(PDF, 87 KB)
- Table S19 - Conservation of mating and meiosis genes (PDF, 126 KB)
- Table S7 - Comparison of P. sorbitophila global genome features with other yeasts (PDF, 82 KB)
- Supporting Information - Supporting Data, Figures S1-S15 and Tables S1-19 (.zip, 11.8 MB)
