# Supplementary material for: Pichia sorbitophila, an Interspecies Yeast Hybrid, Reveals Early Steps of Genome Resolution After Polyploidization
Source: G3 (Bethesda). 2012 Feb 1;2(2):299–311. doi: 10.1534/g3.111.000745 (PMC3284337; doi:10.1534/g3.111.000745)
Supplement: Supporting Information [file supp_2.2.299_FigureS1.pdf]

1. Structure of the *Pichia sorbitophila* nuclear genome

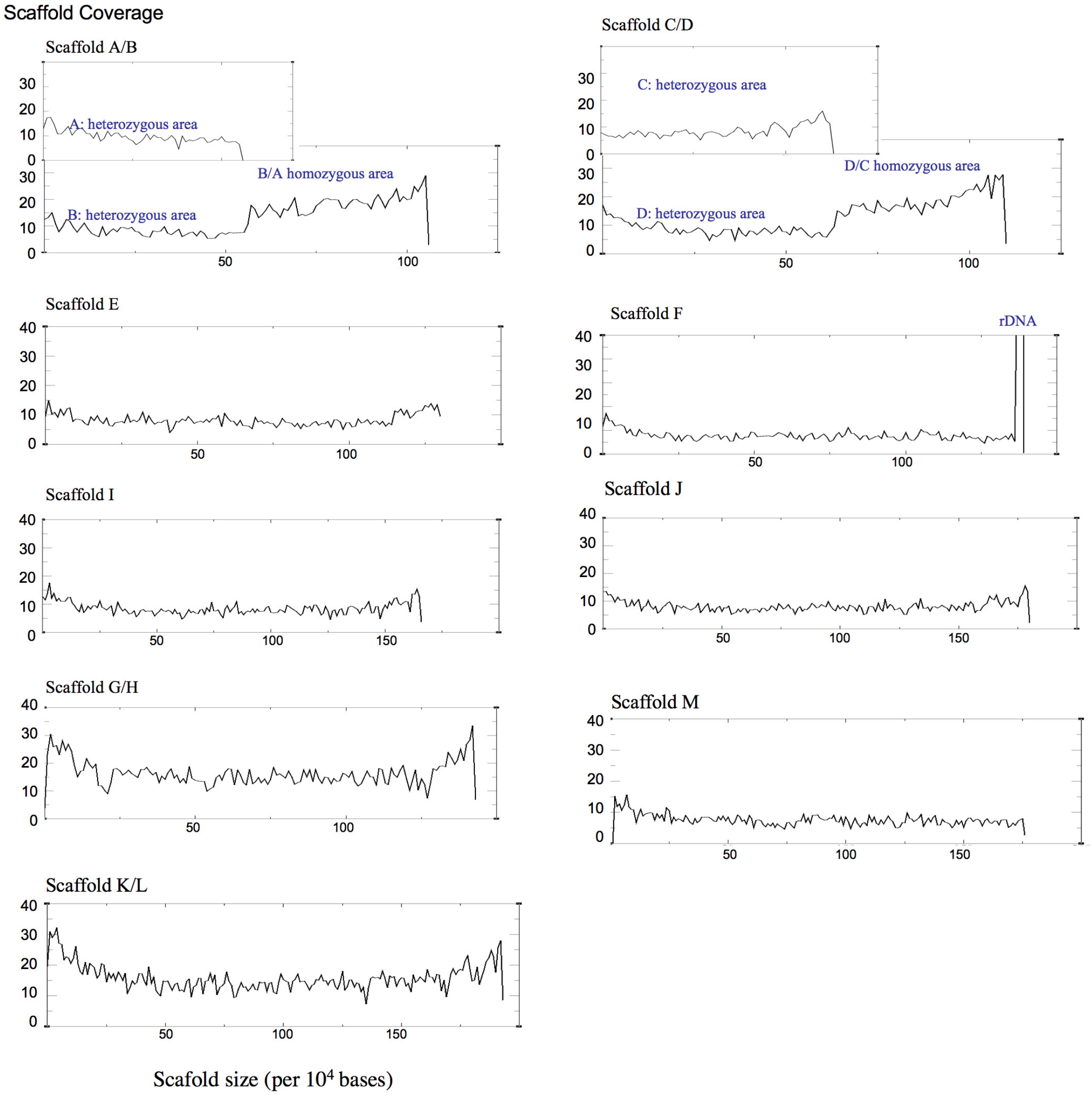

Figure S1 Sequencing coverage (X) along scaffolds. Sequencing depth (Y-axe) was calculated along finished scaffolds (X-axe) by mapping initial Sanger reads on scaffolds. The 11 contigs (7.5X covered) obtained before finishing (see Methods) correspond to the heterozygous regions in A, B, C, D, and to the heterozygous chromosomes E, F, I, J, M, and N (2 contigs), respectively. The six 14X covered contigs correspond to the homozygous parts in A/B, and C/D and to the homozygous chromosomes G/H (2 contigs) and K/L (2 contigs).
