# Supplementary material for: Pichia sorbitophila, an Interspecies Yeast Hybrid, Reveals Early Steps of Genome Resolution After Polyploidization
Source: G3 (Bethesda). 2012 Feb 1;2(2):299–311. doi: 10.1534/g3.111.000745 (PMC3284337; doi:10.1534/g3.111.000745)
Supplement: Supporting Information [file supp_2.2.299_FigureS2.pdf]

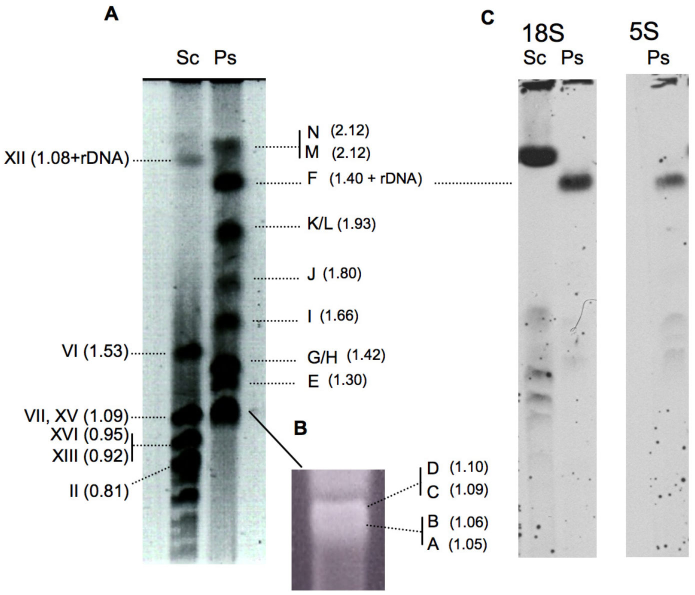

Figure S2 Pulse-field gel electrophoresis profile of *Pichia sorbitophila* chromosomes. Chromosomal DNA was prepared as described previously by Vézinhet *et al.* (1990). Pulsed-field gel electrophoreses were performed using a CHEF Mapper™ pulsed-field electrophoresis system (Bio-Rad), in 1% pulsed field certified agarose (BioRad) and 0,5xTBE (45 mM Tris-borate, 1 mM EDTA). (A) Separation of *P. sorbitophila* (*Ps*) and *S. cerevisiae* (*Sc*) chromosomes with a linear gradient (run time 60h, switch time 250s, angle 106°, voltage gradient 4V/cm) reveals eight bands for *P. sorbitophila*. Name and size (in Mb) of chromosomes (for *Sc*) or scaffolds (for *Ps*) are mapped at the left and the right for *Sc* and *Ps*, respectively. Band intensities are in accordance with the obtained number of *Ps* contigs of equivalent size and which are typically associated in pair: for example, the G and H contigs, identical in size and sequence, correspond to a pair of homozygous chromosomes that comigrate. By comparison, I and J contigs correspond to an heterozygous pair of chromosomes differing in size. (B) A/B and C/D pairs were separated using the following migration conditions: run time 48h, switch time 400s, angle 106°, voltage gradient 3V/cm. (C) Hybridization of chromosomes after transfer onto Hybond N+ membrane (MP biomedicals) and using 5S and 18S rDNA probes amplified from *Ps* and *Sc* genomes. Results show that rDNA repeats are only detected on contig F and confirm the hemizygous state of the rDNA locus.

Additional Note : The size dissimilarity observed between chr. E and F, around 440 kb, is widely due to the absence of an equivalent rDNA repeated cluster on chr. E. This explains why previous hybridization data obtained by Suzuky *et al*. (2003) on *P. sorbitophila* CBS 7064 chromosomes, showed two bands at 1.37 and 1.90 Mb respectively, using a *URA3*-specific probe. They detected therefore both alleles of the gene located respectively on chr. E and F. One can notice that both *URA3* allelic sequences previously published (accession numbers AB109042 and AB109043) are identical to the ones obtained in this study.
