# Supplementary material for: Pichia sorbitophila, an Interspecies Yeast Hybrid, Reveals Early Steps of Genome Resolution After Polyploidization
Source: G3 (Bethesda). 2012 Feb 1;2(2):299–311. doi: 10.1534/g3.111.000745 (PMC3284337; doi:10.1534/g3.111.000745)
Supplement: Supporting Information [file supp_2.2.299_FigureS3.pdf]

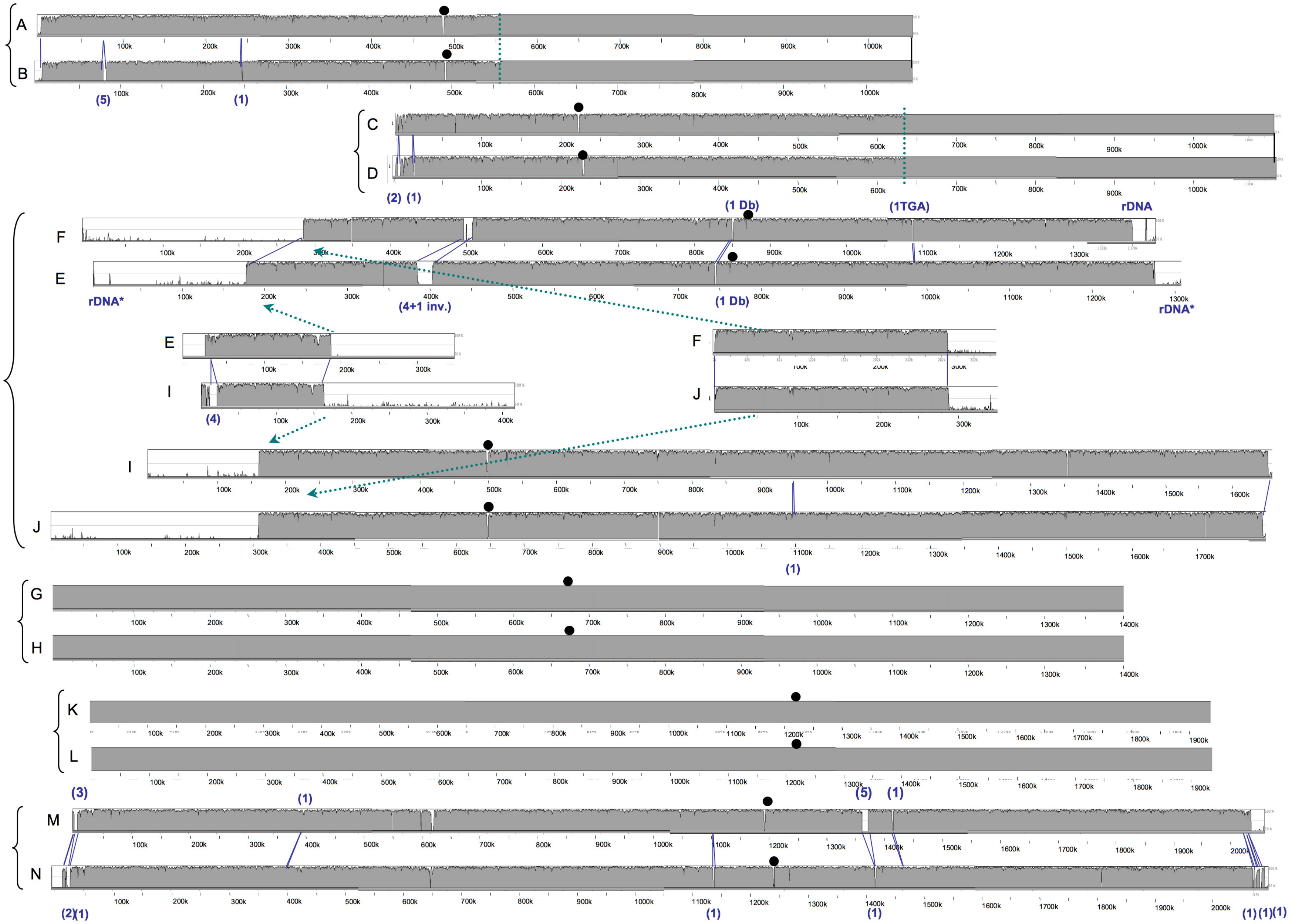

A
B
(5)
(1)
C
D
(2) (1)
(1 Db)
(1TGA)
rDNA
F
E
rDNA*
(4+1 inv.)
(1 Db)
rDNA*
E
I
(4)
F
J
I
J
(1)
G
H
K
L
(3)
(1)
(5)
(1)
M
N
(2)(1)
(1)
(1)
(1)(1)(1)

Figure S3 Nucleotide sequence identity along chromosomes. Pairs of chromosomes were aligned with LAGAN (Brudno *et al.*, 2003) using the global pairwise alignment option and default parameters. Variations of identity (from 50% to 100%) were visualized with the graphical option provided by LAGAN. Switch of identity between chromosomes E/F/I/J, or transition between heterozygous and homozygous regions in A/B and C/D are indicated by green arrows and lines. Positions of rDNA clusters, degenerated (*) or not, are also indicated. Identity and gap percentages were recomputed from the global alignments using a sliding window of 5kb and a step of 1kb to define positions of identity drops (< 70% identity). These positions were then manually curated for the presence of protein coding genes or other genomic elements. Areas of identity drop range from 2.2 kb to 18.5 kb. They correspond either to putative centromere positions (•), sequence variations in intergenic regions or local synteny losses. In the latter cases (14 cases) the number of non syntenic genes is indicated in brackets below the concerned chromosomes. 39 protein-coding genes are concerned, out of which 16 genes are closed to the chromosome ends and can be associated to the 67 other genes located in subtelomeric regions. The remaining 23 genes are located in internal regions. (TGA). The additional gene identified on chr. F is the result of a tandem gene duplication leading to an array of two genes on chr. F against 1 gene on chr. E. (inv.) inversion, the two allelic genes are not in the same orientation (see also Supp. Fig. 14). (Db) dubious ORF.
