# Supplementary material for: Pichia sorbitophila, an Interspecies Yeast Hybrid, Reveals Early Steps of Genome Resolution After Polyploidization
Source: G3 (Bethesda). 2012 Feb 1;2(2):299–311. doi: 10.1534/g3.111.000745 (PMC3284337; doi:10.1534/g3.111.000745)
Supplement: Supporting Information [file supp_2.2.299_FigureS4.pdf]

A

Proposed positions for centromeres

chromosome	Centromere					global_GC%*	Upstream_region		Downstream_region	
	start	end	length	area	local_GC%		upstream_gene	upstream_locus	dowstream_gene	downstream_locus
Piso0A	483754	486340	2587	he	31.117	41.726	Piso0_000278	PISO0A05830g	Piso0_000279	PISO0A05852g
Piso0B	491886	494328	2443	he	30.372	41.362	Piso0_000278	PISO0B05897g	Piso0_000279	PISO0B05919g
Piso0C	222931	225224	2294	he	28.640	40.458	Piso0_000725	PISO0C02686g	Piso0_000726	PISO0C02708g
Piso0D	230388	232657	2270	he	29.031	40.548	Piso0_000725	PISO0D02753g	Piso0_000726	PISO0D02775g
Piso0E	749453	751963	2511	he	30.506	41.356	Piso0_001590	PISO0E08386g	Piso0_001591	PISO0E08408g
Piso0F	850966	853576	2611	he	30.601	41.741	Piso0_001590	PISO0F09729g	Piso0_001591	PISO0F09751g
Piso0G	660324	663504	3181	hm	30.934	41.547	Piso0_003261	PISO0G08432g	Piso0_003262	PISO0G08454g
Piso0H	660324	663504	3181	hm	30.934	41.547	Piso0_003261	PISO0H08433g	Piso0_003262	PISO0H08455g
Piso0I	501951	504862	2912	he	30.529	41.534	Piso0_002232	PISO0I05728g	Piso0_002233	PISO0I05772g
Piso0J	644011	646788	2778	he	31.965	40.854	Piso0_002232	PISO0J07533g	Piso0_002233	PISO0J07555g
Piso0K	1216294	1218822	2529	hm	30.645	41.983	Piso0_004330	PISO0K14508g	Piso0_004331	PISO0K14530g
Piso0L	1216294	1218822	2529	hm	30.645	41.983	Piso0_004330	PISO0K14508g	Piso0_004331	PISO0K14530g
Piso0M	1227373	1230078	2706	he	29.527	40.791	Piso0_005451	PISO0M14972g	Piso0_005451	PISO0M14994g
Piso0N	1246461	1249237	2777	he	31.761	41.299	Piso0_005451	PISO0N15369g	Piso0_005451	PISO0N15391g

* global GC content is calculated for the full lenght chromosome

B

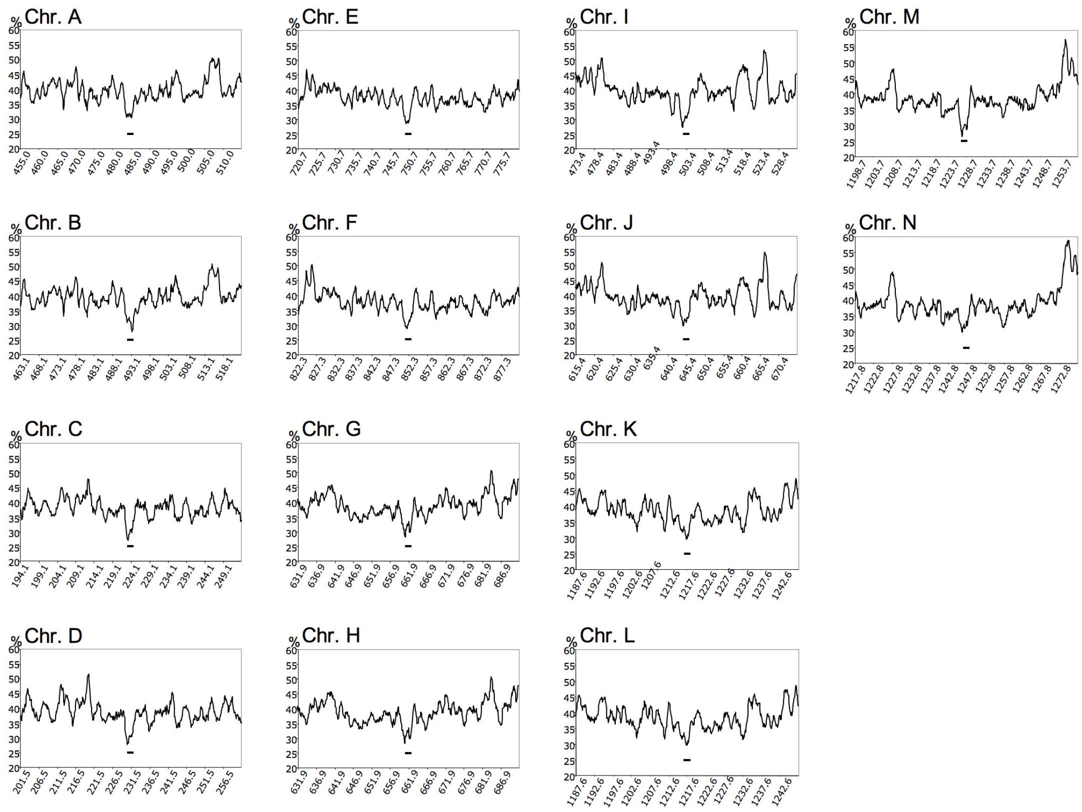

Figure S4 Proposed positions of centromeres. GC content along chromosomes was calculated for sliding windows of 1kb using a step of 0.1kb between successive windows. Windows having a GC content value inferior to two fold the standard deviation value determined for the corresponding chromosome, and located at syntenic position for chromosomes forming a pair were retained. For each chromosome, a unique area, devoid of gene, was obtained. (A) Position of centromeres, GC content and flanking genes. (B) GC content variation in a window size of 60 kb around the centromere. Poor GC area are indicated by lines. X-axe: chromosomal coordinates, Y-axe GC%.
