# Supplementary material for: Pichia sorbitophila, an Interspecies Yeast Hybrid, Reveals Early Steps of Genome Resolution After Polyploidization
Source: G3 (Bethesda). 2012 Feb 1;2(2):299–311. doi: 10.1534/g3.111.000745 (PMC3284337; doi:10.1534/g3.111.000745)
Supplement: Supporting Information [file supp_2.2.299_FigureS5.pdf]

A

Heterozygous regions

amino acid	codon	usage % A Pγ	B_Pε	E_Pε	F_Pγ	I_Pγ	J_Pε	M_Pε	N_Pγ
Phe	TTT	50.25	53.70	52.15	50.50	49.69	53.43	54.02	51.22
	TTC	49.75	46.30	47.85	49.50	50.31	46.57	45.98	48.78
Val	GTT	60.88	61.93	60.29	59.77	59.82	61.14	61.95	59.47
	GTC	39.12	38.07	39.71	40.23	40.18	38.86	38.05	40.53
Ser	TCT	63.58	65.46	65.97	64.59	64.24	65.97	66.54	64.48
	TCC	36.42	34.54	34.03	35.41	35.76	34.03	33.46	35.52
Pro	CCT	66.86	68.31	66.44	65.32	66.49	67.90	68.02	66.54
	CCC	33.14	31.69	33.56	34.68	33.51	32.10	31.98	33.46
Thr	ACT	58.47	61.85	58.80	57.47	57.71	60.64	61.29	58.95
	ACC	41.53	38.15	41.20	42.53	42.29	39.36	38.71	41.05
Ala	GCT	62.81	64.31	61.67	60.23	60.92	64.11	63.42	61.21
	GCC	37.19	35.69	38.33	39.77	39.08	35.89	36.58	38.79
His	CAT	59.18	59.42	58.47	58.10	57.77	60.19	60.48	58.92
	CAC	40.82	40.58	41.53	41.90	42.23	39.81	39.52	41.08
Asn	AAT	53.54	55.35	52.52	52.29	53.01	55.69	55.86	54.24
	AAC	46.46	44.65	47.48	47.71	46.99	44.31	44.14	45.76

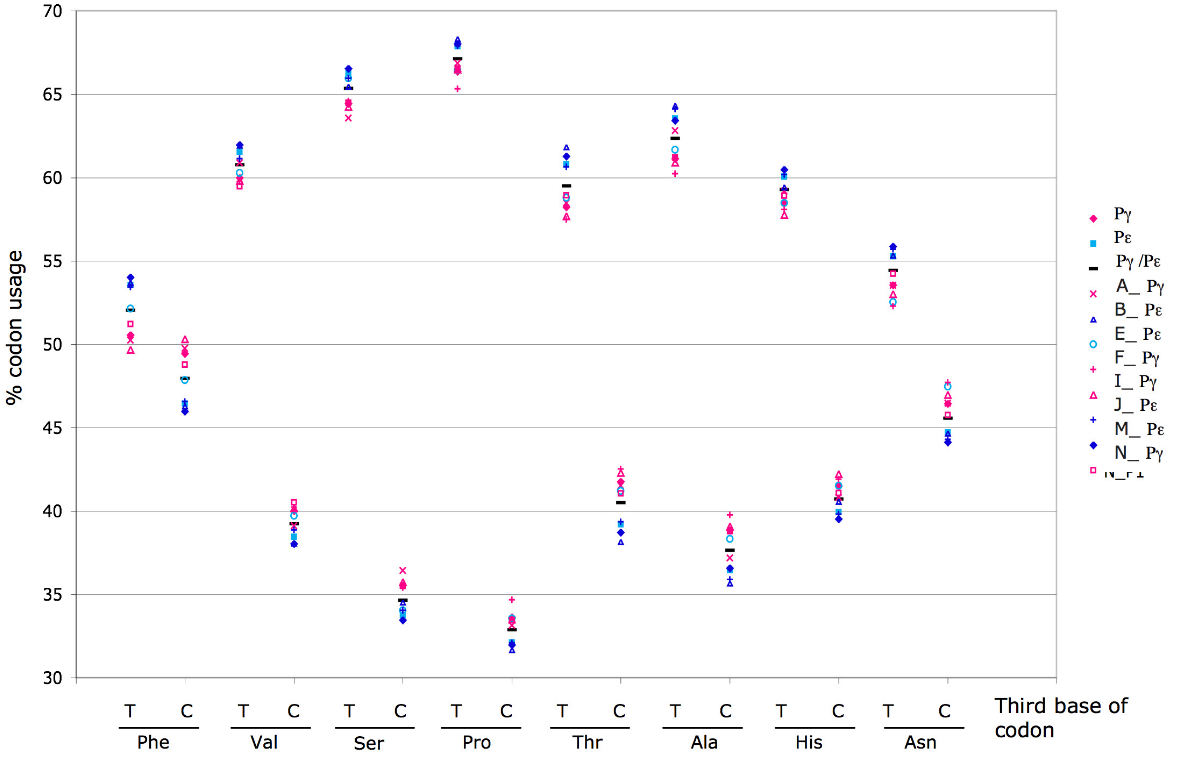

Figure S5 Bias in codon usage between Pγ and Pε subgenomes. For tRNA species that pair with two codons (Crick, 1966), the usage % of each codon was determined as follows: (number of one codon/number of both codonx100). Codon usage % for tRNA species showing more than 1.5 variation between both codons (Table 2) were calculated for each chromosomal region, independently of its origin. The usage % values and their distribution are shown for heterozygous (panel A) and homozygous (panel B) regions. Pγ/Pε corresponds to the mean value between both parents.

B

Homozygous regions

amino acid	codon	usage % A/B	C/D	G/H	K/L
Phe	TTT	50.68	51.12	51.04	49.46
	TTC	49.32	48.88	48.96	50.54
Val	GTT	60.32	61.20	59.10	59.47
	GTC	39.68	38.80	40.90	40.53
Ser	TCT	64.72	65.80	64.17	62.92
	TCC	35.28	34.20	35.83	37.08
Pro	CCT	63.08	68.50	65.07	64.52
	CCC	36.92	31.50	34.93	35.48
Thr	ACT	60.07	59.35	56.09	56.39
	ACC	39.93	40.65	43.91	43.61
Ala	GCT	61.63	63.05	60.87	59.19
	GCC	38.37	36.95	39.13	40.81
His	CAT	56.50	58.84	57.97	55.90
	CAC	43.50	41.16	42.03	44.10
Asn	AAT	51.71	53.93	52.77	52.19
	AAC	48.29	46.07	47.23	47.81

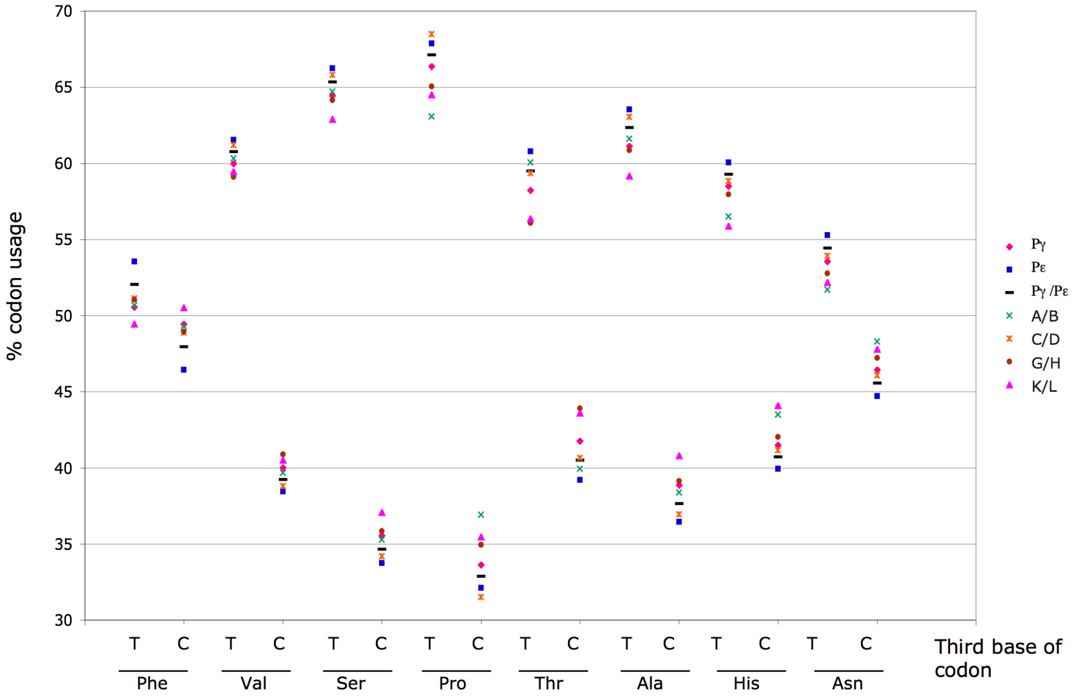

C

Average value of dGC (Fig. 1)

	dGC_Pγ*	dGC_Pε	s**
A/B	1.007	0.993	0.012
C/D_Left$	1.008	0.992	0.012
C/D_Right$	1.005	0.995	0.013
F/JE	1.005	0.995	0.011
I/EJ	1.006	0.994	0.011
M/N	1.006	0.994	0.012
**average**	**1.006**	**0.994**	**0.012**

* Average value of dGC for each heterozygous region.
**Standard deviation for each heterozygous region
$ For the C/D chromosome pair, values refer to regions located on both sides of the reciprocal exchange.

Figure S5 (next). Bias in codon usage between Pγ and Pε subgenomes.
