# Supplementary material for: Pichia sorbitophila, an Interspecies Yeast Hybrid, Reveals Early Steps of Genome Resolution After Polyploidization
Source: G3 (Bethesda). 2012 Feb 1;2(2):299–311. doi: 10.1534/g3.111.000745 (PMC3284337; doi:10.1534/g3.111.000745)
Supplement: Supporting Information [file supp_2.2.299_FigureS6.pdf]

## 2. Analyses of protein coding genes

Protein coding gene models and spliceosomal introns were predicted and annotated using the same procedure as that used for the protoploid yeast genomes (Souciet *et al*., 2009). Additional steps and annotations were introduced for *P. sorbitophila* because of its hybrid status (Fig. S6). In each chromosomal pair, a protein coding gene is present in most cases in two copies coming either from both parents (in heterozygous regions) or from a sole parent (in homozygous regions). Allelic pairs were determined according to the synteny and homology (Fig. S6). As a result, 5,736 genes were identified in the genome from the 11,252 annotated loci. They are represented by two coding alleles for 5,465 genes, one coding allele and one pseudogene allele for 38 genes, two pseudogene alleles for 13 genes, a single coding allele for 207 genes and a single pseudogene allele for 13 genes. Spliceosomal introns were predicted in 735 gene alleles (Table S6). Most of these alleles contain only one intron (685 alleles) but multi-intronic gene alleles were also detected with up to 4 introns per gene (Table S6) leading to a total of 803 introns. Intron structure is very similar to that of *D. hansenii* (Bon *et al*., 2003) with a mean length of 142 nt and a short distance of about 5 nt between branch point (BP) and 3'-splice site (Fig. S7). Sequences at intron boundaries are highly conserved. The main 5'-splice site (5'ss) motif is GTAWGT with GTAAGT presents in 45.8% of the introns and GTATGT in 42.1% (Fig. S7). The consensus motif for the BP is TACTAAC as in *S. cerevisiae* (Lopez and Séraphin, 1999). In heterozygous regions, we observed that both alleles of a gene (182 genes) contain the same number of introns, almost identical in size. Nucleotide variations in introns between alleles (differences of up to 20 nt in size) correspond to internal insertions, mainly in S1.

Protein-coding genes were sorted out according to their location, the sequence divergence between alleles, the number of paralogs and the associated GO-term, using tools and methods described in Figure S8. Among all, 124 protein-coding alleles were predicted as « dubious » ORFs.

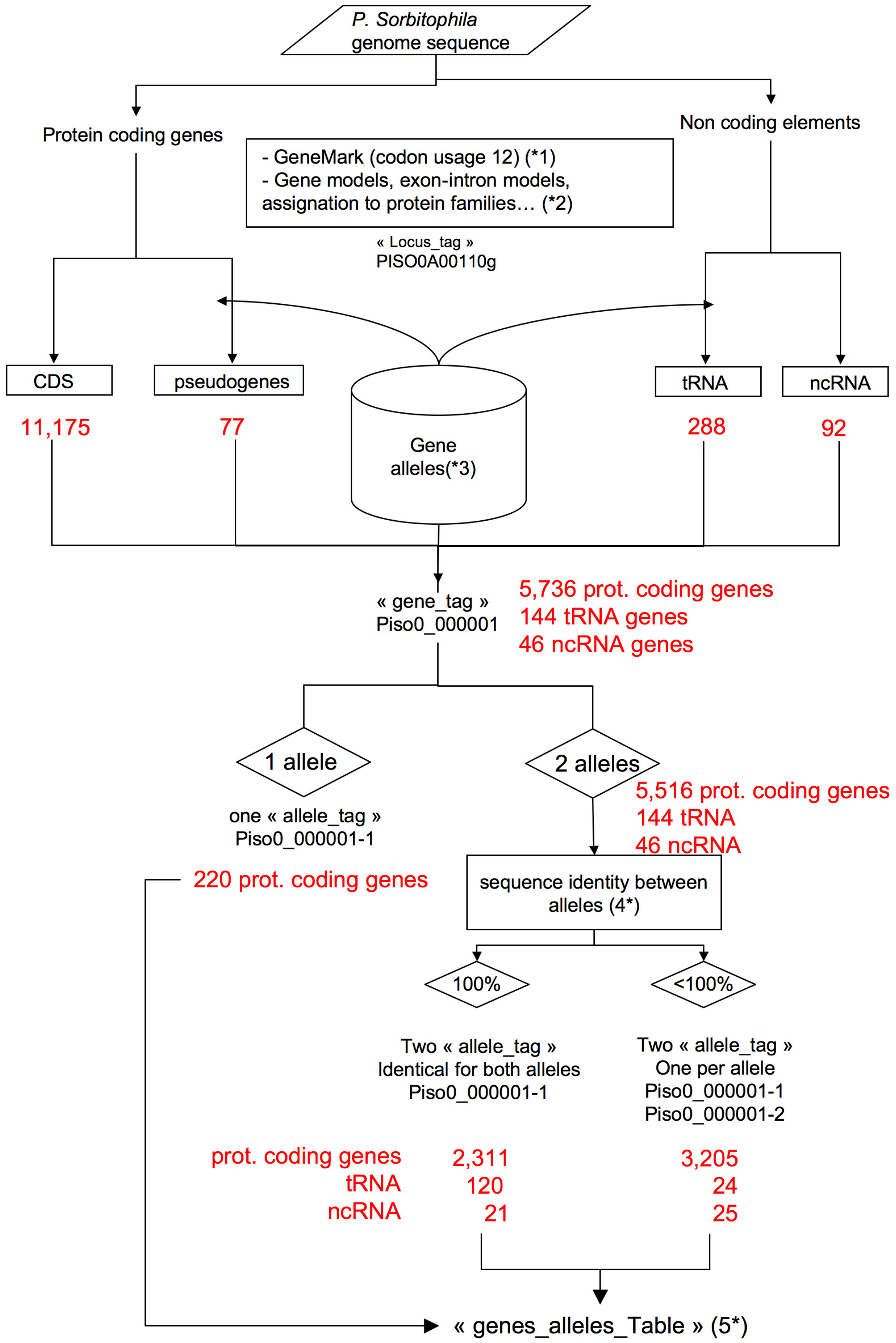
P. Sorbitophila
genome sequence
Protein coding genes
Non coding elements
- GeneMark (codon usage 12) (*1)
- Gene models, exon-intron models,
assignation to protein families… (*2)
« Locus_tag »
PISO0A00110g
CDS
pseudogenes
tRNA
ncRNA
11,175
77
288
92
Gene
alleles(*3)
« gene_tag »
Piso0_000001
5,736 prot. coding genes
144 tRNA genes
46 ncRNA genes
1 allele
2 alleles
one « allele_tag »
Piso0_000001-1
5,516 prot. coding genes
144 tRNA
46 ncRNA
220 prot. coding genes
sequence identity between
alleles (4*)
100%
<100%
Two « allele_tag »
Identical for both alleles
Piso0_000001-1
Two « allele_tag »
One per allele
Piso0_000001-1
Piso0_000001-2
prot. coding genes
tRNA
ncRNA
2,311
120
21
3,205
24
25
« genes_alleles_Table » (5*)

**Figure S6** Flowchart for the prediction and the annotation of each chromosomal feature in *P. sorbitophila* genome.
*1 Alternative Yeast Nuclear Code (http://www.ncbi.nlm.nih.gov/Taxonomy/Utils/wprintgc.cgi,transl_table=12)
*2 Gene detection and annotation according to the previously developed method (Souciet *et al*., 2009). Each feature is characterized by a unique “locus_tag” representing its chromosomal position, an example of “locus_tag” is given.
*3 For protein-coding genes, tRNA and ncRNA, annotations in (2*) were homogenized regarding to the gene synteny between chromosomes forming a pair. Each region of synteny loss was manually checked to assess the presence/absence of the considered gene/pseudogene. For each pair of chromosomes, we considered two syntenic genes/pseudogenes that share the same annotation as two variants of the same gene. They have the same “gene_tag” but can differed for the “allele_tag” (4*)
4* The two variants of a gene were compared at the nucleotide level along the whole length, using Needle from EMBOSS package (Rice *et al*., 2000). Same “alleles_tag” were attributed only in case of 100% identity.
*5 Relation between loci, gene and alleles are available in the “genes _alleles_table” at http://www.genolevures.org/
