# Supplementary material for: Pichia sorbitophila, an Interspecies Yeast Hybrid, Reveals Early Steps of Genome Resolution After Polyploidization
Source: G3 (Bethesda). 2012 Feb 1;2(2):299–311. doi: 10.1534/g3.111.000745 (PMC3284337; doi:10.1534/g3.111.000745)
Supplement: Supporting Information [file supp_2.2.299_FigureS7.pdf]

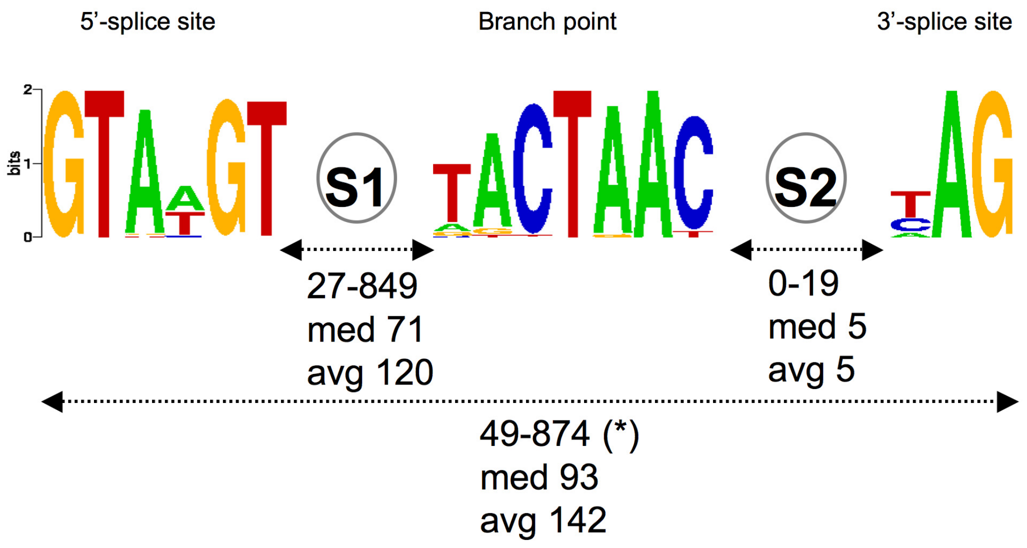

Figure S7 Splicing pattern determined for spliceosomal introns detected in *P. sorbitophila* genome. Minimal and maximal distances observed between exons (*), between the 5'-splice site and branch point (S1) and between the branch point and 3'-splice site (S2) are indicated, as well as the median (med) and the average (avg) values.
