# Supplementary material for: Pichia sorbitophila, an Interspecies Yeast Hybrid, Reveals Early Steps of Genome Resolution After Polyploidization
Source: G3 (Bethesda). 2012 Feb 1;2(2):299–311. doi: 10.1534/g3.111.000745 (PMC3284337; doi:10.1534/g3.111.000745)
Supplement: Supporting Information [file supp_2.2.299_FigureS8.pdf]

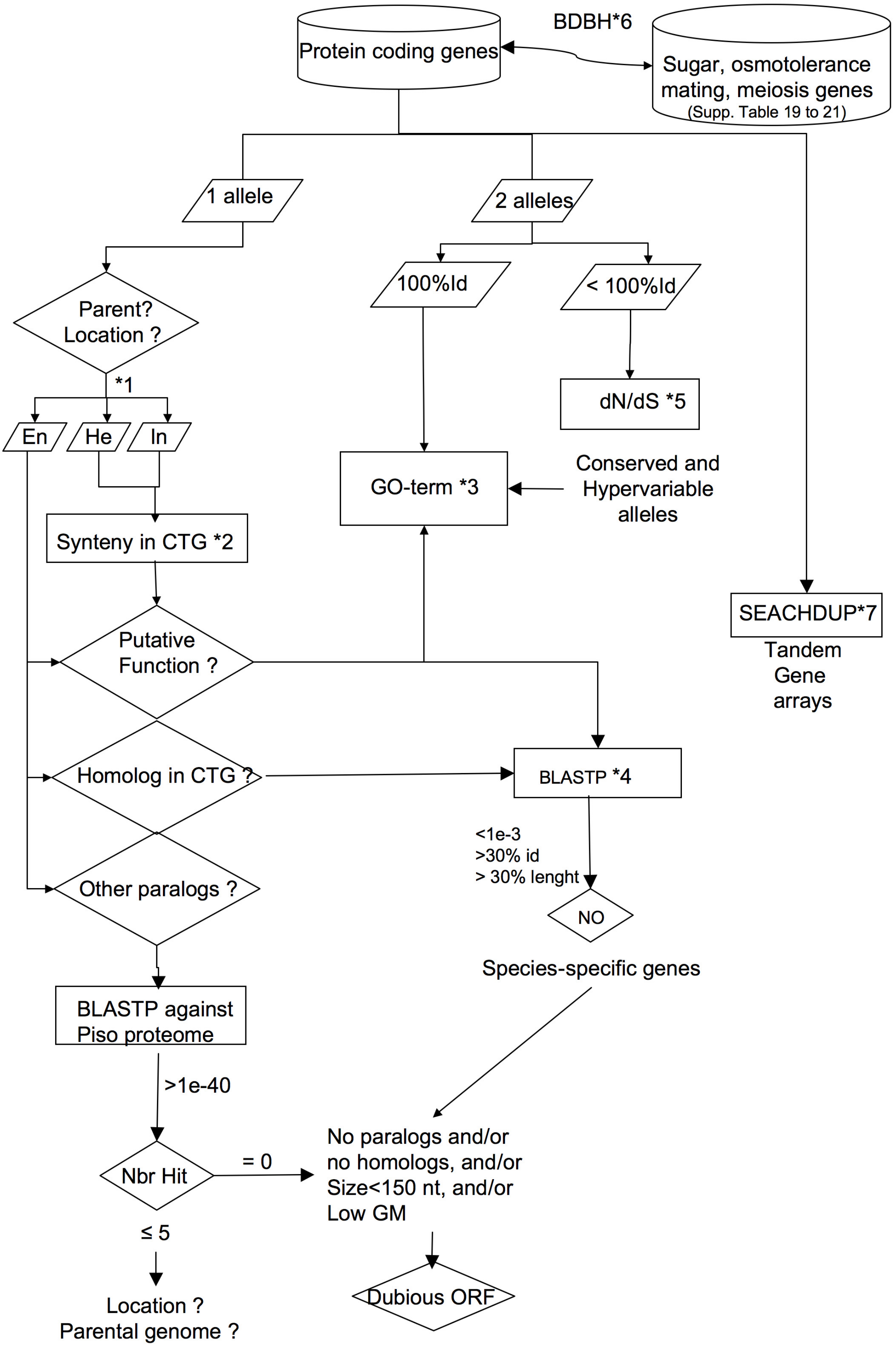
BDBH*6
Protein coding genes
Sugar, osmotolerance
mating, meiosis genes
(Supp. Table 19 to 21)
1 allele
2 alleles
Parent?
Location ?
*1
En
He
In
Synteny in CTG *2
Putative
Function ?
Homolog in CTG ?
Other paralogs ?
BLASTP against
Piso proteome
>1e-40
Nbr Hit
= 0
≤ 5
Location ?
Parental genome ?
100%Id
< 100%Id
dN/dS *5
GO-term *3
Conserved and
Hypervariable
alleles
BLASTP *4
<1e-3
>30% id
> 30% lenght
NO
Species-specific genes
No paralogs and/or
no homologs, and/or
Size<150 nt, and/or
Low GM
Dubious ORF
SEACHDUP*7
Tandem
Gene
arrays

Figure S8 Flowchart for gene analyses and comparisons.
*1 « he », « en » and « in » refer to **he**terozygous regions, **en**d of contig and E/F/I/J translocation breakpo**in**t, respectively. Location and parental subgenome attribution were determined using the table of genes and alleles.
*2 Synteny was analysed first by searching orthologs of neighbor genes in *D. hansenii* using protein families (Sherman *et al*., 2009) and the genome browser available for this species at http://www.genolevures.org/ (Souciet *et al*., 2009). Gene orders in *C. guilliermondii, P. stipitis* and *C. albicans* were obtained from the "Yeast Gene Order Browser" web site http://wolfe.gen.tcd.ie/ygob (Byrne and Wolfe, 2006) using *D. hansenii* genes as queries.
*3 GO-Slim terms were associated to *P. sorbitophila* genes on the basis of orthology with *S. cerevisiae* gene products, considering that all GO-Slim terms of a *S. cerevisiae* gene are transferable to its ortholog. In order to define the functions over or underrepresented in a given group of genes, GO-Slim frequencies for them and for all other genes were calculated and statistically compared.
*4 Species-specific genes were extracted from the whole set of *P. sorbitophila* genes using BLASTP (Altschul *et al*., 1990) against *P. stipitis, C. guilliermondii, C. lusitaniae, C. albicans*, *C. dubliniensis*, *C. tropicalis*, *C. parapsilosis*, *L. elongisporus* and *D. hansenii* proteomes with 1.e-3 as threshold for the expect value and 30% of identity for at least 30% of the query sequence as the threshold for the length of the alignment.
*5 The ratio of non-synonymous substitutions (dN) per synonymous substitutions (dS) was calculated for each gene located in heterozygous regions and having two coding alleles by the following procedure**:** protein sequences for each allelic pair were aligned using *ClustalW2* (Thompson *et al*., 2002). The nucleotide sequences were aligned with *tranalign* from the *EMBOSS* package (Rice *et al*., 2000) using the corresponding set of aligned protein sequences for each allele pair to obtain the nucleic acid sequence translation of the protein alignment, and using the alternative yeast nuclear genetic code (transl_table=12). The dN/dS ratio for the obtained alignment was performed using the *yn00* program from the *PAML package* (Yang, 2007) and calculated by the method of Yang and Nielsen (2000)
*6 Homologs in *P. sorbitophila* were identified by reciprocal blast searches according to the method described in Mushegian and Koonin (1996). Alignments for genes involved in sugar degradation and osmotolerance were manually curated.
*7 Tandem gene arrays were detected by analysing similarities between neighbor genes using the previously developed method described in Despons *et al*. (2010).
