# Supplementary material for: Pichia sorbitophila, an Interspecies Yeast Hybrid, Reveals Early Steps of Genome Resolution After Polyploidization
Source: G3 (Bethesda). 2012 Feb 1;2(2):299–311. doi: 10.1534/g3.111.000745 (PMC3284337; doi:10.1534/g3.111.000745)
Supplement: Supporting Information [file supp_2.2.299_FigureS9.pdf]

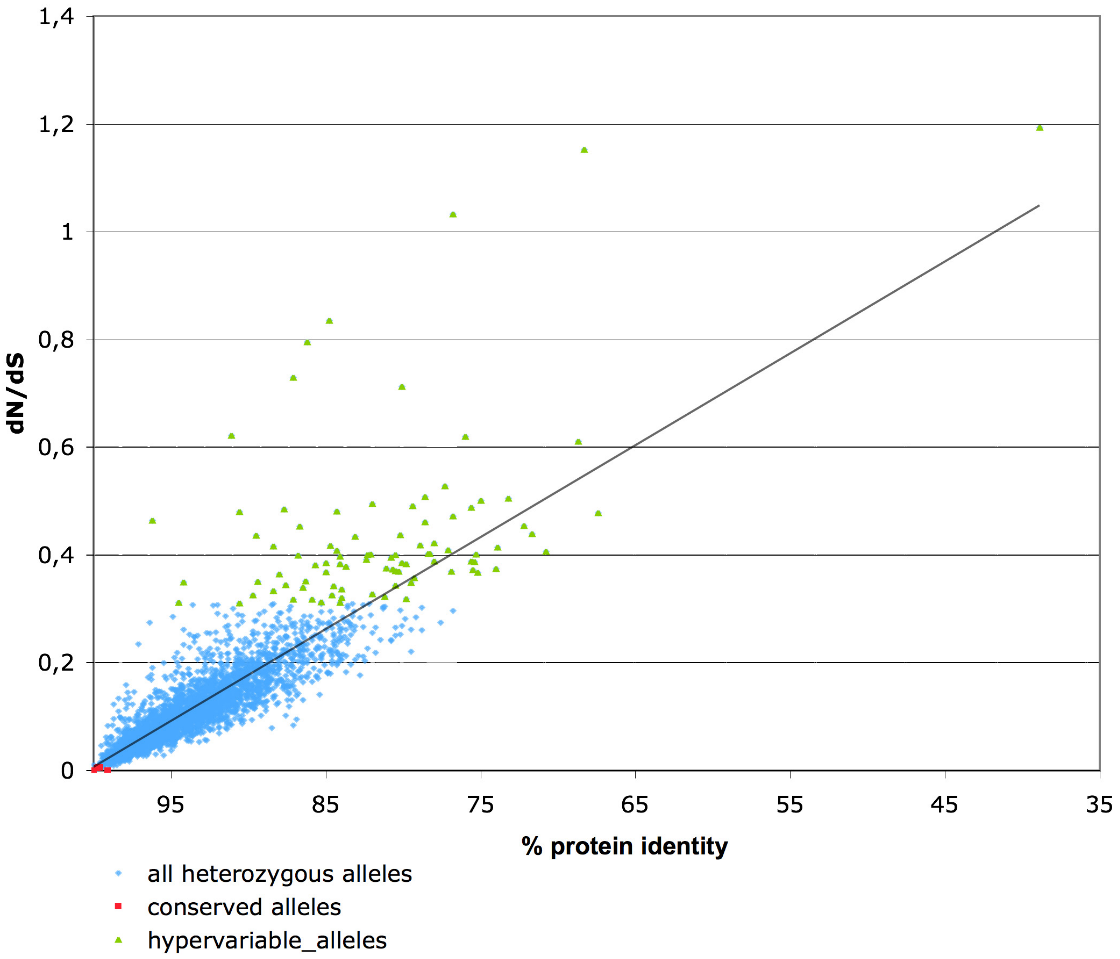


**Figure S9** Distribution of dN/dS values according to the protein sequence identity. The dN/dS ration (Y-axe) was calculated for 3,111 allele pairs aligned without conflicts (see Supp Fig. 8 for method). Protein identity (X-axe) was calculated for the aligned part of the sequences. Best fit line is also indicated ($R^2$= 0.7311). dN/dS mean value = 0.121, median= 0.102 [0.063-0.156]. Three genes show a dN/dS value > to 1 but present also CDS size of 225 to 339 nt and have no other homologs. They likely correspond to dubious ORFs.
