# Supplementary material for: Pichia sorbitophila, an Interspecies Yeast Hybrid, Reveals Early Steps of Genome Resolution After Polyploidization
Source: G3 (Bethesda). 2012 Feb 1;2(2):299–311. doi: 10.1534/g3.111.000745 (PMC3284337; doi:10.1534/g3.111.000745)
Supplement: Supporting Information [file supp_2.2.299_FigureS10.pdf]

3. Ribosomal DNA

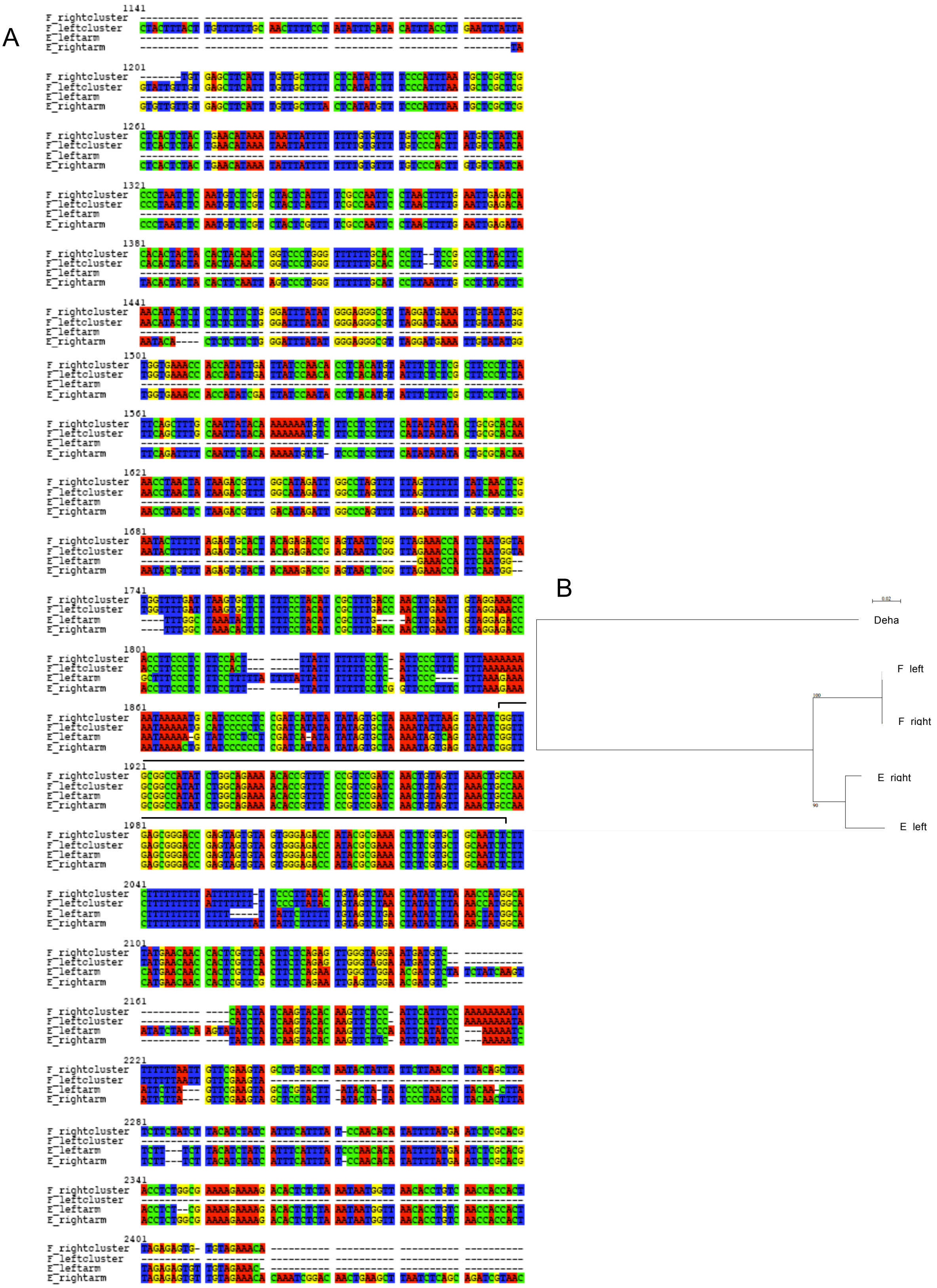

Figure S10 Comparison of 5S rDNA sequences. (A) Multiple alignment of the rDNA loci performed with Muscle software (Edgard, 2004) and visualized with Jalview (Waterhouse *et al*., 2009). E_leftarm and E_rightarm correspond to both rDNA loci on chr. E, F_leftcluster and F_rightcluster correspond to the 5S sequences flanking the 73 tandem repeats (Fig. 4). (B)

Phylogenetic tree calculated using the Bio-NJ distance method in Seaview (Gouy *et al.*, 2010) for 100 replicata and D. hansenii as outgroup.
