# Supplementary material for: Pichia sorbitophila, an Interspecies Yeast Hybrid, Reveals Early Steps of Genome Resolution After Polyploidization
Source: G3 (Bethesda). 2012 Feb 1;2(2):299–311. doi: 10.1534/g3.111.000745 (PMC3284337; doi:10.1534/g3.111.000745)
Supplement: Supporting Information [file supp_2.2.299_FigureS12.pdf]

4. Mitochondrial genome

A recent phylogenetic analysis of the CTG yeast mitochondrial genomes (mt) places the *P. sorbitophila* mt genome at the base of this group: *P. farinosa* (CBS 185), *P. stipitis*, *D. hansenii* and *P. guillermondii* mt genomes are more closely related to each other than to *P. sorbitophila* (Jung *et al*., 2010). Two facts suggest that the *P. sorbitophila* mt genome is inherited from the Pε parent: i) its mt genome is very distant from others *Pichia* species, and ii) *P. sorbitophila* mt *COX2* gene sequence diverges from the one sequenced in *P. farinosa* CBS 2001 (Mallet *et al*., in preparation), a strain very closely related to Pγ (Table S4).

5. tRNA and co-transcribed tRNA, tRNA gene usage

The *P. sorbitophila* genome contains a total of 144 tRNA genes (Table S14), all of them being represented by two alleles, 88 in heterozygous and 56 in homozygous regions. This value is significantly lower than for *D. hansenii* with 200 tRNA genes (Table S14). The 88 tRNA genes located in heterozygous regions show a total of 18 SNP between alleles (outside intronic and extra arm sequences), corresponding to less than 0.3% of sequence divergence. Groups of two neighbouring and co-oriented tRNA genes are present in one or four copies in the genome (Table S15). The neighbouring tRNAs genes are separated by only eight to 13 nucleotides suggesting that they are co-transcribed (Acker *et al*., 2008).

The codon and tRNA gene usages were also compared with *D. hansenii* (Table S16). In *P. sorbitophila*, as in the other yeasts of the CTG group (Santos *et al*., 1996, Perreau *et al*., 1999, Marck *et al*., 2006), the decoding properties of both tRNA-Leu (AAG, reading CTT, CTC and CTA codons) and tRNA-Ser (CAG, reading the usual CTG codon as Ser) are finely tuned by two unusual G nucleotides located 5' of the anticodon (G32 and G33, respectively, instead of the predominant C32 and U33).

6. Other noncoding RNA genes

We were able to identify 46 ncRNA genes (Supp. Table 17): the small nuclear RNAs (U1, U2, U4, U5 and U6); the RNA components of the RNase P; the signal recognition particle; 17 H/ACA and 35 C/D snoRNAs. All these ncRNA are evenly distributed over the 7 pairs of chromosomes including 17 in the homozygous chromosomal regions (essentially snoRNAs). The remaining 29 ncRNAs (found in heterozygous regions) are all represented by two highly similar alleles sharing on average 96.0 % of nucleotide identity. Compared to the average protein-coding genes identity (90.9 % in the coding sequence), ncRNA genes are therefore highly conserved.

7. Synteny breaks between Pγ and Pε subgenomes

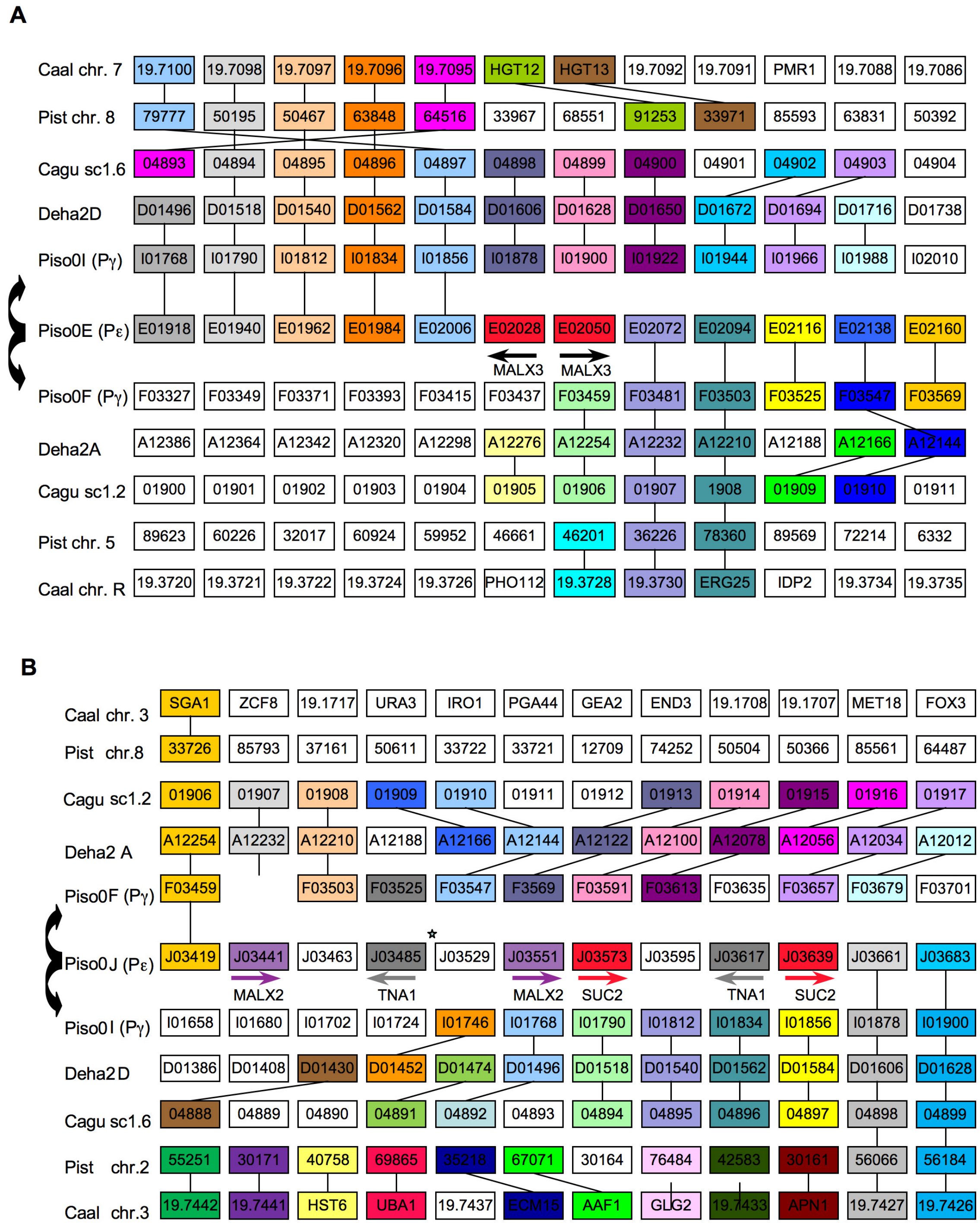

Figure S12 Comparison of synteny maps at the E/F/I/J reciprocal translocation between CTG yeasts. (A) Gene order on chr. E showing a synteny shift from chr. I to chr. F. (B) Gene order on chr. J showing a synteny shift from chr. F to chr. I. Tandemly duplicated genes and pseudogenes are indicated by arrows and star, respectively. Orthologs are represented by same color and line-connected.
