# Supplementary material for: Pichia sorbitophila, an Interspecies Yeast Hybrid, Reveals Early Steps of Genome Resolution After Polyploidization
Source: G3 (Bethesda). 2012 Feb 1;2(2):299–311. doi: 10.1534/g3.111.000745 (PMC3284337; doi:10.1534/g3.111.000745)
Supplement: Supporting Information [file supp_2.2.299_FigureS11.pdf]

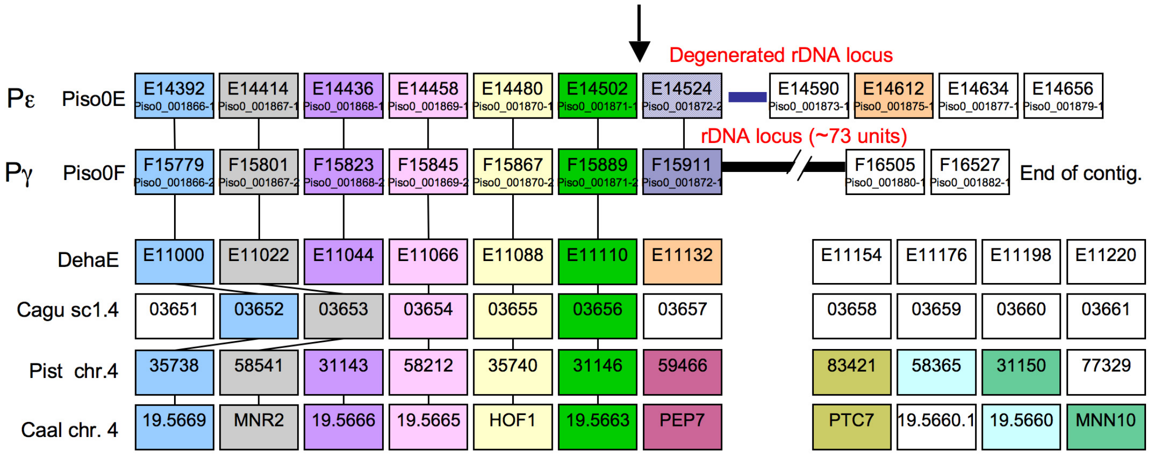

Figure S11 Synteny conservation around the rDNA clusters located on the left arm of *P. sorbitophila* chr E and F. Within the five CTG species, orthologous genes (or alleles for *P. sorbitophila*) are represented by same colors and line connected. The locus name is indicated for each species according to the published nomenclature. For *P. sorbitophila*, both loci and alleles are indicated (see also Figure S8). Hatched box corresponds to pseudogene. Synteny breakpoint is represented by an arrow.
