# Supplementary material for: Pichia sorbitophila, an Interspecies Yeast Hybrid, Reveals Early Steps of Genome Resolution After Polyploidization
Source: G3 (Bethesda). 2012 Feb 1;2(2):299–311. doi: 10.1534/g3.111.000745 (PMC3284337; doi:10.1534/g3.111.000745)
Supplement: Supporting Information [file supp_2.2.299_FigureS13.pdf]

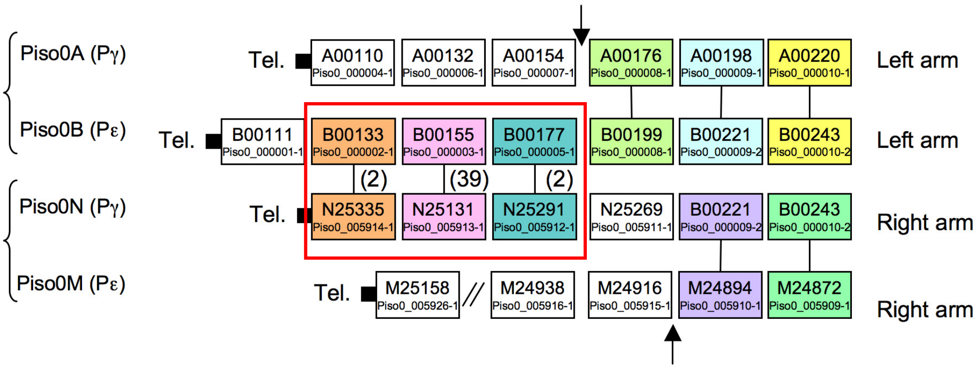

Figure S13 Gene location movement between two subtelomeric regions. Alleles are represented by connected boxes of same color. The comparison between subtelomeric regions of A/B and M/N chromosomal pairs shows breaks of synteny (arrows). The last three genes (toward the telomere) on chr. N (red box) have no equivalent allelic position on chr M. The second copy of these genes is found in the subtelomeric region of chr B, in same orientation. The number of paralogs identified for each gene is indicated in brackets. Among the three genes, two are represented by these only two copies in the genome of *P. sorbitophila*.
