# Supplementary material for: Pichia sorbitophila, an Interspecies Yeast Hybrid, Reveals Early Steps of Genome Resolution After Polyploidization
Source: G3 (Bethesda). 2012 Feb 1;2(2):299–311. doi: 10.1534/g3.111.000745 (PMC3284337; doi:10.1534/g3.111.000745)
Supplement: Supporting Information [file supp_2.2.299_FigureS14.pdf]

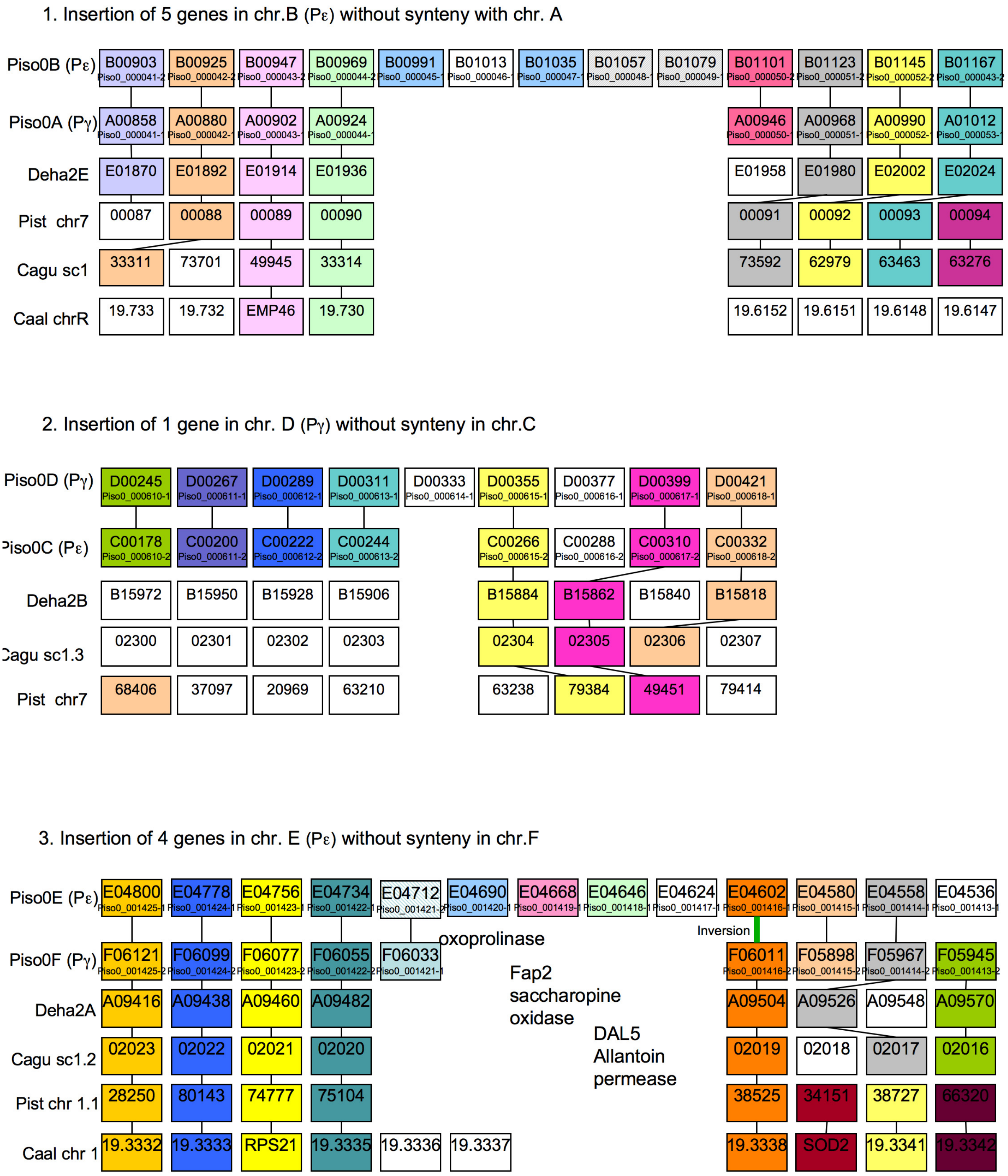

Figure S14 Comparison of synteny maps at single allele gene positions between CTG yeasts (seven studied cases). Alleles of genes (*P. sorbitophila*) and orthologs are represented by same color and line-connected. Hatched box corresponds to a pseudogene. One case of inversion is indicated by a green connector between alleles.

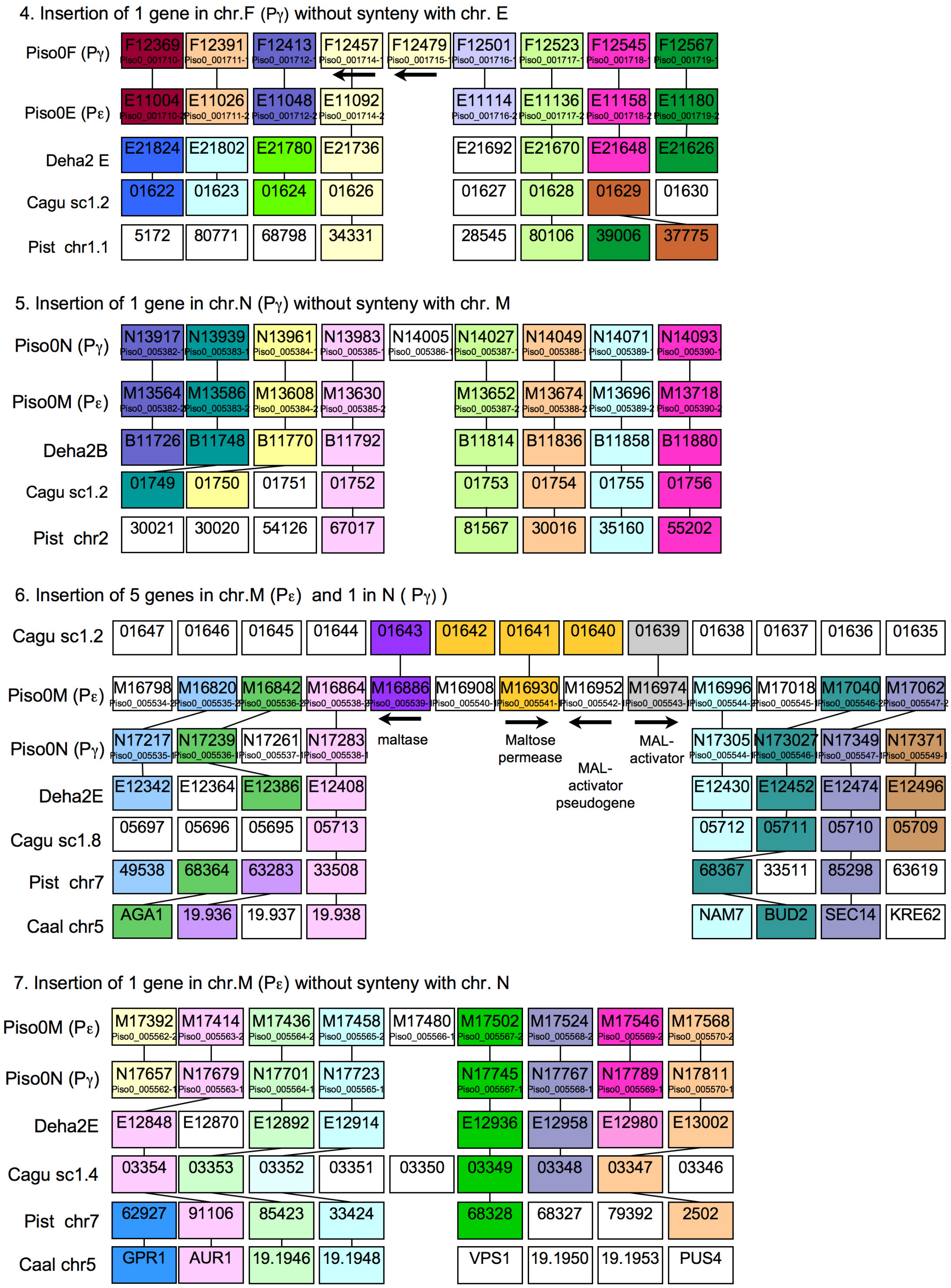

Figure S14 Comparison of synteny maps at single allele gene positions between CTG yeasts (seven studied cases), cases four to seven.
