# Supplementary material for: Pichia sorbitophila, an Interspecies Yeast Hybrid, Reveals Early Steps of Genome Resolution After Polyploidization
Source: G3 (Bethesda). 2012 Feb 1;2(2):299–311. doi: 10.1534/g3.111.000745 (PMC3284337; doi:10.1534/g3.111.000745)
Supplement: Supporting Information [file supp_2.2.299_FigureS15.pdf]

8. Mating type

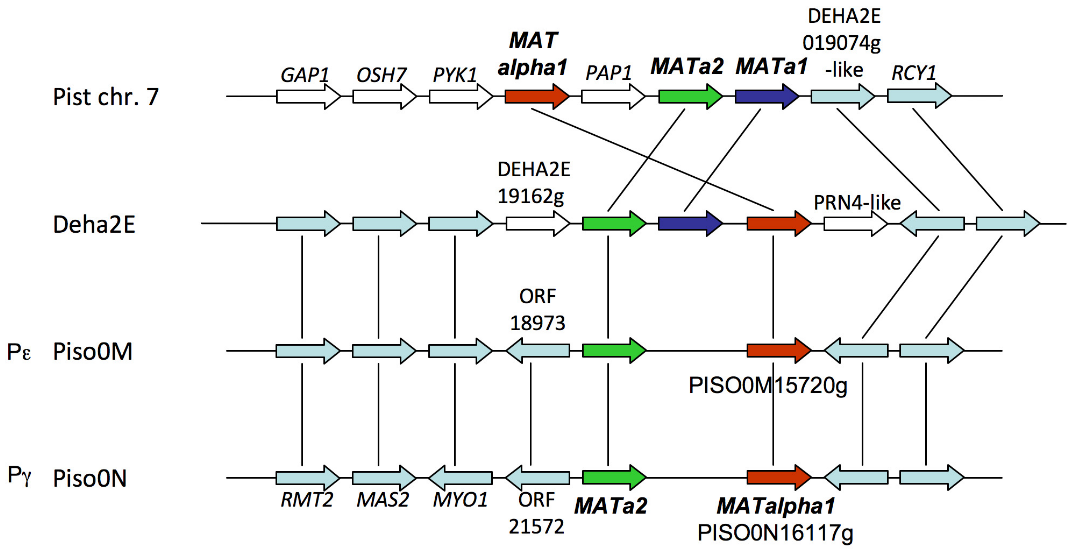

Figure S15 Comparative organization of the mating type locus (MTL) in *P. sorbitophila*, *D. hansenii* and *P. stipitis*. Conserved MTL genes are coloured: alpha idiomorph, in red; a1, in blue; a2, in green. Orthologous genes line connected. In *P. stipitis, MATa1* is annotated as *YOX2* and *MATalpha1* is not annotated.
