# Supplementary material for: Pichia sorbitophila, an Interspecies Yeast Hybrid, Reveals Early Steps of Genome Resolution After Polyploidization
Source: G3 (Bethesda). 2012 Feb 1;2(2):299–311. doi: 10.1534/g3.111.000745 (PMC3284337; doi:10.1534/g3.111.000745)
Supplement: Supporting Information [file supp_2.2.299_TableS1.pdf]

Table S1 Heterozygous and homozygous parts of *P. sorbitophila* genome

chr. Pair*	chr.	Chr. Size (nt)	Size of homozygous region (nt)	Size of heterozygous region** (nt)	Homozygous Part (%)	Heterozygous Part †(%)	Size of hetero. reg. w/o ins/del√ (nt)	% seq. identity in hetero. regions†	% seq. identity in hetero. regions w/o ins/del¥	Telomeric repeats &
A/B	Chr. A	1,055,225	498,502	556,723	47.2	52.8	551,536	87.45	89.74	L and R
	Chr. B	1,063,002	498,502	564,500	46.9	53.1	550,398			R
C/D	Chr. C	1,095,816	467,624	628,192	42.7	57.3	622,613	87.18	88.75	R
	Chr. D	1,102,865	467,624	635,241	42.4	57.6	622,743			R
E/I	Chr. E	1,307,235	0	178,135	0	100	149,929	70.61	87.10	-
	Chr. I	1,666,063	0	162,406	0	100	151,514			-
E/F	Chr. E	1,307,235	0	1,129,100	0	100	1,068,026	85.07	89.47	L and R
	Chr. F	1,407,903	0	1,119,295	0	100	1,071,047			L
F/J	Chr. F	1,407,903	0	288,608	0	100	284,415	88.33	89.31	-
	Chr. J	1,803,284	0	285,346	0	100	283,178			-
I/J	Chr. I	1,666,063	0	1,503,657	0	100	1,202,358	88.63	89.14	R
	Chr. J	1,803,284	0	1,517,939	0	100	1,196,297			R
G/H	Chr. G	1,423,303	1,423,303	0	100	0	-	-	-	L and R
	Chr. H	1,423,303	1,423,303	0	100	0	-			L and R
K/L	Chr. K	1,933,849	1,933,849	0	100	0	-	-	-	L
	Chr. L	1,933,849	1,933,849	0	100	0	-			L
M/N	Chr. M	2,121,241	0	2,121,241	0	100	2,048,627	85.64	89.10	L and R
	Chr. N	2,122,704	0	2,122,704	0	100	2,049,178			L
**Total genome**		**21,459,642**	**8,646,556**	**12,813,086**	**40.3**	**59.7**	**11,851,959**	**84.70**	**89.16**	-

* according to identity (Figure S3). ** Size of the whole sequence of each heterozygous region, from telomeric repeats until the last SNP identified. † Calculated using the complete heterozygous regions **. √ Size of the heterozygous regions excluding areas of identity drop, i.e telomeric and subtelomeric non aligned sequences and internal insertions/deletions. ¥ percentage of identity calculated on the basis of the global alignment of regions presenting no drop of identity (√). Sequences were aligned using Stretcher from EMBOSS (Rice et al., 2000). The total percentage of identity is the sum of all identity in heterozygous regions divided by the sum of all aligned positions. & Telomeres were detected at the ends of contigs by searching repeats rich in C/A (inward orientation) and in G/T (outward orientation). They were found at both ends for chr. A, E, G, H and M, at the right end for chr. B, C, D, I and J, and at the left end for chr. F, K, L and N. The motif CCCAAACA is repeated without variation.
