# Supplementary material for: Pichia sorbitophila, an Interspecies Yeast Hybrid, Reveals Early Steps of Genome Resolution After Polyploidization
Source: G3 (Bethesda). 2012 Feb 1;2(2):299–311. doi: 10.1534/g3.111.000745 (PMC3284337; doi:10.1534/g3.111.000745)
Supplement: Supporting Information [file supp_2.2.299_TableS2.pdf]

Table S2 Sequence polymorphism in homozygous regions of the *P. sorbitophila* genome

chr. pair	size_region of homozygosity per chr.	size of covered area in region of homozygosity	% of coverage in region of homozygosity	Nbr. SNP in covered area	Nbre of Indel in covered area	Average SNP/indel density
A/B	498,502	393,074	78.85	22	0	1/35,734
C/D	467,624	411,796	88.06	15	1	1/51,474
G/H	1,423,303	1,421,984	99.90	158	0	1/18,000
K/L	1,933,849	1,933,590	99.98	186	1	1/20,680

The SNP detection procedure is based on the mapping of the trimmed reads on the genome reference sequence with SSAHA2 (Ning *et al*., 2001). The variant positions were filtered according to the identified bases quality (Q>25), the number of reads containing the SNP and the local coverage.
