# Supplementary material for: Pichia sorbitophila, an Interspecies Yeast Hybrid, Reveals Early Steps of Genome Resolution After Polyploidization
Source: G3 (Bethesda). 2012 Feb 1;2(2):299–311. doi: 10.1534/g3.111.000745 (PMC3284337; doi:10.1534/g3.111.000745)
Supplement: Supporting Information [file supp_2.2.299_TableS3.pdf]

Table S3 NUMTs in the nuclear genome of *Pichia sorbitophila*

Nb	Mitochondrial coordinates	Mitochondrial gene/intergene	orienta-tion	size (nt)	Percentage identity*	Chr.	Chromosomal coordinates	Parental subgenome	NUMT organization	Cluster type	Allele organization
p1	20708-20744	cox1 CDS ai5 ai4	>	37	97	Piso0A	984134-984170	Pe	single		homozygous (with NUMT p5)
p2	38123-38152	Nad2	>	30	96	Piso0A	1035013-1035042	Pe	cluster 1	mosaic	homozygous (with mosaic 2)
p3	19871-19922	cox1 CDS ai5 ai4	>	52	94	Piso0A	1035162-1035213	Pe	cluster 1	mosaic	
p4	19977-20063	cox1 CDS ai5 ai4	>	87	83	Piso0A	1035220-1035306	Pe	cluster 1	mosaic	
p5	20708-20744	cox1 CDS ai5 ai4	>	37	97	Piso0B	991911-991947	Pe	single		homozygous (with NUMT p1)
p6	38123-38152	Nad2	>	30	96	Piso0B	1042790-1042819	Pe	cluster 2	mosaic	homozygous (with mosaic 1)
p7	19871-19922	cox1 CDS ai5 ai4	>	52	94	Piso0B	1042939-1042990	Pe	cluster 2	mosaic	
p8	19977-20063	cox1 CDS ai5 ai4	>	87	83	Piso0B	1042997-1043083	Pe	cluster 2	mosaic	
p9	35906-35958	tRNA-Glu	<	53	100	Piso0E	81462-81514	Pe	single		hemizygous (absent on Piso0I)
p10	27200-27253	Cox1	>	53	90	Piso0E	310115-310167	Pe	cluster 3	mosaic	hemizygous (absent on Piso0F)
p11	33141-33180	Nad1	>	40	90	Piso0E	310167-310206	Pe	cluster 3	mosaic	
p12	36888-36929	Atp6	>	42	95	Piso0E	629315-629356	Pe	single		hemizygous (absent on Piso0F)
p13	14902-15036	Nad5	<	135	92	Piso0E	673200-673334	Pe	single		hemizygous (absent on Piso0F)
p14	21204-21227	cox1 CDS ai5 ai4	<	24	95	Piso0G	481070-481093	Pg	single		homozygous (with NUMT p15)
p15	21204-21227	cox1 CDS ai5 ai4	<	24	95	Piso0H	481070-481093	Pg	single		homozygous(withNUMT p14)
p16	33508-33552	Nad1	<	45	91	Piso0I	9413-9457	Pg	cluster	procession	hemizygous (absent on Piso0E)
p17	33267-33492	Nad1	<	224	86	Piso0I	9448-9671	Pg	cluster	procession	
p18	3957-3993	cob bi4 bi3 bi2	>	37	97	Piso0J	783025-783061	Pe	single		hemizygous (absent on Piso0I)
p19	34992-35101	intergene	>	110	98	Piso0J	1034596-1034705	Pe	single		hemizygous (absent on Piso0I)
p20	37866-37916	Nad2	>	51	90	Piso0J	1330982-1331032	Pe	cluster 4	mosaic	hemizygous (absent on Piso0I)
p21	17989-18033	cox1 CDS ai5 ai4 ai3 ai2	>	45	97	Piso0J	1331053-1331097	Pe	cluster 4	mosaic	
p22	20709-20743	cox1 CDS ai5 ai4	<	35	97	Piso0J	1331102-1331136	Pe	cluster 4	mosaic	
p23	10164-10195	tRNA-Phe	>	32	96	Piso0J	1331137-1331168	Pe	cluster 4	mosaic	
p24	28250-28291	Cox1	<	43	93	Piso0M	158431-158473	Pe	single		hemizygous (absent on Piso0N)

NUMTs (NUclear sequences of MiTochondrial origin) in *P. sorbitophila* genome were identified on the basis of the complete published sequence (39,107 bp) of the mitochondrial genome (Jung *et al*., 2010), using BLASTN searches (Altschul *et al*., 1990) and according to the previously developed method described by Sacerdot *et al (2008).* To test the hypothesis that some NUMTs present in the Pγ subgenome were not identified due to poor sequence identities with the *P. sorbitophila* mitochondrial genome, we re-iterated the procedure by using the mitochondrial genome of *P. farinosa* CBS 185, but without obtaining additional NUMTs insertion loci in Pγ. * percentage of identity between NUMTs and mtDNA
