# Supplementary material for: Pichia sorbitophila, an Interspecies Yeast Hybrid, Reveals Early Steps of Genome Resolution After Polyploidization
Source: G3 (Bethesda). 2012 Feb 1;2(2):299–311. doi: 10.1534/g3.111.000745 (PMC3284337; doi:10.1534/g3.111.000745)
Supplement: Supporting Information [file supp_2.2.299_TableS4.pdf]

Table S4 Sequence identity between *P. farinosa CBS 2001* and *P. sorbitophila* subgenomes

region	feature	chr_1 *	position_1 $	locus_1	Identity_1 to P. farinosa CBS2001 &	subgenome attribution for locus _1 £	chr_2*	position_2 $	locus_2	Identity_2 to P. farinosa CBS2001 &	subgenome attribution for locus _2 £	accession number
heterozygous	gene	B	176663..177097	PISO0B02069g	93	Pε	A	168748..169182	PISO0A01892g	100	Pγ	FN994818
homozygous	gene	B	796873..797371	PISO0B09835g	94	Pε	A	789096..789594	PISO0A09768g	94	Pε	FN994814
homozygous	NUMT p1	B	992082..991685	PISO0B12453g-PISO0B12475g	73 (no NUMT)	Pε	A	983908..984305	PISO0A12386g-PISO0A12408g	73 (no NUMT)	Pε	JQ267632
heterozygous	gene	C	151119..151546	PISO0C01806g	92	Pε	D	158180..158610	PISO0D01895g	100	Pγ	FN994825
heterozygous	gene : Pγ-Pε exchange	C	383527..384113	PISO0C04512g	98-99	Pγ	D	391163..391749	PISO0D04623g	99-97	Pγ	JQ267644
homozygous	gene	C	868158..868568	PISO0C10474g	100	Pγ	D	875207..875617	PISO0D10541g	100	Pγ	FN994821
heterozygous	gene	E	99549..100005	PISO0E01214g	92	Pε	I	84142..84602	PISO0I01042g	100	Pγ	FN994836
heterozygous	gene	E	254393..254869	PISO0E02930g	93	Pε	F	361850..362326	PISO0F04339g	99	Pγ	FN994833
heterozygous	gene	E	1185601..1186066	PISO0E13534g	95	Pε	F	1289721..1290186	PISO0F14921g	100	Pγ	FN994829
heterozygous	gene	J	208409..208838	PISO0J02473g	93	Pε	F	211321..211750	PISO0F02513g	100	Pγ	FN994840
heterozygous	last gene in F/J syneny	J	283861..284508	PISO0J03419g	90	Pε	F	287111..287770	PISO0F03459g	100	Pγ	JQ267642
heterozygous	end of F/J syneny	J	285133..285342	PISO0J03419g	85	Pε	F	288395..288881		100	Pγ	JQ267639
heterozygous	begining of F/E synteny	E	181320..181550	PISO0E02050g-PISO0E02072g	91	Pε			PISO0F03459g-PISO0F03481g		Pγ	
heterozygous	last gene in I/E syneny	E	177149..177618	PISO0E02006g	91	Pε	I	161728..162198	PISO0I01856g	100	Pγ	JQ267640
heterozygous	end of I/E syneny	E	177685..177840	PISO0E02006g-PISO0E02028g	93	Pε	I	162265..162748		99	Pγ	JQ267643
heterozygous	begining of I/J synteny	J	305395..305708	PISO0J03661g	94	Pε			PISO0I01856g-PISO0I01878g		Pγ	
heterozygous	gene	J	516929..517387	PISO0J06081g	91	Pε	I	375063..375521	PISO0I04276g	100	Pγ	FN994853
heterozygous	gene	J	1521249..11521709	PISO0J18269g	93	Pε	I	1384553..1385013	PISO0I16508g	100	Pγ	FN994849
homozygous	gene	G	172080..172490	PISO0G02294g	100	Pγ	H	172080..172490	PISO0H02295g	100	Pγ	FN994846
homozygous	gene	G	301055..301682	PISO0G03856g	100	Pγ	H	301055..301682	PISO0H03857g	100	Pγ	JQ267635

homozygous	gene	G	301813..302440	PISO0G03856g	100	Pγ	H	301813..302440	PISO0H03857g	100	Pγ	JQ267641
homozygous	NUMTs p14-15	G	480960..481261	PISO0G06122g-PISO0G06144g	100	Pγ	H	480960..481261	PISO0H06123g-PISO0H06145g	100	Pγ	JQ267633
homozygous	Intergene	G	1083262..1084635	PISO0G13734g-PISO0G13712g	99	Pγ	H	1083262..1084635	PISO0H13735g-PISO0H13713g	99	Pγ	JQ267636
homozygous	gene	G	1236388..1236848	PISO0G15538g	100	Pγ	H	1236388..1236848	PISO0H15539g	100	Pγ	FN994843
homozygous	intergene	K	100922..101449	PISO0K01286g-PISO0K01264g	99	Pγ	L	100922..101449	PISO0L01287g-PISO0L01265g	99	Pγ	JQ267637
homozygous	gene	K	234563..234998	PISO0K02738g	100	Pγ	L	234563..234998	PISO0L02739g	100	Pγ	FN994860
homozygous	intergene	K	1063363..1063459	PISO0K12528g-PISO0K12506g	100	Pγ	L	1063363..1063459	PISO0L12529g-PISO0L12507g	100	Pγ	**
homozygous	intergene	K	1063482..1063698	PISO0K12528g-PISO0K12550g	100	Pγ	L	1063482..1063698	PISO0L12529g-PISO0L12551g	100	Pγ	JQ267645
homozygous	gene	K	1749204..1749606	PISO0K21350g	100	Pγ	L	1749204..1749606	PISO0L21351g	100	Pγ	FN556130
homozygous	intergene	K	1462003..1462275	PISO0K17830g-PISO0K17808g	100	Pγ	L	1462003..1462275	PISO0L17831g-PISO0L17809g	100	Pγ	JQ267638
heterozygous	gene	M	169354..169782	PISO0M02058g	96	Pε	N	188531..188959	PISO0N02191g	99	Pγ	FN994867
heterozygous	NUMT p24	M	158164..158608	PISO0M01926g-PISO0M01924g	75	Pε	N	177405..177814	PISO0N02059g-PISO0N02037g	100	Pγ	JQ267634
heterozygous	gene	M	1827135..1827615	PISO0M21550g	95	Pε	N	1837559..1838039	PISO0N21793g	100	Pγ	FN994863

The table indicates the position of selected markers in *P. sorbitophila* genome used in Mallet *et al*. (in preparation) for a taxonomical study of *P. farinosa* species (in blue) and used in these study (in black). These markers are present in two allelic versions (_1 and _2) either at a heterozygous or at a homozygous state (column "region").They correspond to part of genes, intergenes or NUMTs (column "feature"). Position of each allele is indicated(*). These markers were used for PCR amplifications and sequencing in *P. farninosa* CBS 2001 using oligonucleotides that hybridized on both alleles. Identity between the obtained *P. farinosa* sequences and each allele of *P. sorbitophila* are indicated ($). According to the sequence identities shared between *P. farinosa* and *P. sorbitophila* alleles, the belonging of each allele into one parental subgenome Pγ or Pε is proposed (£). These markers are plotted on Figure 1

**corresponding sequence : GATAATTATGATGTGTAAATCGAACTTGGAAATTCAACAAATACTGCAATTTTTTTCTACTCTTTTTTTCCAACTAGGACACTTCTTGGGCACAATG
