# Supplementary material for: Pichia sorbitophila, an Interspecies Yeast Hybrid, Reveals Early Steps of Genome Resolution After Polyploidization
Source: G3 (Bethesda). 2012 Feb 1;2(2):299–311. doi: 10.1534/g3.111.000745 (PMC3284337; doi:10.1534/g3.111.000745)
Supplement: Supporting Information [file supp_2.2.299_TableS5.pdf]

**Table S5 Databases for yeast species used in this study**

| species | strain | web site | Reference |
|---|---|---|---|
| *Debaryomyces hansenii* | CBS767 | http://www.genolevures.org/deha.html# | The Génolevures Consortium, 2009 (Souciet *et al.*) |
| *Yarrowia lipolytica* | E150 | http://www.genolevures.org/yali.html# | The Génolevures Consortium, 2009 (Souciet *et al.*) |
| *Candida albicans* | SC5314 | http://www.candidagenome.org/download/sequence/Assembly21/current/ | Jones *et al.*, 2004, Skrzypek *et al.*, 2009 |
| *Candida guilliermondii* | ATCC6260 | http://www.broadinstitute.org/annotation/genome/candida_albicans/MultiHome.html | Butler *et al.*, 2009 |
| *Candida lusitaniae* | ATCC 42720 | http://www.broadinstitute.org/annotation/genome/candida_albicans/MultiHome.html | Butler *et al.*, 2009 |
| *Candida parapsilosis* | CDC 317 | http://www.broadinstitute.org/annotation/genome/candida_albicans/MultiHome.html | Butler *et al.*, 2009 |
| *Candida tropicalis* | MYA-3404 | http://www.broadinstitute.org/annotation/genome/candida_albicans/MultiHome.html | Butler *et al.*, 2009 |
| *Lodderomyces elongisporus* | NRRL YB-4239 | http://www.broadinstitute.org/annotation/genome/candida_albicans/MultiHome.html | Butler *et al.*, 2009 |
| *Pichia pastoris* | GS115 | https://bioinformatics.psb.ugent.be/gdb/pichia/ | Mattanovich *et al.*, 2009 |
| *Pichia stipitis* | CBS 6054 | http://genome.jgi-psf.org/Picst3/Picst3.home.html | Jeffries *et al.*, 2007 |
| *Candida dubliniensis* | CD36 | http://www.sanger.ac.uk/sequencing/Candida/dubliniensis/ | Jackson *et al.*, 2009 |
