# Supplementary material for: Pichia sorbitophila, an Interspecies Yeast Hybrid, Reveals Early Steps of Genome Resolution After Polyploidization
Source: G3 (Bethesda). 2012 Feb 1;2(2):299–311. doi: 10.1534/g3.111.000745 (PMC3284337; doi:10.1534/g3.111.000745)
Supplement: Supporting Information [file supp_2.2.299_TableS6.pdf]

Table S6 Distribution of introns in protein-coding genes

Chromosome	Intron-containing genes					Introns
	1 intron	2 introns	3 introns	4 introns	Total	
Piso0A	30	2	1	1	34	41
Piso0B	31	2	1	1	35	42
Piso0C	47	0	0	0	47	47
Piso0D	47	0	0	0	47	47
Piso0E	35	0	1	1	37	42
Piso0F	42	0	1	1	44	49
Piso0G	39	3	0	0	42	45
Piso0H	39	3	0	0	42	45
Piso0I	65	2	0	0	67	69
Piso0J	73	2	0	0	75	77
Piso0K	57	4	1	1	63	72
Piso0L	57	4	1	1	63	72
Piso0M	61	8	0	0	69	77
Piso0N	62	8	0	0	70	78
Genome	685	38	6	4	735	803
