# Supplementary material for: Pichia sorbitophila, an Interspecies Yeast Hybrid, Reveals Early Steps of Genome Resolution After Polyploidization
Source: G3 (Bethesda). 2012 Feb 1;2(2):299–311. doi: 10.1534/g3.111.000745 (PMC3284337; doi:10.1534/g3.111.000745)
Supplement: Supporting Information [file supp_2.2.299_TableS8.pdf]

Table S8 Characteristics of tandemly duplicated gene arrays

species		Nbr. of TGA		total nbr. of CDSs	Nbr. of CDSs in TGA	proportion of CDSs in TGA (%)	Nbr. of TGA in direct orientation	Nbr. of TGA in convergent orientation	Nbr. of TGA in divergent orientation	Nbr. of TGA in mixte orientation
		total	with relic							
Saccharomyces cerevisiae		52	3	5859	111	1.895	42 (80.8)	5 (9.6)	4 (7.7)	1 (1.9)
Candida glabrata		44	1	5200	114	2.192	31 (70.5)	6 (13.6)	3 (6.8)	4 (9.1)
Zygosaccharomyces rouxii		47	8	4991	97	1.943	41 (87.2)	3(6.4)	2 (4.3)	1 (2.1)
Kluyveromyces thermotolerans		37	6	5092	77	1.512	30 (81.1)	4 (10.8)	2 (5.4)	1 (2.7)
Kluyveromyces lactis		36	2	5075	72	1.419	27 (75.0)	4 (11.1)	4 (11.1)	1 (2.8)
Debaryomyces hansenii		128	19	6264	273	4.358	110 (85.9)	8 (6.3)	9 (7.0)	1 (0.8)
Pichia sorbitophila	***hybrid***	**75**	**2**	**11252**	**153**	**1.359**	**51 (68.0)**	**4 (5.3)**	**18 (24.0)**	**2 (2.7)**
	eq. haploid*	**38**	**1**	**5736**	**77**	**1.359**	**25**	**2**	**9**	**1**
Yarrowia lipolytica		43	7	6426	80	1.245	31 (72.1)	4 (9.3)	8 (18.6)	0 (0.0)

*The number of TGA and genes identified in *P. sorbitophila* genome (hybrid) are converted into an equivalent haplotype for a best comparison with the other haploid hemiascomycetous yeasts described.

Numbers in brackets indicate the percentage of each orientation
