# Supplementary material for: Pichia sorbitophila, an Interspecies Yeast Hybrid, Reveals Early Steps of Genome Resolution After Polyploidization
Source: G3 (Bethesda). 2012 Feb 1;2(2):299–311. doi: 10.1534/g3.111.000745 (PMC3284337; doi:10.1534/g3.111.000745)
Supplement: Supporting Information [file supp_2.2.299_TableS14.pdf]

Table S14   tDNA numbers per chromosome in *P. sorbitophila* genome

chr.	Nbr per chr.			in eq. haploid
	Heterozygous	Homozygous	Total	
A	11	10	21	21
B	11	10	21	
C	6	4	10	10
D	6	4	10	
I (I/E)	0	0	0	0
E (I/E)	0	0	0	
J (J/F)	4	0	4	4
F (J/F)	4	0	4	
E (E/F)	17	0	17	17
F (E/F)	17	0	17	
I (I/J)	25	0	25	25
J (I/J)	25	0	25	
G	0	17	17	17
H	0	17	17	
K	0	25	25	25
L	0	25	25	
M	25	0	25	25
N	25	0	25	
**Total**	**176**	**112**	**288**	**144**

Genes encoding tRNAs were searched with tRNAScan-SE (Lowe and Eddy, 1997) and obtained sequences were analyzed for possible exceptions to the eukaryotic cloverleaf model, as described in Marck and Grosjean (2002). Initiator Met tRNA genes were distinguished from the elongator sequences thanks to the "GGG" sequence in positions 29-31. For chr. E/F/I/J, the syntenic heterozygous pairs considered are mentionned in brackets.
