# Supplementary material for: Pichia sorbitophila, an Interspecies Yeast Hybrid, Reveals Early Steps of Genome Resolution After Polyploidization
Source: G3 (Bethesda). 2012 Feb 1;2(2):299–311. doi: 10.1534/g3.111.000745 (PMC3284337; doi:10.1534/g3.111.000745)
Supplement: Supporting Information [file supp_2.2.299_TableS9.pdf]

Table S9 Gene ontology categories for conserved alleles

Molecular_function	Other_genes	Conserved_alleles	Freq. for other genes	Freq. for conserved_alleles	Freq conserved/Freq other
signal transducer activity	56	4	0.01	0.04	5.43
nucleotidyltransferase activity	114	6	0.01	0.06	4.00
structural molecule activity	470	22	0.06	0.21	3.56
RNA binding	328	14	0.04	0.13	3.24
DNA binding	427	12	0.05	0.12	2.14
transcription regulator activity	389	10	0.05	0.10	1.95
peptidase activity	211	4	0.03	0.04	1.44
hydrolase activity	1343	14	0.17	0.13	0.79
protein binding	786	6	0.10	0.06	0.58
ligase activity	286	2	0.04	0.02	0.53
transferase activity	1179	8	0.15	0.08	0.52
transporter activity	686	2	0.09	0.02	0.22
oxidoreductase activity	516	0	0.07	0.00	0.00
lyase activity	148	0	0.02	0.00	0.00
protein kinase activity	206	0	0.03	0.00	0.00
motor activity	18	0	0.00	0.00	0.00
enzyme regulator activity	307	0	0.04	0.00	0.00
lipid binding	126	0	0.02	0.00	0.00
helicase activity	116	0	0.01	0.00	0.00
translation regulator activity	8	0	0.00	0.00	0.00
isomerase activity	99	0	0.01	0.00	0.00
phosphoprotein phosphatase activity	86	0	0.01	0.00	0.00
Total	7905	104	1.00	1.00	1.00
**Cellular_component**	**Other_genes**	**Conserved_alleles**	**Freq. for other genes**	**Freq. for conserved_alleles**	**Freq conserved/Freq other**
ribosome	488	22	0.03	0.10	3.69
chromosome	444	16	0.02	0.07	2.95
cellular bud	267	8	0.01	0.04	2.45
nucleolus	410	12	0.02	0.06	2.39
site of polarized growth	344	8	0.02	0.04	1.90

Golgi apparatus	316	6	0.02	0.03	1.55
peroxisome	108	2	0.01	0.01	1.51
mitochondrial envelope	506	8	0.03	0.04	1.29
cytoskeleton	265	4	0.01	0.02	1.23
nucleus	2761	38	0.15	0.17	1.13
cell cortex	180	2	0.01	0.01	0.91
cytoplasmic membrane-bounded vesicle	193	2	0.01	0.01	0.85
cytoplasm	5420	52	0.30	0.24	0.78
mitochondrion	1746	16	0.10	0.07	0.75
plasma membrane	486	4	0.03	0.02	0.67
membrane fraction	290	2	0.02	0.01	0.56
membrane	1863	12	0.10	0.06	0.53
vacuole	365	2	0.02	0.01	0.45
endoplasmic reticulum	614	2	0.03	0.01	0.27
cell wall	96	0	0.01	0.00	0.00
microtubule organizing center	78	0	0.00	0.00	0.00
endomembrane system	553	0	0.03	0.00	0.00
extracellular region	41	0	0.00	0.00	0.00
Total	17834	218	1.00	1.00	1.00

See Figure S8 for method
