# Supplementary material for: Pichia sorbitophila, an Interspecies Yeast Hybrid, Reveals Early Steps of Genome Resolution After Polyploidization
Source: G3 (Bethesda). 2012 Feb 1;2(2):299–311. doi: 10.1534/g3.111.000745 (PMC3284337; doi:10.1534/g3.111.000745)
Supplement: Supporting Information [file supp_2.2.299_TableS10.pdf]

Table S10 Hypervariable alleles in heterozygous regions

type_1	locus_1	lenght_1	orient.	Orthologs*	%idADN	%idProt	dN/dS	type_2	locus_2	lenght_2	orient.
CDS	PISO0C04424g	867	1	1	85,2	77,1	0,4097	CDS	PISO0D04535g	867	1
CDS	PISO0E04294g	834	-1	1	83,3	80,8	0,3954	CDS	PISO0F05703g	738	-1
CDS	PISO0M22232g	264	-1	1	86,4	80,5	0,344	CDS	PISO0N22475g	264	-1
CDS	PISO0C01894g	1527	-1	1	84,7	78	0,4229	CDS	PISO0D01983g	1530	-1
CDS	PISO0C01124g	1734	1	1	86,1	79,5	0,3492	CDS	PISO0D01213g	1731	1
CDS	PISO0C00486g	576	-1	1	92,4	89,5	0,4371	CDS	PISO0D00575g	576	-1
CDS	PISO0C00420g	1041	-1	1	90,7	87,6	0,3452	CDS	PISO0D00509g	1050	-1
CDS	PISO0E00576g	2349	1	1	84,6	79,8	0,3193	CDS	PISO0I00404g	2292	1
CDS	PISO0I18708g	1089	-1	1	92,7	90,6	0,3115	CDS	PISO0J20469g	1089	-1
CDS	PISO0M24828g	2454	-1	1	83,4	75,2	0,3677	CDS	PISO0N25159g	2454	-1
CDS	PISO0I04078g	930	1	1	90,5	87,1	0,3178	CDS	PISO0J05883g	930	1
CDS	PISO0I04188g	450	1	1	90	85,9	0,3182	CDS	PISO0J05993g	450	1
CDS	PISO0A06710g	651	1	1	88,4	84,1	0,3837	CDS	PISO0B06777g	645	1
CDS	PISO0A00660g	561	-1	1	88,4	80,1	0,7127	CDS	PISO0B00727g	561	-1
CDS	PISO0M20406g	318	1	1	92,1	86,7	0,4538	CDS	PISO0N20649g	318	1
CDS	PISO0M21374g	672	1	1	86,6	77,3	0,5284	CDS	PISO0N21617g	663	1
CDS	PISO0M15170g	2325	1	1	86	79,3	0,3576	CDS	PISO0N15567g	2388	1
CDS	PISO0E11620g	2007	-1	1	85,9	78,4	0,403	CDS	PISO0F13007g	2016	-1
CDS	PISO0E11422g	375	-1	1	91,7	87,1	0,7301	CDS	PISO0F12809g	375	-1
CDS	PISO0E11312g	453	-1	1	84,3	74	0,3748	CDS	PISO0F12699g	453	-1
CDS	PISO0I10678g	1575	-1	1	89,7	86,5	0,3403	CDS	PISO0J12483g	1575	-1
CDS	PISO0I10612g	609	-1	1	92,4	90,6	0,481	CDS	PISO0J12439g	621	-1
CDS	PISO0I06630g	1044	1	1	86,3	80,7	0,3736	CDS	PISO0J08457g	1047	1
CDS	PISO0M02740g	549	-1	1	88,2	84,1	0,312	CDS	PISO0N02873g	561	-1
CDS	PISO0E07198g	627	-1	1	89,5	84,1	0,3981	CDS	PISO0F08541g	627	-1
CDS	PISO0E07814g	1293	-1	1	87,9	82,3	0,4013	CDS	PISO0F09157g	1290	-1
CDS	PISO0E08518g	2262	-1	1	86,9	80,5	0,401	CDS	PISO0F09861g	2253	-1
CDS	PISO0A03190g	276	-1	1	89,5	84,6	0,3256	CDS	PISO0B03301g	276	-1
CDS	PISO0I02054g	2067	1	1	84,3	75,4	0,3875	CDS	PISO0J03837g	2064	1
CDS	PISO0M20890g	675	-1	1	86,4	81,2	0,3232	CDS	PISO0N21133g	675	-1

CDS	PISO0M00474g	912	1	1	83,4	75,5	0,3734	CDS	PISO0N00629g	909	1
CDS	PISO0M14400g	390	-1	1	92,8	88,4	0,417	CDS	PISO0N14797g	390	-1
CDS	PISO0M12706g	354	-1	1	91,8	89,7	0,3258	CDS	PISO0N13015g	354	-1
CDS	PISO0F01237g	819	1	1	88	83,1	0,4351	CDS	PISO0J01219g	819	1
CDS	PISO0I14484g	4455	1	1	88,7	85,3	0,313	CDS	PISO0J16289g	4458	1
CDS	PISO0M11870g	831	-1	1	91,5	88,4	0,3338	CDS	PISO0N12179g	831	-1
CDS	PISO0M13212g	1101	1	1	88,6	84,3	0,4086	CDS	PISO0N13521g	1035	1
CDS	PISO0E13578g	680	-1	0	97,3	96,2	0,4647	CDS	PISO0F14965g	693	-1
CDS	PISO0F01567g	330	1	0	75,8	38,9	1,1931	CDS	PISO0J01549g	273	1
CDS	PISO0M10440g	225	1	0	83,9	68,3	1,1526	CDS	PISO0N10727g	192	1
CDS	PISO0C00992g	339	-1	0	93	76,8	1,0331	CDS	PISO0D01081g	300	-1
CDS	PISO0E06032g	339	-1	1	93,2	91,1	0,6224	CDS	PISO0F07353g	342	-1
CDS	PISO0I05816g	2091	-1	0	80	68,7	0,6116	CDS	PISO0J07599g	2220	-1
CDS	PISO0I12636g	768	-1	1	89,2	82	0,4956	CDS	PISO0J14463g	753	-1
CDS	PISO0A00946g	642	1	0	85	75,6	0,4882	CDS	PISO0B01101g	642	1
CDS	PISO0C04006g	261	1	0	90	67,4	0,479	CDS	PISO0D04095g	273	1
CDS	PISO0M05380g	1008	1	0	83,3	72,2	0,4547	CDS	PISO0N05535g	876	1
CDS	PISO0E11114g	1098	-1	0	90,2	84,7	0,4175	CDS	PISO0F12501g	1119	-1
CDS	PISO0E08892g	1380	1	0	82	70,8	0,4069	CDS	PISO0F10235g	1407	1
CDS	PISO0M05358g	1173	1	0	88,2	82,1	0,4014	CDS	PISO0N05513g	1179	1
CDS	PISO0I05508g	582	-1	1	85,5	83,7	0,3793	CDS	PISO0J07313g	573	-1
CDS	PISO0M11892g	357	-1	1	86,8	80,5	0,3709	CDS	PISO0N12201g	357	-1
CDS	PISO0A05522g	660	1	1	89,4	86,3	0,3517	CDS	PISO0B05589g	660	1
CDS	PISO0E12060g	690	1	1	82,5	78,6	0,5083	CDS	PISO0F13447g	762	1
CDS	PISO0M24542g	1023	-1	1	89,2	86,8	0,3996	CDS	PISO0N24829g	1044	-1
CDS	PISO0C01388g	1743	-1	1	84,2	76,9	0,3699	CDS	PISO0D01477g	1749	-1
CDS	PISO0A04642g	1074	1	1	90,4	85,7	0,3815	CDS	PISO0B04731g	1074	1
CDS	PISO0F01457g	1272	1	1	88,4	82,4	0,3918	CDS	PISO0J01439g	1233	1
CDS	PISO0I10348g	954	1	1	91,8	87,7	0,4854	CDS	PISO0J12175g	954	1
CDS	PISO0I03242g	1185	-1	1	95,6	94,2	0,35	CDS	PISO0J05025g	1185	-1
CDS	PISO0M00672g	1428	-1	1	90,9	88	0,3652	CDS	PISO0N00827g	1404	-1
CDS	PISO0M04060g	363	-1	1	92	78,3	0,403	CDS	PISO0N04193g	375	-1
CDS	PISO0E12808g	1827	-1	1	86,1	78,9	0,4186	CDS	PISO0F14195g	1824	-1
CDS	PISO0I08632g	2853	-1	1	86,7	81,1	0,376	CDS	PISO0J10459g	2859	-1
CDS	PISO0I12394g	2328	-1	1	86,9	80,2	0,4374	CDS	PISO0J14221g	2304	-1
CDS	PISO0E05812g	2379	1	1	82,6	75,6	0,3886	CDS	PISO0F07133g	2220	1

CDS	PISO0E08232g	786	1	1	91,1	86,2	0,7959	CDS	PISO0F09575g	786	1
CDS	PISO0I11074g	3219	1	1	86,5	80,1	0,3858	CDS	PISO0J12879g	3243	1
CDS	PISO0I17058g	495	1	1	95,4	94,5	0,3123	CDS	PISO0J18819g	438	1
CDS	PISO0M04830g	201	1	0	91,5	84,8	0,8357	CDS	PISO0N04941g	201	1
CDS	PISO0M03774g	366	-1	1	83,6	76	0,6206	CDS	PISO0N03907g	384	-1
CDS	PISO0E03128g	1155	-1	0	83,8	73,2	0,506	CDS	PISO0F04537g	1155	-1
CDS	PISO0M14906g	732	1	0	84	75	0,5017	CDS	PISO0N15303g	687	1
CDS	PISO0M14862g	438	1	1	87,8	76,8	0,4723	CDS	PISO0N15259g	378	1
CDS	PISO0M05182g	3654	-1	0	82,2	71,7	0,4399	CDS	PISO0N05337g	3609	-1
CDS	PISO0M00430g	2364	1	0	83,6	75,3	0,4023	CDS	PISO0N00585g	2364	1
CDS	PISO0M10088g	2898	1	0	85,8	78	0,3887	CDS	PISO0N10397g	2898	1
CDS	PISO0M09076g	1413	-1	0	86,7	79,8	0,3842	CDS	PISO0N09275g	1446	-1
CDS	PISO0M13630g	963	-1	1	87	80,3	0,3699	CDS	PISO0N13983g	966	-1
CDS	PISO0I15320g	966	-1	1	90,1	85	0,3694	CDS	PISO0J17125g	966	-1
CDS	PISO0I13142g	1125	1	1	88,4	84,5	0,3428	CDS	PISO0J14947g	1125	1
CDS	PISO0M22122g	369	-1	1	86,2	82	0,328	CDS	PISO0N22365g	372	-1
CDS	PISO0M20296g	303	-1	1	88,4	84	0,3215	CDS	PISO0N20539g	303	-1
CDS	PISO0I15210g	324	1	1	89,5	85	0,3857	CDS	PISO0J17015g	324	1
CDS	PISO0M21264g	945	-1	1	83,4	73,9	0,4148	CDS	PISO0N21507g	933	-1
CDS	PISO0I18796g	1203	1	1	88,5	84	0,3375	CDS	PISO0J20557g	1227	1
CDS	PISO0C01630g	1284	-1	1	89,1	84,3	0,4819	CDS	PISO0D01719g	1308	-1
CDS	PISO0I05442g	399	1	1	91,7	89,4	0,3506	CDS	PISO0J07247g	399	1
CDS	PISO0E01654g	498	-1	1	85,1	79,4	0,4916	CDS	PISO0I01482g	498	-1
CDS	PISO0M20098g	1374	-1	1	86,5	78,6	0,4618	CDS	PISO0N20341g	1386	-1

Both alleles of a gene (_1 and _2) are on the same line. They were compared for their identity in DNA (%idADN), in protein (%idProt), for the dN/dS ratio and the presence of at least one ortholog in the CTG group* (if yes=1). Percentages of identity were calculated for the aligned part of the sequences
