# Supplementary material for: Pichia sorbitophila, an Interspecies Yeast Hybrid, Reveals Early Steps of Genome Resolution After Polyploidization
Source: G3 (Bethesda). 2012 Feb 1;2(2):299–311. doi: 10.1534/g3.111.000745 (PMC3284337; doi:10.1534/g3.111.000745)
Supplement: Supporting Information [file supp_2.2.299_TableS11.pdf]

Table S11 Gene ontology categories for hypervariable alleles

Molecular_function	Other_genes	Hypervariable_alleles	Freq. for other genes	Freq. for hypervariable alleles	Freq hypervariable/Freq other
phosphoprotein phosphatase activity	84	2	0.01	0.05	4.87
structural molecule activity	486	6	0.06	0.15	2.52
isomerase activity	98	1	0.01	0.03	2.09
DNA binding	435	4	0.05	0.10	1.88
hydrolase activity	1347	10	0.17	0.26	1.52
ligase activity	286	2	0.04	0.05	1.43
enzyme regulator activity	305	2	0.04	0.05	1.34
RNA binding	340	2	0.04	0.05	1.20
protein binding	788	4	0.10	0.10	1.04
oxidoreductase activity	514	2	0.06	0.05	0.80
transferase activity	1183	4	0.15	0.10	0.69
signal transducer activity	60	0	0.01	0.00	0.00
peptidase activity	215	0	0.03	0.00	0.00
lyase activity	148	0	0.02	0.00	0.00
transcription regulator activity	399	0	0.05	0.00	0.00
protein kinase activity	206	0	0.03	0.00	0.00
motor activity	18	0	0.00	0.00	0.00
transporter activity	688	0	0.09	0.00	0.00
lipid binding	126	0	0.02	0.00	0.00
helicase activity	116	0	0.01	0.00	0.00
translation regulator activity	8	0	0.00	0.00	0.00
nucleotidyltransferase activity	120	0	0.02	0.00	0.00
Total	7970	39	1.00	1.00	1.00

Cellular_component	Other_genes	Hypervariable_alleles	Freq. for other genes	Freq. for hypervariable alleles	Freq hypervariable/Freq other
extracellular region	37	4	0.00	0.03	16.72
cell wall	90	6	0.01	0.05	10.31
microtubule organizing center	76	2	0.00	0.02	4.07
endoplasmic reticulum	606	10	0.03	0.09	2.55
endomembrane system	545	8	0.03	0.07	2.27
membrane fraction	288	4	0.02	0.03	2.15
Ribosome	504	6	0.03	0.05	1.84

cell cortex	180	2	0.01	0.02	1.72
Vacuole	363	4	0.02	0.03	1.70
Nucleolus	418	4	0.02	0.03	1.48
Cytoskeleton	267	2	0.01	0.02	1.16
cellular bud	273	2	0.02	0.02	1.13
Nucleus	2779	20	0.15	0.17	1.11
Golgi apparatus	320	2	0.02	0.02	0.97
Cytoplasm	5440	32	0.30	0.28	0.91
site of polarized growth	350	2	0.02	0.02	0.88
Membrane	1871	4	0.10	0.03	0.33
Mitochondrion	1760	2	0.10	0.02	0.18
Peroxisome	110	0	0.01	0.00	0.00
Chromosome	460	0	0.03	0.00	0.00
cytoplasmic membrane-bounded vesicle	195	0	0.01	0.00	0.00
plasma membrane	490	0	0.03	0.00	0.00
mitochondrial envelope	514	0	0.03	0.00	0.00
Total	17936	116	1.00	1.00	1.00

See Figure 8 for method
