# Supplementary material for: Pichia sorbitophila, an Interspecies Yeast Hybrid, Reveals Early Steps of Genome Resolution After Polyploidization
Source: G3 (Bethesda). 2012 Feb 1;2(2):299–311. doi: 10.1534/g3.111.000745 (PMC3284337; doi:10.1534/g3.111.000745)
Supplement: Supporting Information [file supp_2.2.299_TableS12.pdf]

Table S12 Gene ontology categories for pseudogenes-CDS pairs of genes

Table S12. Gene ontology categories for pseudogenes-CDS pairs of genes

Molecular_function	Other_genes	Pseudogenes	Freq. for other genes	Freq. for pseudogenes	Freq pseudo/Freq other
oxidoreductase activity	514	2	0.06	0.40	6.23
transporter activity	686	2	0.09	0.40	4.67
transcription regulator activity	398	1	0.05	0.20	4.02
signal transducer activity	60	0	0.01	0.00	0.00
DNA binding	439	0	0.05	0.00	0.00
peptidase activity	215	0	0.03	0.00	0.00
lyase activity	148	0	0.02	0.00	0.00
structural molecule activity	492	0	0.06	0.00	0.00
transferase activity	1187	0	0.15	0.00	0.00
protein kinase activity	206	0	0.03	0.00	0.00
motor activity	18	0	0.00	0.00	0.00
hydrolase activity	1357	0	0.17	0.00	0.00
enzyme regulator activity	307	0	0.04	0.00	0.00
lipid binding	126	0	0.02	0.00	0.00
helicase activity	116	0	0.01	0.00	0.00
translation regulator activity	8	0	0.00	0.00	0.00
ligase activity	288	0	0.04	0.00	0.00
nucleotidyltransferase activity	120	0	0.01	0.00	0.00
isomerase activity	99	0	0.01	0.00	0.00
phosphoprotein phosphatase activity	86	0	0.01	0.00	0.00
RNA binding	342	0	0.04	0.00	0.00
protein binding	792	0	0.10	0.00	0.00
Total	8004	5	1.00	1.00	1.00
**Cellular_component**	**Other_genes**	**Pseudogenes**	**Freq. for other genes**	**Freq. for pseudogenes**	**Freq pseudo/Freq other**
plasma membrane	488	2	0.03	0.14	5.28
mitochondrial envelope	513	1	0.03	0.07	2.51
Membrane	1872	3	0.10	0.21	2.06
Mitochondrion	1760	2	0.10	0.14	1.46
Nucleus	2796	3	0.16	0.21	1.38
Cytoplasm	5469	3	0.30	0.21	0.71
endoplasmic reticulum	616	0	0.03	0.00	0.00
site of polarized growth	352	0	0.02	0.00	0.00

Nucleolus	422	0	0.02	0.00	0.00
cell wall	96	0	0.01	0.00	0.00
Peroxisome	110	0	0.01	0.00	0.00
microtubule organizing center	78	0	0.00	0.00	0.00
Chromosome	460	0	0.03	0.00	0.00
Golgi apparatus	322	0	0.02	0.00	0.00
cytoplasmic membrane-bounded vesicle	195	0	0.01	0.00	0.00
endomembrane system	553	0	0.03	0.00	0.00
cellular bud	275	0	0.02	0.00	0.00
Ribosome	510	0	0.03	0.00	0.00
membrane fraction	292	0	0.02	0.00	0.00
Cytoskeleton	269	0	0.01	0.00	0.00
Vacuole	367	0	0.02	0.00	0.00
extracellular region	41	0	0.00	0.00	0.00
cell cortex	182	0	0.01	0.00	0.00
Total	18038	14	1.00	1.00	1.00

See Figure 8 for method
