# Supplementary material for: Pichia sorbitophila, an Interspecies Yeast Hybrid, Reveals Early Steps of Genome Resolution After Polyploidization
Source: G3 (Bethesda). 2012 Feb 1;2(2):299–311. doi: 10.1534/g3.111.000745 (PMC3284337; doi:10.1534/g3.111.000745)
Supplement: Supporting Information [file supp_2.2.299_TableS13.pdf]

Table S13 Gene ontology categories for single allele genes

Molecular_function	Other_genes	Single_alleles	Freq. for other genes	Freq. for single_alleles	Freq single/Freq other
transporter activity	664	24	0.08	0.39	4.71
oxidoreductase activity	506	10	0.06	0.16	2.58
transcription regulator activity	392	7	0.05	0.11	2.33
DNA binding	435	4	0.05	0.07	1.20
hydrolase activity	1346	11	0.17	0.18	1.06
peptidase activity	214	1	0.03	0.02	0.61
protein binding	790	2	0.10	0.03	0.33
transferase activity	1185	2	0.15	0.03	0.22
signal transducer activity	60	0	0.01	0.00	0.00
lyase activity	148	0	0.02	0.00	0.00
structural molecule activity	492	0	0.06	0.00	0.00
protein kinase activity	206	0	0.03	0.00	0.00
motor activity	18	0	0.00	0.00	0.00
enzyme regulator activity	307	0	0.04	0.00	0.00
lipid binding	126	0	0.02	0.00	0.00
helicase activity	116	0	0.01	0.00	0.00
translation regulator activity	8	0	0.00	0.00	0.00
ligase activity	288	0	0.04	0.00	0.00
nucleotidyltransferase activity	120	0	0.02	0.00	0.00
isomerase activity	99	0	0.01	0.00	0.00
phosphoprotein phosphatase activity	86	0	0.01	0.00	0.00
RNA binding	342	0	0.04	0.00	0.00
Total	7948	61	1.00	1.00	1.00
**Cellular_component**	**Other_genes**	**Single_alleles**	**Freq. for other genes**	**Freq. for single_alleles**	**Freq single/Freq other**
extracellular region	38	3	0.00	0.03	12.00
plasma membrane	471	19	0.03	0.16	6.13
cell wall	94	2	0.01	0.02	3.23
Membrane	1851	24	0.10	0.20	1.97
membrane fraction	289	3	0.02	0.03	1.58
cytoplasmic membrane-bounded vesicle	193	2	0.01	0.02	1.57
Vacuole	364	3	0.02	0.03	1.25
Mitochondrion	1748	14	0.10	0.12	1.22
Ribosome	506	4	0.03	0.03	1.20

Cytoplasm	5442	30	0.30	0.25	0.84
cellular bud	274	1	0.02	0.01	0.55
endoplasmic reticulum	614	2	0.03	0.02	0.50
Nucleus	2790	9	0.16	0.08	0.49
mitochondrial envelope	513	1	0.03	0.01	0.30
endomembrane system	552	1	0.03	0.01	0.28
site of polarized growth	352	0	0.02	0.00	0.00
Nucleolus	422	0	0.02	0.00	0.00
Peroxisome	110	0	0.01	0.00	0.00
microtubule organizing center	78	0	0.00	0.00	0.00
Chromosome	460	0	0.03	0.00	0.00
Golgi apparatus	322	0	0.02	0.00	0.00
Cytoskeleton	269	0	0.01	0.00	0.00
cell cortex	182	0	0.01	0.00	0.00
Total	17934	118	1.00	1.00	1.00

See Figure 8 for method
