# Supplementary material for: Pichia sorbitophila, an Interspecies Yeast Hybrid, Reveals Early Steps of Genome Resolution After Polyploidization
Source: G3 (Bethesda). 2012 Feb 1;2(2):299–311. doi: 10.1534/g3.111.000745 (PMC3284337; doi:10.1534/g3.111.000745)
Supplement: Supporting Information [file supp_2.2.299_TableS15.pdf]

Table S15 Pairs of potentially co-transcribed tRNA genes in *P. sorbitophila*

tDNA pairs and intervals in genome	nb of occurrences	
tDNA-Asp (GTC)-{10 or 11 nt}-tDNA-Gly (GCC)	x4	*
tDNA-Ile (AAT)-{11 or 13 nt}-tDNA-Ala (AGC)	x4	*
tDNA-Asn (GTT)-{9 nt}-tDNA-Thr (AGT)	x1	
tDNA-Val (AAC)-{8 nt}-tDNA-Gly (GCC)	x1	*

This table lists the pairs of neighbouring and co-oriented tRNA genes. The tRNA type and distance between tRNA genes (number of nucleotides between base 73 of the first tRNA gene and base 1 of the following gene) are indicated. These very short intervals suggest that the two tandem tDNAs are co-transcribed (Dujon *et al*., 2004). * Pairs also present in the genome of *Y. lipolytica.*
