# Supplementary material for: Pichia sorbitophila, an Interspecies Yeast Hybrid, Reveals Early Steps of Genome Resolution After Polyploidization
Source: G3 (Bethesda). 2012 Feb 1;2(2):299–311. doi: 10.1534/g3.111.000745 (PMC3284337; doi:10.1534/g3.111.000745)
Supplement: Supporting Information [file supp_2.2.299_TableS16.pdf]

Table S16 Codon and tRNA gene usages in *P. sorbitophila* and *D. hansenii*

	AA	C	AC	*P.sorbitophila*		*D. hansenii*	
F	Phe	TTT	---	2.27	-	2.64	-
F	Phe	TTC	(GAA)	2.14	5+	1.82	8+
L	Leu	TTA	(TAA)	2.19	2	3.64	9
L	Leu	TTG	(CAA)	3.32	7	3.11	4
L	Leu	CTT	(AAG)	1.77	2	1.21	2
L	Leu	CTC	(GAG)	1.13	-	0.47	-
L	Leu	CTA	(TAG)	0.90	-	0.78	-
S	Ser	CTG	(CAG)	0.66	1	0.41	1
I	Ile	ATT	(AAT)	2.37	6	3.45	9
I	Ile	ATC	(GAT)	1.82	-	1.53	-
I	Ile	ATA	(TAT)	2.15	2+	2.07	2+
M	Met	ATG	(CAT)	1.73	3+	0.21	4+
m	iMet	ATG	(CAT)	0.21	2	1.79	3
V	Val	GTT	(AAC)	1.94	6	2.38	10
V	Val	GTC	---	1.27	-	0.94	-
V	Val	GTA	(TAC)	1.13	1+	1.14	1+
V	Val	GTG	(CAC)	1.41	2	0.96	1
S	Ser	TCT	(AGA)	2.23	5	2.16	6
S	Ser	TCC	---	1.21	-	0.99	-
S	Ser	TCA	(TGA)	1.73	2	2.13	3
S	Ser	TCG	(CGA)	1.27	1	1.31	1+
P	Pro	CCT	(AGG)	1.55	1	1.33	1
P	Pro	CCC	---	0.79	-	0.43	-
P	Pro	CCA	(TGG)	1.48	4+	1.99	7+
P	Pro	CCG	(CGG)	0.45	-	0.49	-
T	Thr	ACT	(AGT)	1.62	5	1.95	8
T	Thr	ACC	---	1.14	-	1.09	-
T	Thr	ACA	(TGT)	1.60	2	1.66	2
T	Thr	ACG	(CGT)	0.84	1	0.78	1
A	Ala	GCT	(AGC)	1.99	6	1.95	7

A	Ala	GCC	---	1.24	-	0.92	-
A	Ala	GCA	(TGC)	1.71	4	1.73	4
A	Ala	GCG	(CGC)	0.77	1	0.56	1
Y	Tyr	TAT		1.77	-	2.24	-
Y	Tyr	TAC	(GTA)	1.76	5+	1.36	6+
*	Och	TAA	---		-		-
*	Amb	TAG	---		-		-
H	His	CAT	---	1.28	-	1.46	-
H	His	CAC	(GTG)	0.91	4	0.63	5
Q	Gln	CAA	(TTG)	1.91	4	2.68	7
Q	Gln	CAG	(CTG)	1.72	2	1.13	1
N	Asn	AAT	---	3.08	-	4.44	-
N	Asn	AAC	(GTT)	2.65	6	2.22	8
K	Lys	AAA	(TTT)	3.47	3+	3.92	7+
K	Lys	AAG	(CTT)	3.82	5+	3.47	10+
D	Asp	GAT	---	3.46	-	4.33	-
D	Asp	GAC	(GTC)	2.59	7	1.85	9
E	Glu	GAA	(TTC)	3.84	4	4.83	9
E	Glu	GAG	(CTC)	2.86	4	1.75	1
C	Cys	TGT	---	0.66	-	0.81	-
C	Cys	TGC	(GCA)	0.53	2	0.35	4
*	Opa	TGA	---		-		-
W	Trp	TGG	(CCA)	1.00	3	0.99	4
R	Arg	CGT	(ACG)	0.59	2+	0.53	4+
R	Arg	CGC	---	0.38	-	0.13	-
R	Arg	CGA	(TCG)	0.39	-	0.27	-
R	Arg	CGG	(CCG)	0.26	1+	0.17	1+
S	Ser	AGT	---	1.35	-	1.53	-
S	Ser	AGC	(GCT)	1.34	3+	0.77	3+
R	Arg	AGA	(TCT)	2.09	5	2.24	9
R	Arg	AGG	(CCT)	0.89	1	0.68	1
G	Gly	GGT	---	1.97	-	2.30	-
G	Gly	GGC	(GCC)	1.10	8	0.69	11

G	Gly	GGA	(TCC)	1.67	3	1.52	4
G	Gly	GGG	(CCC)	0.67	1	0.69	1

The first column (AA) indicates the charged amino acid (in one- and three-letter codes), the second one (C) the codon and the third one (AC) the anticodon found (between brackets). For each genome, the first value is the codon usage (in % of the 61 sense codons) and the second one the number of tRNA genes ("-" no tRNA gene). The "+" signs denote genes with intron. The number of tRNA indicated refers to the theoretical haploid genome. The codon usage refers to the complete diploid genome in order to average the heterozygosity. Data for *Debaryomyces hansenii* are revised data from Dujon *et al*., 2004.
