# Supplementary material for: Pichia sorbitophila, an Interspecies Yeast Hybrid, Reveals Early Steps of Genome Resolution After Polyploidization
Source: G3 (Bethesda). 2012 Feb 1;2(2):299–311. doi: 10.1534/g3.111.000745 (PMC3284337; doi:10.1534/g3.111.000745)
Supplement: Supporting Information [file supp_2.2.299_TableS17.pdf]

Table S17 List of ncRNA genes

ncRNA class	Gene_name	Allele_1				%identity*	Allele_2			Area**
		Allele1	Subgenome	Locus1	Annotation		Allele2	Subgenome	Locus2	
snRNA	Piso0_001482	Piso0_001482-1	Pε	Piso0E06043r	snRNA SNR19 (U1)	84.6	Piso0_001482-2	Pγ	PISO0F07375r	he
	Piso0_003856	Piso0_003856-1	Pγ	PISO0K04080r	snRNA SNR20 (U2)	100.0	Piso0_003856-1	Pγ	PISO0L04081r	hm
	Piso0_005802	Piso0_005802-1	Pε	PISO0M22672r	snRNA SNR14 (U4)	98.2	Piso0_005802-2	Pγ	PISO0N22915r	he
	Piso0_005825	Piso0_005825-1	Pε	PISO0M23134r	snRNA SNR7 (U5)	98.4	Piso0_005825-2	Pγ	PISO0N23421r	he
snoRNA	Piso0_003217	Piso0_003217-1	Pγ	PISO0G07464r	snoRNA snR5	100.0	Piso0_003217-1	Pγ	PISO0H07465r	hm
	Piso0_004120	Piso0_004120-1	Pγ	PISO0K09888r	snoRNA SNR8	100.0	Piso0_004120-1	Pγ	PISO0L09889r	hm
	Piso0_000301	Piso0_000301-1	Pγ	PISO0A06336r	snoRNA SNR10	98.3	Piso0_000301-2	Pε	PISO0B06403r	he
	Piso0_003392	Piso0_003392-1	Pγ	PISO0G11314r	snoRNA SNR128	100.0	Piso0_003392-1	Pγ	PISO0H11315r	hm
	Piso0_005688	Piso0_005688-1	Pε	PISO0M20164r	snoRNA SNR17	98.9	Piso0_005688-2	Pγ	PISO0N20407r	he
	Piso0_003431	Piso0_003431-1	Pγ	PISO0G12172r	snoRNA SNR18	100.0	Piso0_003431-1	Pγ	PISO0H12173r	hm
	Piso0_000022	Piso0_000022-1	Pγ	PISO0A00440r	snoRNA SNR191	92.5	Piso0_000022-2	Pε	PISO0B00507r	he
	Piso0_004900	Piso0_004900-1	Pε	PISO0M03422r	snoRNA SNR24	100.0	Piso0_004900-1	Pγ	PISO0N03555r	he
	Piso0_000550	Piso0_000550-1	Pε	PISO0A11814r	snoRNA SNR32	100.0	Piso0_000550-1	Pε	PISO0B11881r	hm
	Piso0_002702	Piso0_002702-1	Pγ	PISO0I15936r	snoRNA SNR35	99.0	Piso0_002702-2	Pε	PISO0J17741r	he
	Piso0_005801	Piso0_005801-1	Pε	PISO0M22650r	snoRNA SNR36	95.6	Piso0_005801-2	Pγ	PISO0N22893r	he
	Piso0_005896	Piso0_005896-1	Pε	PISO0M24652r	snoRNA SNR37	97.9	Piso0_005896-2	Pγ	PISO0N24939r	he
	Piso0_001254	Piso0_001254-1	Pε	PISO0E01060r	snoRNA SNR38	91.0	Piso0_001254-2	Pγ	PISO0I00888r	he
	Piso0_005580	Piso0_005580-1	Pε	PISO0M17788r	snoRNA SNR40	100.0	Piso0_005580-1	Pγ	PISO0N18031r	he
	Piso0_005631	Piso0_005631-1	Pε	PISO0M18910r	snoRNA SNR41	97.9	Piso0_005631-2	Pγ	PISO0N19153r	he
	Piso0_002238	Piso0_002238-1	Pγ	PISO0I05871r	snoRNA SNR42	95.6	Piso0_002238-2	Pε	PISO0J07665r	he
	Piso0_001592	Piso0_001592-1	Pε	PISO0E08430r	snoRNA SNR43	94.7	Piso0_001592-2	Pγ	PISO0F09773r	he
	Piso0_002636	Piso0_002636-1	Pγ	PISO0I14506r	snoRNA SNR47	100.0	Piso0_002636-1	Pε	PISO0J16311r	he
	Piso0_003338	Piso0_003338-1	Pγ	PISO0G10126r	snoRNA SNR49	100.0	Piso0_003338-1	Pγ	PISO0H10127r	hm
	Piso0_005632	Piso0_005632-1	Pε	PISO0M18932r	snoRNA SNR51	98.9	Piso0_005632-2	Pγ	PISO0N19175r	he
	Piso0_001150	Piso0_001150-1	Pγ	PISO0C11992r	snoRNA SNR54	100.0	Piso0_001150-1	Pγ	PISO0D12059r	hm
	Piso0_002436	Piso0_002436-1	Pγ	PISO0I10128r	snoRNA SNR55	96.1	Piso0_002436-2	Pε	PISO0J11955r	he
	Piso0_004621	Piso0_004621-1	Pγ	PISO0K20910r	snoRNA SNR56	100.0	Piso0_004621-1	Pγ	PISO0L20911r	hm
	Piso0_002437	Piso0_002437-1	Pγ	PISO0I10150r	snoRNA SNR57	91.3	Piso0_002437-2	Pε	PISO0J11977r	he
	Piso0_002435	Piso0_002435-1	Pγ	PISO0I10106r	snoRNA SNR61	98.9	Piso0_002435-2	Pε	PISO0J11933r	he
	Piso0_000459	Piso0_000459-1	Pε	PISO0A09812r	snoRNA SNR62	100.0	Piso0_000459-1	Pε	PISO0B09879r	hm
	Piso0_001366	Piso0_001366-1	Pε	PISO0E03502r	snoRNA SNR66	93.1	Piso0_001366-2	Pγ	PISO0F04911r	he
	Piso0_005068	Piso0_005068-1	Pε	PISO0M07030r	snoRNA SNR67	98.9	Piso0_005068-2	Pγ	PISO0N07163r	he
	Piso0_005140	Piso0_005140-1	Pε	PISO0M08504r	snoRNA SNR71	96.6	Piso0_005140-2	Pγ	PISO0N08681r	he
	Piso0_003377	Piso0_003377-1	Pγ	PISO0G10984r	snoRNA SNR73	100.0	Piso0_003377-1	Pγ	PISO0H10985r	hm
	Piso0_003376	Piso0_003376-1	Pγ	PISO0G10962r	snoRNA SNR74	100.0	Piso0_003376-1	Pγ	PISO0H10963r	hm

	Piso0_003375	Piso0_003375-1	Pγ	PISO0G10940r	snoRNA SNR75	100.0	Piso0_003375-1	Pγ	PISO0H10941r	hm
	Piso0_003374	Piso0_003374-1	Pγ	PISO0G10918r	snoRNA SNR76	100.0	Piso0_003374-1	Pγ	PISO0H10919r	hm
	Piso0_003373	Piso0_003373-1	Pγ	PISO0G10896r	snoRNA SNR77	100.0	Piso0_003373-1	Pγ	PISO0H10897r	hm
	Piso0_003372	Piso0_003372-1	Pγ	PISO0G10874r	snoRNA SNR78	100.0	Piso0_003372-1	Pγ	PISO0H10875r	hm
	Piso0_005706	Piso0_005706-1	Pε	PISO0M20560r	snoRNA SNR79	98.9	Piso0_005706-2	Pγ	PISO0N20803r	he
	Piso0_000435	Piso0_000435-1	Pε	PISO0A09284r	snoRNA SNR80	100.0	Piso0_000435-1	Pε	PISO0B09351r	hm
Polymerase III transcripts										
snRNA	Piso0_001264	Piso0_001264-1	Pε	PISO0E01280r	SNR6 (U6 RNA)	100.0	Piso0_001264-1	Pγ	PISO0I01108r	he
SRP RNA	Piso0_004927	Piso0_004927-1	Pε	PISO0M04016r	SCR1 (SRP RNA)	98.0	Piso0_004927-2	Pγ	PISO0N04149r	he
RNaseP RNA	Piso0_005211	Piso0_005211-1	Pε	PISO0M09890r	RPR1 (RNase P RNA)	94.4	Piso0_005211-2	Pγ	PISO0N10199r	he
Rnase MRP RNA	Piso0_001365	Piso0_001365-1	Pε	PISO0E03480r	RNase_MRP	93.0	Piso0_001365-2	Pγ	PISO0F04889r	he
snoRNA	Piso0_001993	Piso0_001993-1	Pγ	PISO0F02403r	SNR52	97.1	Piso0_001993-2	Pε	PISO0J02363r	he

Non-coding RNA (ncRNA) genes were detected using the following bioinformatics tools: 1) ncRNA sequences from the Genolevures database (Sherman *et al.*, 2009) were used as queries for BLAST (Altschul *et al.*, 1990) searches and hits with an e-value smaller than 0.1 were retained for validation. 2) covariance models found in RFam (Griffiths-Jones, 2009) database were used to perform Infernal searches (Nawrocki *et al.*, 2009). All hits with an e-value smaller than 0.5 were retained for validation. All retained hits were manually checked. Hits were accepted as candidates if: i) the sequence agrees with known structural features, guiding sequences (for snoRNAs) and conserved sequence motifs for homologous molecules or ii) known synteny. * % of identity between both alleles of a ncRNA gene. ** position in heterozygous (he) or homozygous (hm) regions
