# Supplementary material for: Pichia sorbitophila, an Interspecies Yeast Hybrid, Reveals Early Steps of Genome Resolution After Polyploidization
Source: G3 (Bethesda). 2012 Feb 1;2(2):299–311. doi: 10.1534/g3.111.000745 (PMC3284337; doi:10.1534/g3.111.000745)
Supplement: Supporting Information [file supp_2.2.299_TableS18.pdf]

Table S18 ***P. sorbitophila*** **genes in osmotic stress**

transporter type	transporter name	locus_1*	Subgenome_1	locus_2*	Subgenome_2
K+ and Na+ efflux	ENA1	PISO0C05656g	Pγ	PISO0D05745g	Pε
	ENA2	-		-	
K+/H+ antiporter	KHA1	PISO0M11650g	Pε	PISO0N11959g	Pγ
Na+/H+ antiporter	NHA1	PISO0I14000g	Pγ	PISO0J15805g	Pε
K+ and Na+ influx	TRK1	PISO0E03788g	Pε	PISO0F05197g	Pγ
	HAK1	PISO0K12792g	Pγ	PISO0L12793g	Pγ
P-Type ATPase	ACU1	PISO0A12650g	Pε	PISO0B12717g	Pε
H+-ATPase	PMA1	PISO0E05438g	Pε	PISO0F06759g	Pγ
	VMA2	PISO0E12390g	Pε	PISO0F13799g	Pγ
	PHO89	-		-	
H+/glycerol symport	STL1	PISO0A00352g	Pγ	PISO0B00419g	Pε
Glycerol	GPD	Piso0I12878g	Pγ	Piso0J14683g	Pε
	Gup1	PISO0A06402g	Pγ	PISO0B06469g	Pε
Intracellular Na+/K+ concentration	HAL1	-		-	
Membrane Pεrmeability to K+	TOK1	PISO0I13780g	Pγ	PISO0J15585g	Pε
Aquaglyceroporin	FPS1	-		-	
Na+ transport to vacuole	NHX1	PISO0C09154g	Pγ	PISO0D09221g	Pγ
Water flux	AQY1	PISO0G17386g	Pγ	PISO0H17387g	Pγ
HOG pathway	SLN1	PISO0A11462g	Pε	PISO0B11529g	Pε
	SHO1	PISO0L19899g	Pγ	PISO0K19898g	Pγ
	YPD1	PISO0G11886g	Pγ	PISO0H11887g	Pγ
	SSK1	PISO0M05886g	Pε	PISO0N06041g	Pγ
	SSK2	PISO0M09142g	Pε	PISO0N09363g	Pγ
	STE20	PISO0E12786g	Pε	PISO0F14173g	Pγ
	STE50	PISO0A09240g	Pε	PISO0B09307g	Pε
	STE11	PISO0A07018g	Pε	PISO0B07085g	Pε
	PBS2	PISO0E12962g	Pε	PISO0F14349g	Pγ
	HOG1	PISO0E11840g	Pε	PISO0F13227g	Pγ
	SKO1	PISO0K05598g	Pγ	PISO0L05599g	Pγ
	HOT1	-		-	
	RCK2	PISO0G15846g	Pγ	PISO0H15847g	Pγ
	MSN2/4	PISO0E05768g	Pε	PISO0F07089g	Pγ
	SMP1	-		-	
	MSN1	-		-	
	SGD1	PISO0G09928g	Pγ	PISO0H09929g	Pγ
	SKN7	PISO0M12882g	Pε	PISO0N13191g	Pγ
	TUP1	PISO0A11044g	Pε	PISO0B11111g	Pε

	SSN6	PISO0J01747g	Pε	PISO0F01787g	Pγ
Calcineurin	CNA1	PISO0K05268g	Pγ	PISO0L05269g	Pγ
	CNB1	PISO0G03218g	Pγ	PISO0H03219g	Pγ
	CMD1	PISO0K04432g	Pγ	PISO0L04433g	Pγ
	RCN1	-		-	
	CRZ1	PISO0E04118g	Pε	PISO0F05527g	Pγ
	PPZ1	PISO0K14706g	Pγ	PISO0L14707g	Pγ
	HAL3	PISO0I00778g	Pγ	PISO0E00950g	
	HAL4	PISO0E07858g	Pε	PISO0F09201g	Pγ
stress respons and nutrient-controlled signalling	SNF1	PISO0M19570g	Pε	PISO0N19813g	Pγ
	SNF4	PISO0M12244g	Pε	PISO0N12553g	Pγ
	GAL83	PISO0E03392g	Pε	PISO0F04801g	Pγ
	MLG1	PISO0A10670g	Pε	PISO0B10737g	Pε
	SD1	PISO0I19610g	Pγ	PISO0J21371g	Pε
	MTH1	-		-	
	SNF3	PISO0C01740g	Pε	PISO0D01829g	Pγ
	SNF12	PISO0A11528g	Pε	PISO0B11595g	Pε
	SNF2	PISO0C11310g	Pγ	PISO0D11377g	Pγ
	TOR1/2	PISO0G15538g	Pγ	PISO0H15539g	Pγ
	GCN4	PISO0C02158g	Pε	PISO0D02247g	Pγ
	RIM101	PISO0J01571g	Pε	PISO0F01589g	Pγ
	GLN3	PISO0A01408g	Pγ	PISO0B01585g	Pε
	GAT1	PISO0A00814g	Pγ	PISO0B00881g	Pε
	GAT2	PISO0N06899g	Pγ	PISO0M06766g	Pε

* Locus_1 and _2 indicated the locus name of allele in one chromosome forming a pair.
