# Supplementary material for: Pichia sorbitophila, an Interspecies Yeast Hybrid, Reveals Early Steps of Genome Resolution After Polyploidization
Source: G3 (Bethesda). 2012 Feb 1;2(2):299–311. doi: 10.1534/g3.111.000745 (PMC3284337; doi:10.1534/g3.111.000745)
Supplement: Supporting Information [file supp_2.2.299_TableS19.pdf]

Table S19 Conservation of mating and meiosis genes¥

	S. cerevisiae	*D. hansenii**	*D. hansenii_2***	*P.sorbitophila_1*	*P.sorbitophila_2*
**Mating**					
a-factor	YDR461W/YNL145W	No	No	No	No
alpha-factor	YPL187W/YGL089C	DEHA0F20900g	DEHA2F19580g	PISO0G09378g	PISO0H09379g
RAM2	YKL019W	DEHA0F12034g	DEHA2F11022g	PISO0K09932g	PISO0L09933g
KEX1	YGL203C	yes	DEHA2F22352g	PISO0N03137g	PISO0M02982g
KEX2	YNL238W	DEHA0C11308g	DEHA2C10296g	PISO0G11380g	PISO0H11381g
STE13	YOR219C	DEHA0G24486g	DEHA2G23144g	PISO0I16002g	PISO0J17807g
STE23	YLR389C	DEHA0A05236g	DEHA2A05214g	PISO0D03589g	PISO0C03500g
RCE1	YMR274C	DEHA0F08041g	DEHA2F07260g	PISO0K06126g	PISO0L06127g
STE24	YJR117W	DEHA0F06820g	DEHA2F06248g	PISO0I14264g	PISO0J16069g
RAM1	YDL090C	DEHA0G18007g	DEHA2G16918g	PISO0G06122g	PISO0H06123g
STE14	YDR410C	DEHA0G06017g	DEHA2G05368g	PISO0C09066g	PISO0D09133g
STE6	YKL209C	DEHA0F18667g	DEHA2F17226g	PISO0I06388g	PISO0J08193g
STE2	YFL026W	DEHA0A11110g	DEHA2A10736g	PISO0F04823g	PISO0E03414g
STE3	YKL178C	DEHA0D04708g	DEHA2D04004g	PISO0I04254g	PISO0J06059g
GPA1	YHR005C	DEHA0D14894g	DEHA2D13618g	PISO0K01594g	PISO0L01595g
SST2	YLR452C	DEHA0E10692g	DEHA2E10164g	PISO0K11252g	PISO0L11253g
STE4	YOR212W	DEHA0C17600g	DEHA2C16368g	PISO0K21680g	PISO0L21681g
STE5	YDR103W	DEHA0G13684g	DEHA2G12826g	PISO0F10873g	PISO0E09508g
STE18	YJR086W	yes	DEHA2G24024g	PISO0I16882g	PISO0J18643g
STE20	YHL007C	DEHA0E23529g	DEHA2E22220g	PISO0F14173g	PISO0E12786g
STE11	YLR362W	DEHA0B04895g	DEHA2B05016g	PISO0A07018g	PISO0B07085g
STE7	YDL159W	DEHA0F15719g	DEHA2F14498g	PISO0I08896g	PISO0J10723g
STE12	YHR084W	DEHA0F27445g	DEHA2F25894g	PISO0N06877g	PISO0M06744g
FAR1	YJL157C	DEHA0F14905g	DEHA2F13728g	PISO0I09622g	PISO0J11427g
STE50	YCL032W	DEHA0D19019g	DEHA2D17490g	PISO0A09240g	PISO0B09307g
DIG1/2	YDR480W	DEHA0D17083g	DEHA2D15686g	PISO0I01196g	PISO0E01368g
CDC24	YAL041W	DEHA0E12452g	DEHA2E11836g	PISO0N16799g	PISO0M16402g
CDC42	YLR229C	DEHA0G15037g	DEHA2G14168g	PISO0F12083g	PISO0E10718g
BEM1	YBR200W	DEHA0E22924g	DEHA2E21670g	PISO0F12523g	PISO0E11136g
FUS3	YBL016W	DEHA0E04290g	DEHA2E03586g	PISO0K16774g	PISO0L16775g
		DEHA0E21219g	DEHA2E20086g	PISO0N15083g	PISO0M14686g
BNI1	YNL271C	DEHA0E14938g	DEHA2E14366g	PISO0N19065g	PISO0M18822g
PFY1	YOR122C	DEHA0G15807g	DEHA2G14784g	PISO0G07794g	PISO0H07795g
ACT1	YFL039C	DEHA0D06193g	DEHA2D05412g	PISO0A06226g	PISO0B06293g
BUD6	YLR319C	DEHA0C12034g	DEHA2C11022g	PISO0G12018g	PISO0H12019g

SPA2	YLL021W	DEHA0E17325g	DEHA2E16588g	PISO0N21199g	PISO0M20956g
PEA2	YER149C	DEHA0G12474g	DEHA2G11792g	PISO0F09883g	PISO0E08540g
AXL1	YPR122W	DEHA0E03179g	DEHA2E02464g	PISO0A01364g	PISO0B01541g
BIM1	YER016W	DEHA0D15554g	DEHA2D14278g	PISO0N01619g	PISO0M01464g
HO	YDL227C	No	No	No	No
KAR1	YNL188W	No	No	No	No
KAR2	YJL034W	DEHA0A01749g	DEHA2A01364g	PISO0F00489g	PISO0J00471g
KAR3	YPR141C	DEHA0B02068g	DEHA2B02068g	PISO0N23795g	PISO0M23530g
KAR4	YCL055W	DEHA0E24156g	DEHA2E22770g	PISO0N08659g	PISO0M08482g
KAR5	YMR065W	DEHA0E22396g	DEHA2E21208g	PISO0F12963g	PISO0E11576g
KAR9	YPL269W	DEHA0D05797g	DEHA2D05016g	PISO0A06578g	PISO0B06645g
**Meiosis**					
CDC27	YBL084C	DEHA0F21934g	DEHA2F20614g	PISO0G10456g	PISO0H10457g
APC4	YDR118W	DEHA0B08327g	DEHA2B08316g	PISO0G03702g	PISO0H03703g
CDC16	YKL022C	DEHA0C07876g	DEHA2C07062g	PISO0I11602g	PISO0J13429g
APC1	YNL172W	DEHA0D09867g	DEHA2D08932g	PISO0K05862g	PISO0L05863g
APC5	YOR249C	DEHA0C09548g	DEHA2C08668g	PISO0N09099g	PISO0M08900g
CDC23	YHR166C	DEHA0E02805g	DEHA2E02156g	PISO0A01122g	PISO0B01277g
CDC26	YFR036W	No	No	No	No
CDH1	YGL003C	DEHA0A06963g	DEHA2A06864g	PISO0C05018g	PISO0D05107g
CDC20	YGL116W	DEHA0E22132g	DEHA2E20966g	PISO0F13205g	PISO0E11818g
SWM1	YDR260C	DEHA0G13827g	DEHA2G12958g	PISO0F10983g	PISO0E09618g
CDC10	YCR002C	DEHA0F18557g	DEHA2F17116g	PISO0I06476g	PISO0J08303g
CDC3	YLR314C	DEHA0D13794g	DEHA2D12540g	PISO0K02518g	PISO0L02519g
CDC14	YFR028C	DEHA0F16632g	DEHA2F15356g	PISO0I07972g	PISO0J09799g
CDC5	YMR001C	DEHA0F10923g	DEHA2F09966g	PISO0K08832g	PISO0L08833g
CLB1/2 (WGD gene pair)		DEHA0A02189g	DEHA2A01760g	PISO0F00797g	PISO0J00779g
CLB3/4 (WGD gene pair)		DEHA0G25520g	DEHA2G24134g	PISO0I17014g	PISO0J18775g
CLB5/6 (WGD gene pair)		No	No	No	No
IME1	YJR094C	No	No	No	No
IME2	YJL106W	DEHA0B00770g	DEHA2B00748g	PISO0N24499g	PISO0M24212g
IME4	YGL192W	DEHA0B04491	DEHA2B04598g	PISO0A07392g	PISO0B07459g
IDS2	YJL146W	No	No	No	No
RIM4	YHL024W	DEHA0F03850g	DEHA2F03476g	PISO0A10582g	PISO0B10649g
RME1	YGR044C	DEHA0F21098g	DEHA2F19778g	PISO0G09554g	PISO0H09555g
NDT80	YHR124W	DEHA0A07392g	DEHA2A07282g	PISO0C05414g	PISO0D05503g
		DEHA0F22594g	DEHA2F21230g	PISO0N04215g	PISO0M04082g
SUM1	YDR310C	No	No	No	No
RIM11	YMR139W	DEHA0F09647g	DEHA2F08756g	PISO0K07556g	PISO0L07557g
RIM13	YMR154C	DEHA0G22297g	DEHA2G20988g	PISO0A02486g	PISO0B02597g
RIM15	YFL033C	DEHA0F24013g	DEHA2F22572g	PISO0N02983g	PISO0M02850g
RIM101	YHL027W	DEHA0D05577g	DEHA2D04796g	PISO0F01589g	PISO0J01571g

UME3	YNL025C	DEHA0A10131g	DEHA2A09878g	PISO0F05615g	PISO0E04206g
UME6	YDR207C	DEHA0G16236g	DEHA2G15202g	PISO0N20869g	PISO0M20626g
		DEHA0E16929g	DEHA2E16236g	PISO0N20869g	PISO0M20626g
MCK1	YNL307C	DEHA0E05819g	DEHA2E05060g	PISO0K15630g	PISO0L15631g
SPO12/BNS1 (WGD gene pair)	YHR152W	No	No	No	No
SPO11	YPR007C	DEHA0B13156g	DEHA2B13112g	PISO0D02621g	PISO0C02554g
SPO13	YHR014W	No	No	No	No
SPO22	YIL073C	No	No	No	No
REC102	YLR329W	DEHA0A06699g	DEHA2A06622g	PISO0C04754g	PISO0D04865g
MRE11	YMR224C	DEHA0F17116g	DEHA2F15818g	PISO0I07598g	PISO0J09425g
RAD50	YNL250W	DEHA0D18799g	DEHA2D17314g	PISO0A09042g	PISO0B09109g
RAD1	YPL022W	DEHA0F09823g	DEHA2F08932g	PISO0K07710g	PISO0L07711g
MUS81	YDR386W	DEHA0G05852g	DEHA2G05214g	PISO0C09220g	PISO0D09287g
MER3	YGL251C	DEHA0F08877g	DEHA2F07986g	PISO0A01892g	PISO0B02069g
RAD54	YGL163C	DEHA0D06248g	DEHA2D05456g	PISO0A06182g	PISO0B06249g
MLH1	YMR167W	DEHA0G23320g	DEHA2G22022g	PISO0A03410g	PISO0B03565g
MLH2	YLR035C	No	No	No	No
MLH3	YPL164C	DEHA0E11066g	DEHA2E10472g	PISO0F15295g	PISO0E13908g
PMS1	YNL082W	DEHA0A11297g	DEHA2A10868g	PISO0F04713g	PISO0E03304g
REC8	YPR007C	DEHA0D07843g	DEHA2D07106g	PISO0A04884g	PISO0B04973g
REC114	YMR133W	No	No	No	No
CDC31	YOR257W	DEHA0F16082g	DEHA2F14784g	PISO0I08654g	PISO0J10481g
CSM1	YCR086W	DEHA0A13904g	DEHA2A13486g	PISO0A11132g	PISO0B11199g
CSM3	YMR048W	DEHA0F05522g	DEHA2F05038g	PISO0I13120g	PISO0J14925g
SMC1	YFL008W	DEHA0F21296g	DEHA2F20020g	PISO0G09884g	PISO0H09885g
SMC2	YFR031C	DEHA0B15268g	DEHA2B15136g	PISO0D01059g	PISO0C00970g
SMC3	YJL074C	DEHA0G01958g	DEHA2G01606g	PISO0N02037g	PISO0M01904g
SMC4	YLR086W	No	**DEHA2B07920g**	**PISO0N13059g**	**PISO0M12750g**
SMC5	YOL034W	No	**DEHA2A12606g**	**PISO0F03261g**	**PISO0J03221g**
SMC6	YLR383W	DEHA0A05346g	DEHA2A05324g	PISO0D03721g	PISO0C03632g
SCC1	YDL003W	DEHA0A14476g	DEHA2A14058g	PISO0N22189g	PISO0M21946g
SCC3	YIL026C	DEHA0D05203g	DEHA2D04488g	PISO0F01303g	PISO0J01285g
PDS5	YMR076C	DEHA0F20966g	DEHA2F19646g	PISO0G09444g	PISO0H09445g
RAD51	YER095W	DEHA0C17952g	DEHA2C16698g	PISO0K22098g	PISO0L22099g
RAD52	YML032C	DEHA0G05786g	DEHA2G05148g	PISO0C09264g	PISO0D09331g
RAD55	YDR076W	No	No	No	No
RAD57	YDR004W	DEHA0B13068g	DEHA2B13024g	PISO0D02687g	PISO0C02620g
REC104	YHR157W	No	No	No	No
DMC1	YER179W	DEHA0E17479g	DEHA2E16742g	PISO0N21353g	PISO0M21110g
MND1	YGL183C	DEHA0E04994g	DEHA2E04246g	PISO0K16290g	PISO0L16291g
HOP2	YGL033W	DEHA0D13442g	DEHA2D12210g	PISO0K02804g	PISO0L02805g
MMS4	YBR098W	DEHA0F16434g	DEHA2F15158g	PISO0I08280g	PISO0J10107g

MSH4	YFL003C	No	No	No	No
MSH5	YDL154W	No	No	No	No
MSH2	YOL090W	DEHA0B16005g	DEHA2B15818g	PISO0D00421g	PISO0C00332g
MSH6	YDR097C	DEHA0E16566g	yes	PISO0N20495g	PISO0M20252g
SAE2	YGL175C	DEHA0C15290g	DEHA2C14212g	PISO0G14900g	PISO0H14901g
MEI5	YPL121C	DEHA0G24640g	DEHA2G23298g	PISO0I16156g	PISO0J17961g
SAE3	YHR079C-A	No	No	No	No
HOP1	YIL072W	DEHA0D16060g	DEHA2D14740g	PISO0N01245g	PISO0M01090g
RED1	YLR263W	No	No	No	No
MEK1	YOR351C	DEHA0F24684g	DEHA2F23254g	PISO0G15362g	PISO0H15363g
ZIP1	YDR285W	No	No	No	No
ZIP2	YGL249W	No	No	No	No
MND2	YIR025W	DEHA0F21802g	DEHA2F20482g	PISO0G10346g	PISO0H10347g
MAM1	YER106W	No	No	No	No
ZIP3	YLR394W	No	No	No	No
STU1	YBL034C	DEHA0F27423g	DEHA2F25872g	PISO0N06855g	PISO0M06722g
TID3	YIL144W	DEHA0E10065g	DEHA2E09548g	PISO0K11824g	PISO0L11825g
UBC11	YOR339C	No	No	No	No
RAD23	YEL037C	DEHA0B04092g	DEHA2B04180g	PISO0A07810g	PISO0B07877g
EXO1	YOR033C	DEHA0E16071g	DEHA2E15444g	PISO0N20055g	PISO0M19812g
HRR25	YPL204W	DEHA0C04884g	DEHA2C04290g	PISO0K19392g	PISO0L19393g
HUL4	YJR036C	DEHA0F12749g	DEHA2F11704g	PISO0K10592g	PISO0L10593g
LEE1	YPL054W	DEHA0D17303g	DEHA2D15928g	PISO0I00976g	PISO0E01148g
ENA2	YDR039C	DEHA0G09878g	DEHA2G09108g	PISO0C05656g	PISO0D05745g
PMC1	YGL006W	DEHA0A09295g	DEHA2A09086g	PISO0F06473g	PISO0E05152g
CMK2	YOL016C	DEHA0F10197g	DEHA2F09284g	PISO0K08172g	PISO0L08173g
CHS1	YNL192W	DEHA0D04620g	DEHA2D03916g	PISO0I04144g	PISO0J05949g
ISA1	YLL027W	DEHA0A06787g	DEHA2A06710g	PISO0C04842g	PISO0D04953g
HTZ1	YOL012C	DEHA0E06501g	DEHA2E05720g	PISO0K14992g	PISO0L14993g
ATG8	YBL078C	DEHA0D04664g	DEHA2D03960g	PISO0I04210g	PISO0J06015g
BAG7/SAC7 (WGD)	YOR134W	DEHA0G12881g	DEHA2G12122g	PISO0F10191g	PISO0E08848g
ROM2	YLR371W	DEHA0G06094g	DEHA2G05456g	PISO0C08978g	PISO0D09045g
RAS2	YNL098C	DEHA0F10351g	DEHA2F09438g	PISO0K08348g	PISO0L08349g
GNA1	YFL017C	DEHA0B05005g	DEHA2B05126g	PISO0A06974g	PISO0B07041g
SGA1	YIL099W	DEHA0A12705g	DEHA2A12254g	PISO0F03459g	PISO0J03419g
CLG1	YGL215W	DEHA0C15642g	DEHA2B03630g	PISO0A08250g	PISO0B08317g
CYB2	YML054C	DEHA0D06325g	DEHA2D05522g	PISO0A06116g	PISO0B06183g
ECM4	YKR076W	DEHA0C17798g	DEHA2C16566g	PISO0K21922g	PISO0L21923g
TOS7	YOL019W	DEHA0G09702g	DEHA2G08932g	PISO0C05832g	PISO0D05899g

ARN2	YHL047C	No	No	No	No
GTT1	YIR038C	DEHA0D17677g	DEHA2D16280g	PISO0I00624g	PISO0E00796g
RIB5	YBR256C	DEHA0D15202g	DEHA2D13926g	PISO0N01861g	PISO0M01706g
CHO1	YER026C	DEHA0B15851g	DEHA2B15686g	PISO0D00553g	PISO0C00464g
XKS1	YGR194C	DEHA0C07788g	DEHA2C06974g	PISO0I11690g	PISO0J13517g
PCT1	YGR202C	DEHA0G22858g	DEHA2G21560g	PISO0A03036g	PISO0B03169g
ELC1	YPL046C	DEHA0D14322g	DEHA2D13046g	PISO0K02144g	PISO0L02145g
SYF2	YGR129W	DEHA0C03894g	DEHA2C03366g	PISO0B10099g	PISO0A10032g
PGM2	YMR105C	DEHA0C05940g	DEHA2C05258g	PISO0K18534g	PISO0L18535g
RKI1	YOR095C	DEHA0A09537g	DEHA2A09328g	PISO0F06231g	PISO0E04910g
SUR4	YLR372W	DEHA0G06116g	DEHA2G05478g	PISO0C08956g	PISO0D09023g
PIB1	YDR313C	DEHA0E08569g	DEHA2E08030g	PISO0K13144g	PISO0L13145g
PIN3	YPR154W	DEHA0B14575g	DEHA2B14476g	PISO0D01609g	PISO0C01520g
SSO2	YMR183C	DEHA0B09790g	DEHA2B09570g	PISO0G02668g	PISO0H02669g
FBP1	YLR377C	DEHA0F01309g	DEHA2F01100g	PISO0C12652g	PISO0D12719g
GLG1/GLG2 (WGD)	YKR058W	DEHA0F18579g	DEHA2F17138g	PISO0N00959g	PISO0M00804g
ARE2	YNR019W	DEHA0F25652g	DEHA2F24222g	PISO0A11748g	PISO0B11815g
GDI1	YER136W	DEHA0F26620g	DEHA2F25102g	PISO0N06129g	PISO0M05974g
PDC1	YLR044C	DEHA0B03784g	DEHA2B03872g	PISO0A08052g	PISO0B08119g
OXR1	YPL196W	DEHA0G01661g	DEHA2G01320g	PISO0K01242g	PISO0L01243g
KGD1	YIL125W	DEHA0F19217g	DEHA2F17798g	PISO0N14709g	PISO0M14312g
DAP1	YPL170W	DEHA0F16170g	DEHA2F14872g	PISO0I08566g	PISO0J10393g
SPO7	YAL009W	DEHA0C17336g	DEHA2C16104g	PISO0K21416g	PISO0L21417g
GSG1	YDR108W	DEHA0E17578g	DEHA2E16830g	PISO0N21419g	PISO0M21176g
RMD5	YDR255C	DEHA0D14300g	DEHA2D13024g	PISO0K02166g	PISO0L02167g
EMI1	YDR512C	DEHA0C07315g	DEHA2C06512g	PISO0K17390g	PISO0L17391g
RMD8	YFR048W	DEHA0F24420g	DEHA2F22990g	PISO0N02587g	PISO0M02454g
RMD11	YHL023C	DEHA0E02695g	DEHA2E02046g	PISO0A01034g	PISO0B01189g
NEM1	YHR004C	DEHA0B03003g	DEHA2B02970g	PISO0N23113g	PISO0M22848g
SPO16	YHR153C	No	No	No	No
EMI5	YOL071W	DEHA0E14311g	DEHA2E13750g	PISO0N18537g	PISO0M18294g
MUM2	YBR057C	DEHA0G13068g	DEHA2G12298g	PISO0F10367g	PISO0E09024g
MUS81	YDR386W	DEHA0G05852g	DEHA2G05214g	PISO0C09220g	PISO0D09287g
NAM8	YHR086W	DEHA0F24706g	DEHA2F23276g	PISO0G15384g	PISO0H15385g
PFS1	YHR185C	DEHA0G22770g	DEHA2G21472g	PISO0A02948g	PISO0B03081g
ADY3/CNM67 (WGD)	YDL239C	No	No	No	No
ADY4	YLR227C	Yes	No	No	No
AMA1	YGR225W	DEHA0B14591g	DEHA2B14498g	PISO0D01587g	PISO0C01498g
CDA1/CDA2 (Gene duplication pair)	YLR307W	DEHA0C12815g	DEHA2C11836g	PISO0G12612g	PISO0H12613g
CRR1	YLR213C	No	No	No	No
DIT1	YDR403W	No	No	PISO0A12408g	PISO0B12475g

DIT2	YDR402C	No	No	PISO0A12386g	PISO0B12453g
DON1/CUE5 (WGD)	YDR273W	DEHA0E07755g	DEHA2E07260g	PISO0K13738g	PISO0L13739g
DTR1	YBR180W	DEHA0A10670g	DEHA2A10362g	PISO0I02736g	PISO0J04519g
ISC10	YER180C	No	No	No	No
MNN4	YKL201C	DEHA0B04675g	DEHA2B04796g	PISO0A07172g	PISO0B07239g
MPC54	YOR177C	No	No	No	No
MSO1	YNR049C	DEHA0F11682g	DEHA2F10692g	PISO0K09602g	PISO0L09603g
MUM3	YOR298W	DEHA0D18832g	DEHA2D17336g	PISO0A09064g	PISO0B09131g
NDT80	YHR124W	DEHA0A07392g	DEHA2A07282g	PISO0C05414g	PISO0D05503g
OSW1	YOR255W	No	No	No	No
OSW2	YLR054C	No	No	No	No
SMA1	YPL027W	No	No	No	No
SMA2	YML066C	Yes	**DEHA2F06072g**	**PISO0I14088g**	**PISO0J15893g**
SMK1	YPR054W	Yes	DEHA2E18348g	PISO0N05799g	PISO0M05644g
SPO14	YKR031C	DEHA0C03311g	DEHA2C02926g	PISO0I18224g	PISO0J19985g
SPO20	YMR017W	No	No	No	No
SPO21/YSW1 (WGD)	YOL091W	No	No	No	No
SPO71	YDR104C	DEHA0G13662g	DEHA2G12804g	PISO0F10851g	PISO0E09486g
SPO73	YER046W	DEHA0A13761g	DEHA2A13332g	PISO0A10956g	PISO0B11023g
SPO74	YGL170C	No	No	No	No
SPO75	YLL005C	DEHA0G18480g	DEHA2G17292g	PISO0G05550g	PISO0H05551g
SPO77	YLR341W	No	No	No	No
SPS1	YDR523C	DEHA0E16115g	DEHA2E15488g	PISO0N20099g	PISO0M19856g
SPS4	YOR313C	DEHA0G07007g	DEHA2G06336g	PISO0C08032g	PISO0D08099g
SSP1	YHR184W	DEHA0E08371g	DEHA2E07854g	PISO0K13342g	PISO0L13343g
SSP2	YOR242C	DEHA0C13277g	DEHA2C12320g	PISO0G13206g	PISO0H13207g
TEP1	YNL128w	DEHA0B05984g	DEHA2B06116g	PISO0N11497g	PISO0M11188g
YAL018C	YAL018c	No	DEHA2C15598g	PISO0K20954g	PISO0L20955g
YEL023C	YEL023c	No	No	PISO0K10636g	PISO0L10637g
SPO1	YNL012W	DEHA0F25861g	DEHA2F24420g	PISO0A11946g	PISO0B12013g
Spombe mug66	No	DEHA0B08591g	DEHA2B08580g	PISO0G03460g	PISO0H03461g

¥ *S. cerevisiae* and *D. hansenii* (version_1) genes were extracted from the supplementary tables 34 and 35 from Butler et al. 2009. *D hansenii* genome : * version_1 from Dujon et al. (2004), ** version_2 (open access at http://www.genolevures.org/deha.html#)The Génolevures Consortium (Souciet *et al*., 2009), In bold, orthologs in *D. hansenii* version_2; in grey, orthologs in *P. sorbitophila* absent in *D. hansenii* version_2
