# Supplementary material for: Pichia sorbitophila, an Interspecies Yeast Hybrid, Reveals Early Steps of Genome Resolution After Polyploidization
Source: G3 (Bethesda). 2012 Feb 1;2(2):299–311. doi: 10.1534/g3.111.000745 (PMC3284337; doi:10.1534/g3.111.000745)
Supplement: Supporting Information [file supp_2.2.299_TableS7.pdf]

Table S7 Comparison of *P. sorbitophila* global genome features with other yeasts

	ploidy	Genome size (Mb)	Number of chromosomes	average GC content (%)	total CDS	average gene density (%)&	GC in cds (%)	average CDS size&
P. sorbitophila	*hybrid*	21.5	14	41.4	11,252	76.9 (74.5-78.1%)	42	1,476
P. Sorbitophila	*eq. haploid $*	10.75	7	-	5,736	-	-	-
D. hansenii	*haploid*	12.2	7	36.3	6,395	74.2	38.0	1,440
C. guilliermondii	*haploid*	10.6	8	43.8	5,920	-	-	1,402
P. stipitis	*haploid*	15.4	8	41.1	5,841	-	-	-
C. albicans	*diploid*	14.3*	8	33.4	6,107	-	-	1,468
C. tropicalis	*diploid*	14.5*	8	33.1	6,258	-	-	1,454
L. elongisporus	*diploid*	15.4*	8	37.0	5,802	-	-	1,530
C. parapsilosis	*diploid*	13.1*	8	38.7	5,733	-	-	1,533
C. lusitaniae	*haploid*	12.1	8	44.5	5,941	-	-	1,382
Y. lipolytica	*haploid*	20.5	6	49.0	6,580	46.0	53.8	1,470
S. cerevisiae £	*haploid*	12.1	16	38.3	5,769	70.0	40.3	1,467

Genome features of *P. sorbitophila* were compared with other yeasts of the CTG group, with *Yarrowia lipolytica* (*Dipodascaceae* group) and *Saccharomyces cerevisiae* (*Saccharomycetaceae* group). & Average gene density and size is the mean of the values obtained for each chromosome. $ eq. Equivalent in ploidy, total CDS corresponds to identified genes (Supporting Section 2). * Sizes of the haploid genomes given in published data (Table S5 for references) £ The Génolevures Consortium (Souciet *et al.*, 2009)
