# Supplementary material for: Pichia sorbitophila, an Interspecies Yeast Hybrid, Reveals Early Steps of Genome Resolution After Polyploidization
Source: G3 (Bethesda). 2012 Feb 1;2(2):299–311. doi: 10.1534/g3.111.000745 (PMC3284337; doi:10.1534/g3.111.000745)
Supplement: Supporting Information [file supp_2.2.299_000745SI.zip › 000745SI/000745SI.pdf]

Table of Contents

I. Supporting Data and Figures

1. Structure of the *Pichia sorbitophila* nuclear genome
- FIGURE S1. Sequencing coverage (X) along scaffolds
- FIGURE S2. Pulse-field gel electrophoresis profile of *Pichia Sorbitophila* chromosomes
- FIGURE S3. Nucleotide sequence identity along chromosomes
- FIGURE S4. Proposed positions of centromeres
- FIGURE S5. Bias in codon usage between Pγ and Pε subgenomes

2. Protein coding genes
- FIGURE S6. Flowchart for the prediction and the annotation of each chromosomal feature in *P. sorbitophila* genome
- FIGURE S7. Splicing pattern determined for spliceosomal introns detected in *P. sorbitophila* genome
- FIGURE S8. Flowchart for gene analyses and comparisons
- FIGURE S9. Distribution of dN/dS values according to the protein sequence identity

3. Ribosomal DNA
- FIGURE S10. Comparison of 5S rDNA sequences
- FIGURE S11. Synteny conservation around the rDNA clusters located on the left arm of *P. sorbitophila* chr E and F

4. Mitochondrial genome

5. tRNA and co-transcribed tRNA, tRNA gene usage

6. Other noncoding RNA genes

7. Synteny breaks between Pg and Pe subgenomes
- FIGURE S12. Comparison of synteny maps at the E/F/I/J reciprocal translocation between CTG yeasts
- FIGURE S13. Gene location movement between two subtelomeric regions
- FIGURE S14. Comparison of synteny maps at single allele gene positions between CTG yeasts

8. Mating type
- FIGURE S15. Comparative organization of the MTL loci in *P. sorbitophila*, *D. hansenii* and *P. stipitis*

References

II. Supporting Tables

- Table S1. Heterozygous and homozygous parts of *P. sorbitophila* genome
- Table S2. Sequence polymorphism in homozygous regions of the *P. sorbitophila* genome
- Table S3. NUMTs in the nuclear genome of *Pichia sorbitophila*
- Table S4. Sequence identity between *P. farinosa CBS 2001* and *P. sorbitophila* subgenomes
- Table S5. Databases for yeasts species used in this study
- Table S6. Distribution of introns in protein-coding genes
- Table S7. Comparison of *P. sorbitophila* global genome features with other yeasts
- Table S8. Characteristics of tandemly duplicated genes arrays
- Table S9. Gene ontology categories for conserved alleles
- Table S10. Hypervariable alleles in heterozygous regions
- Table S11. Gene ontology categories for hypervariable alleles
- Table S12. Gene ontology categories for pseudogenes
- Table S13. Gene ontology categories for single allele genes
- Table S14. tDNA numbers per chromosome in *P. sorbitophila*
- Table S15. Pairs of potentially co-transcribed tRNA genes in *P. sorbitophila*
- Table S16. Codon and tRNA gene usages in *P. sorbitophila* and *D. hansenii*
- Table S17. List of ncRNA genes
- Table S18. *P. sorbitophila* genes in osmotic stress
- Table S19. Conservation of mating and meiosis genes

1. Structure of the *Pichia sorbitophila* nuclear genome

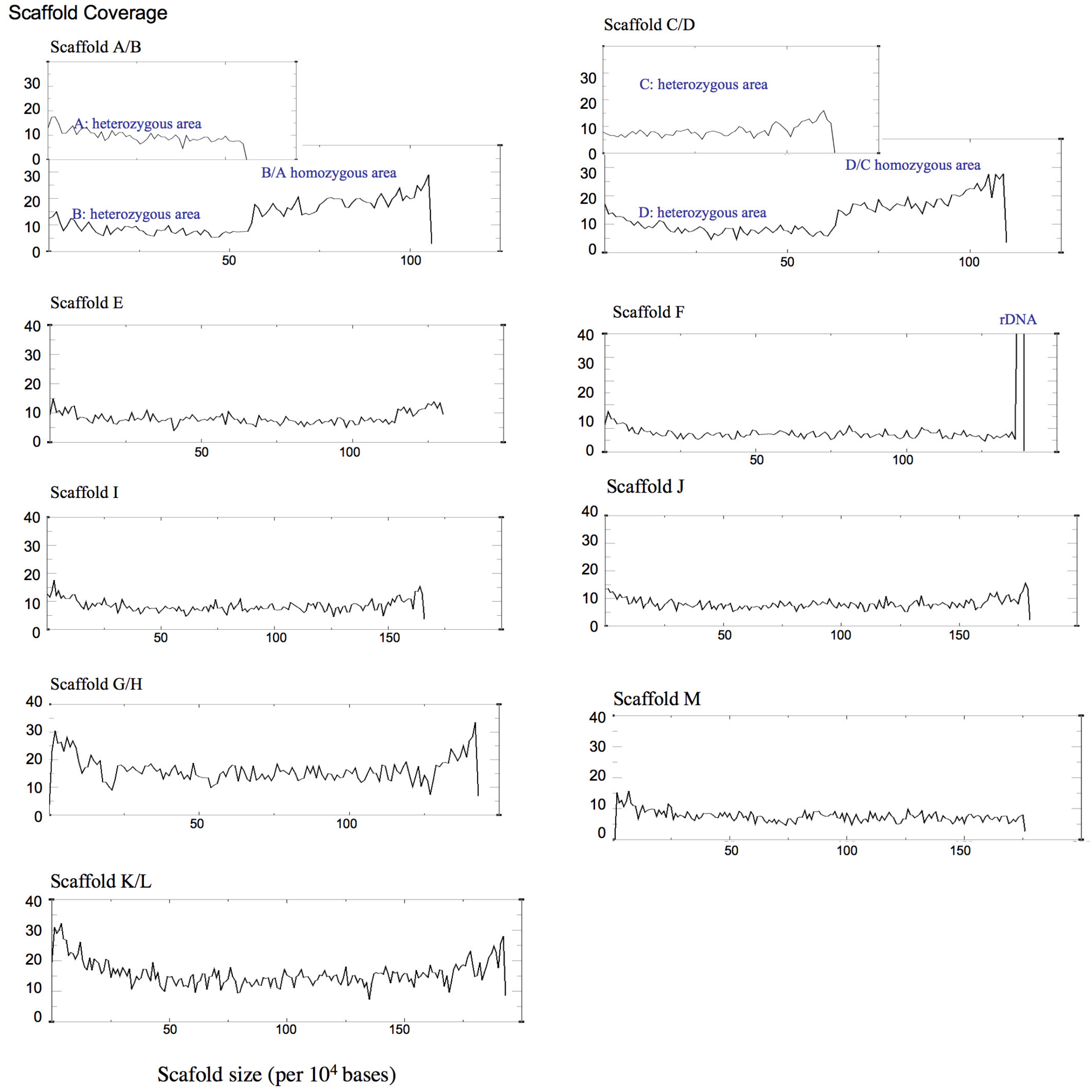

Figure S1 Sequencing coverage (X) along scaffolds. Sequencing depth (Y-axe) was calculated along finished scaffolds (X-axe) by mapping initial Sanger reads on scaffolds. The 11 contigs (7.5X covered) obtained before finishing (see Methods) correspond to the heterozygous regions in A, B, C, D, and to the heterozygous chromosomes E, F, I, J, M, and N (2 contigs), respectively. The six 14X covered contigs correspond to the homozygous parts in A/B, and C/D and to the homozygous chromosomes G/H (2 contigs) and K/L (2 contigs).

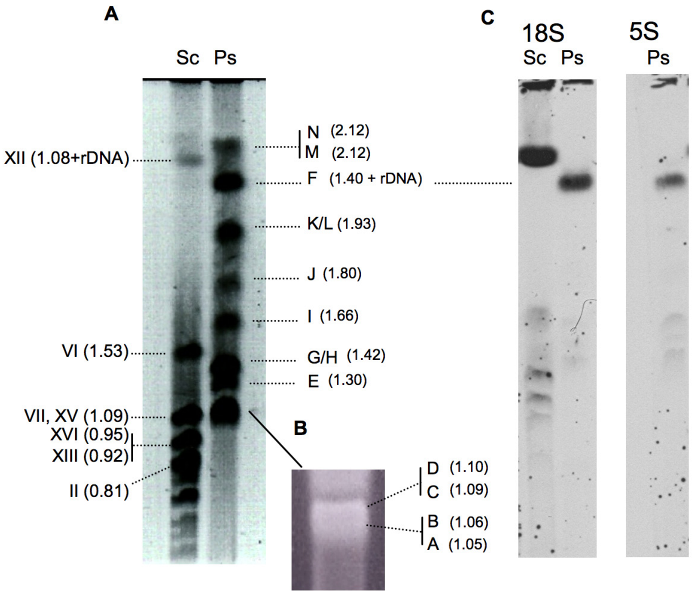

Figure S2 Pulse-field gel electrophoresis profile of *Pichia sorbitophila* chromosomes. Chromosomal DNA was prepared as described previously by Vézinhet *et al.* (1990). Pulsed-field gel electrophoreses were performed using a CHEF Mapper™ pulsed-field electrophoresis system (Bio-Rad), in 1% pulsed field certified agarose (BioRad) and 0,5xTBE (45 mM Tris-borate, 1 mM EDTA). (A) Separation of *P. sorbitophila* (*Ps*) and *S. cerevisiae* (*Sc*) chromosomes with a linear gradient (run time 60h, switch time 250s, angle 106°, voltage gradient 4V/cm) reveals eight bands for *P. sorbitophila*. Name and size (in Mb) of chromosomes (for *Sc*) or scaffolds (for *Ps*) are mapped at the left and the right for *Sc* and *Ps*, respectively. Band intensities are in accordance with the obtained number of *Ps* contigs of equivalent size and which are typically associated in pair: for example, the G and H contigs, identical in size and sequence, correspond to a pair of homozygous chromosomes that comigrate. By comparison, I and J contigs correspond to an heterozygous pair of chromosomes differing in size. (B) A/B and C/D pairs were separated using the following migration conditions: run time 48h, switch time 400s, angle 106°, voltage gradient 3V/cm. (C) Hybridization of chromosomes after transfer onto Hybond N+ membrane (MP biomedicals) and using 5S and 18S rDNA probes amplified from *Ps* and *Sc* genomes. Results show that rDNA repeats are only detected on contig F and confirm the hemizygous state of the rDNA locus.

Additional Note : The size dissimilarity observed between chr. E and F, around 440 kb, is widely due to the absence of an equivalent rDNA repeated cluster on chr. E. This explains why previous hybridization data obtained by Suzuky *et al*. (2003) on *P. sorbitophila* CBS 7064 chromosomes, showed two bands at 1.37 and 1.90 Mb respectively, using a *URA3*-specific probe. They detected therefore both alleles of the gene located respectively on chr. E and F. One can notice that both *URA3* allelic sequences previously published (accession numbers AB109042 and AB109043) are identical to the ones obtained in this study.

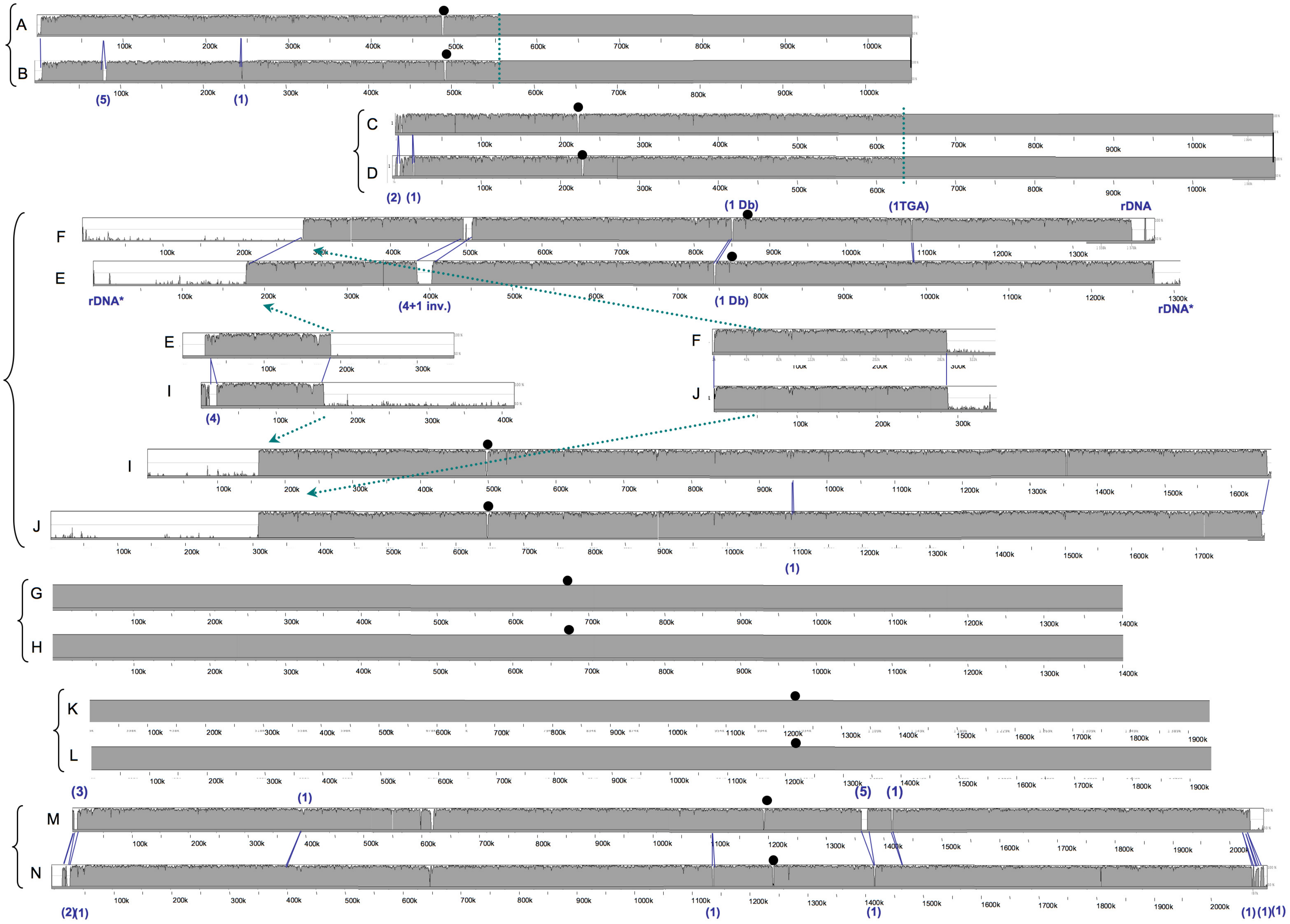
A
B
(5)
(1)
C
D
(2) (1)
(1 Db)
(1TGA)
rDNA
F
E
rDNA*
(4+1 inv.)
(1 Db)
rDNA*
E
I
(4)
F
J
I
J
(1)
G
H
K
L
(3)
(1)
(5)
(1)
M
N
(2)(1)
(1)
(1)
(1)(1)(1)

Figure S3 Nucleotide sequence identity along chromosomes. Pairs of chromosomes were aligned with LAGAN (Brudno *et al.*, 2003) using the global pairwise alignment option and default parameters. Variations of identity (from 50% to 100%) were visualized with the graphical option provided by LAGAN. Switch of identity between chromosomes E/F/I/J, or transition between heterozygous and homozygous regions in A/B and C/D are indicated by green arrows and lines. Positions of rDNA clusters, degenerated (*) or not, are also indicated. Identity and gap percentages were recomputed from the global alignments using a sliding window of 5kb and a step of 1kb to define positions of identity drops (< 70% identity). These positions were then manually curated for the presence of protein coding genes or other genomic elements. Areas of identity drop range from 2.2 kb to 18.5 kb. They correspond either to putative centromere positions (•), sequence variations in intergenic regions or local synteny losses. In the latter cases (14 cases) the number of non syntenic genes is indicated in brackets below the concerned chromosomes. 39 protein-coding genes are concerned, out of which 16 genes are closed to the chromosome ends and can be associated to the 67 other genes located in subtelomeric regions. The remaining 23 genes are located in internal regions. (TGA). The additional gene identified on chr. F is the result of a tandem gene duplication leading to an array of two genes on chr. F against 1 gene on chr. E. (inv.) inversion, the two allelic genes are not in the same orientation (see also Supp. Fig. 14). (Db) dubious ORF.

A

Proposed positions for centromeres

chromosome	Centromere					global_GC%*	Upstream_region		Downstream_region	
	start	end	length	area	local_GC%		upstream_gene	upstream_locus	dowstream_gene	downstream_locus
Piso0A	483754	486340	2587	he	31.117	41.726	Piso0_000278	PISO0A05830g	Piso0_000279	PISO0A05852g
Piso0B	491886	494328	2443	he	30.372	41.362	Piso0_000278	PISO0B05897g	Piso0_000279	PISO0B05919g
Piso0C	222931	225224	2294	he	28.640	40.458	Piso0_000725	PISO0C02686g	Piso0_000726	PISO0C02708g
Piso0D	230388	232657	2270	he	29.031	40.548	Piso0_000725	PISO0D02753g	Piso0_000726	PISO0D02775g
Piso0E	749453	751963	2511	he	30.506	41.356	Piso0_001590	PISO0E08386g	Piso0_001591	PISO0E08408g
Piso0F	850966	853576	2611	he	30.601	41.741	Piso0_001590	PISO0F09729g	Piso0_001591	PISO0F09751g
Piso0G	660324	663504	3181	hm	30.934	41.547	Piso0_003261	PISO0G08432g	Piso0_003262	PISO0G08454g
Piso0H	660324	663504	3181	hm	30.934	41.547	Piso0_003261	PISO0H08433g	Piso0_003262	PISO0H08455g
Piso0I	501951	504862	2912	he	30.529	41.534	Piso0_002232	PISO0I05728g	Piso0_002233	PISO0I05772g
Piso0J	644011	646788	2778	he	31.965	40.854	Piso0_002232	PISO0J07533g	Piso0_002233	PISO0J07555g
Piso0K	1216294	1218822	2529	hm	30.645	41.983	Piso0_004330	PISO0K14508g	Piso0_004331	PISO0K14530g
Piso0L	1216294	1218822	2529	hm	30.645	41.983	Piso0_004330	PISO0K14508g	Piso0_004331	PISO0K14530g
Piso0M	1227373	1230078	2706	he	29.527	40.791	Piso0_005451	PISO0M14972g	Piso0_005451	PISO0M14994g
Piso0N	1246461	1249237	2777	he	31.761	41.299	Piso0_005451	PISO0N15369g	Piso0_005451	PISO0N15391g

* global GC content is calculated for the full lenght chromosome

B

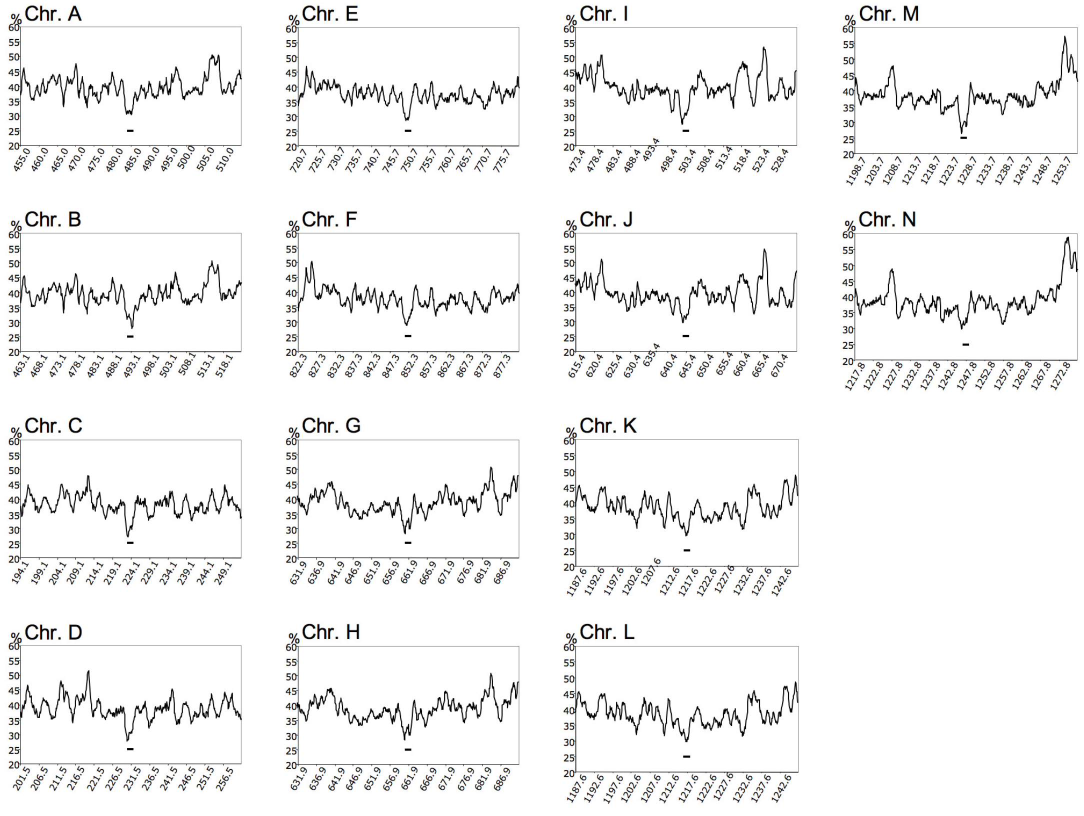

Figure S4 Proposed positions of centromeres. GC content along chromosomes was calculated for sliding windows of 1kb using a step of 0.1kb between successive windows. Windows having a GC content value inferior to two fold the standard deviation value determined for the corresponding chromosome, and located at syntenic position for chromosomes forming a pair were retained. For each chromosome, a unique area, devoid of gene, was obtained. (A) Position of centromeres, GC content and flanking genes. (B) GC content variation in a window size of 60 kb around the centromere. Poor GC area are indicated by lines. X-axe: chromosomal coordinates, Y-axe GC%.

A

Heterozygous regions

amino acid	codon	usage %							
		A Pγ	B_Pε	E_Pε	F_Pγ	I_Pγ	J_Pε	M_Pε	N_Pγ
Phe	TTT	50.25	53.70	52.15	50.50	49.69	53.43	54.02	51.22
	TTC	49.75	46.30	47.85	49.50	50.31	46.57	45.98	48.78
Val	GTT	60.88	61.93	60.29	59.77	59.82	61.14	61.95	59.47
	GTC	39.12	38.07	39.71	40.23	40.18	38.86	38.05	40.53
Ser	TCT	63.58	65.46	65.97	64.59	64.24	65.97	66.54	64.48
	TCC	36.42	34.54	34.03	35.41	35.76	34.03	33.46	35.52
Pro	CCT	66.86	68.31	66.44	65.32	66.49	67.90	68.02	66.54
	CCC	33.14	31.69	33.56	34.68	33.51	32.10	31.98	33.46
Thr	ACT	58.47	61.85	58.80	57.47	57.71	60.64	61.29	58.95
	ACC	41.53	38.15	41.20	42.53	42.29	39.36	38.71	41.05
Ala	GCT	62.81	64.31	61.67	60.23	60.92	64.11	63.42	61.21
	GCC	37.19	35.69	38.33	39.77	39.08	35.89	36.58	38.79
His	CAT	59.18	59.42	58.47	58.10	57.77	60.19	60.48	58.92
	CAC	40.82	40.58	41.53	41.90	42.23	39.81	39.52	41.08
Asn	AAT	53.54	55.35	52.52	52.29	53.01	55.69	55.86	54.24
	AAC	46.46	44.65	47.48	47.71	46.99	44.31	44.14	45.76

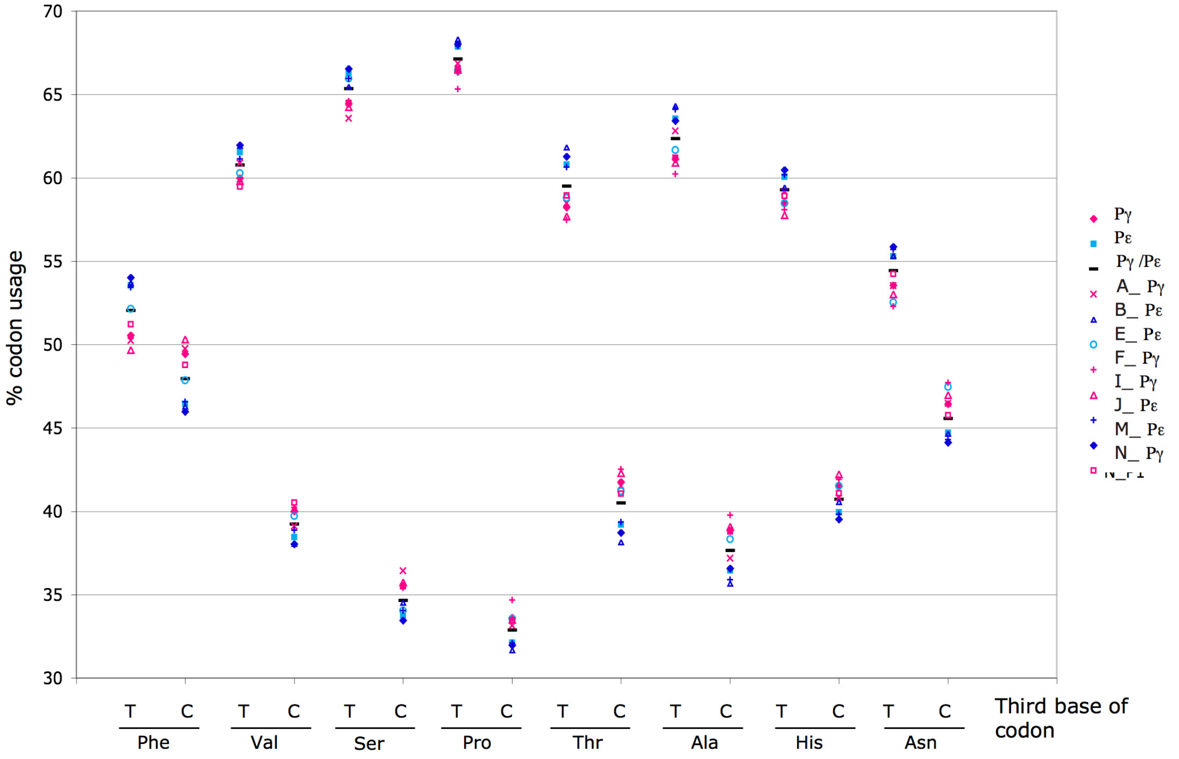

Figure S5 Bias in codon usage between Pγ and Pε subgenomes. For tRNA species that pair with two codons (Crick, 1966), the usage % of each codon was determined as follows: (number of one codon/number of both codonx100). Codon usage % for tRNA species showing more than 1.5 variation between both codons (Table 2) were calculated for each chromosomal region, independently of its origin. The usage % values and their distribution are shown for heterozygous (panel A) and homozygous (panel B) regions. Pγ/Pε corresponds to the mean value between both parents.

B

Homozygous regions

amino acid	codon	usage % A/B	C/D	G/H	K/L
Phe	TTT	50.68	51.12	51.04	49.46
	TTC	49.32	48.88	48.96	50.54
Val	GTT	60.32	61.20	59.10	59.47
	GTC	39.68	38.80	40.90	40.53
Ser	TCT	64.72	65.80	64.17	62.92
	TCC	35.28	34.20	35.83	37.08
Pro	CCT	63.08	68.50	65.07	64.52
	CCC	36.92	31.50	34.93	35.48
Thr	ACT	60.07	59.35	56.09	56.39
	ACC	39.93	40.65	43.91	43.61
Ala	GCT	61.63	63.05	60.87	59.19
	GCC	38.37	36.95	39.13	40.81
His	CAT	56.50	58.84	57.97	55.90
	CAC	43.50	41.16	42.03	44.10
Asn	AAT	51.71	53.93	52.77	52.19
	AAC	48.29	46.07	47.23	47.81

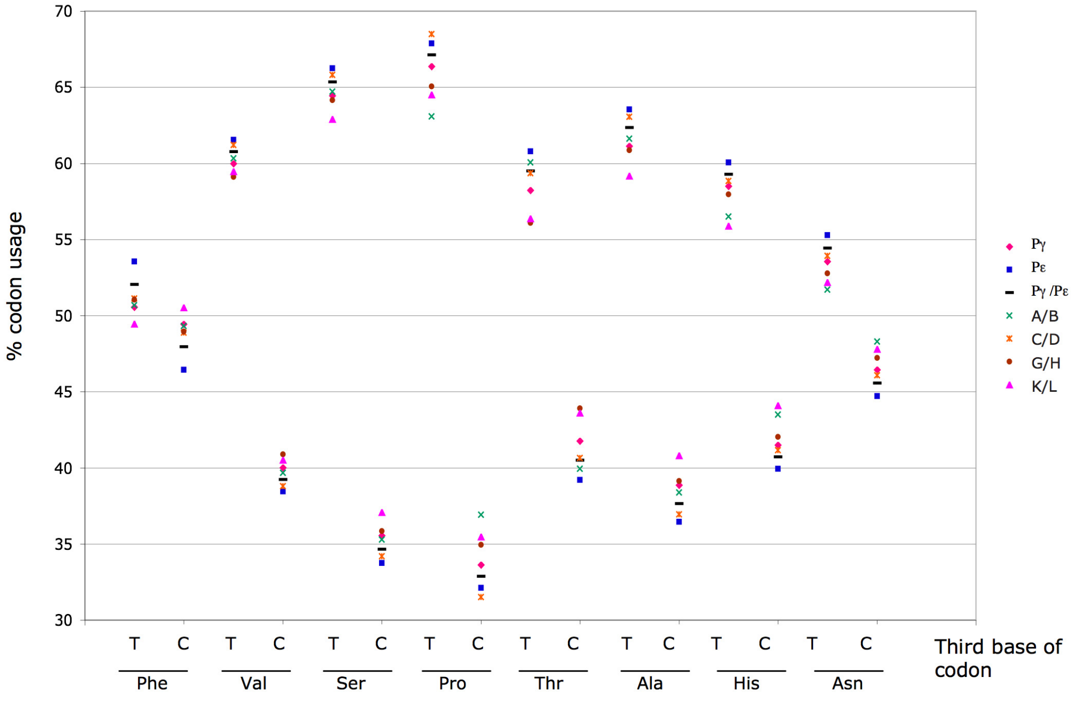

C

Average value of dGC (Fig. 1)

	dGC_Pγ*	dGC_Pε	s**
A/B	1.007	0.993	0.012
C/D_Left$	1.008	0.992	0.012
C/D_Right$	1.005	0.995	0.013
F/JE	1.005	0.995	0.011
I/EJ	1.006	0.994	0.011
M/N	1.006	0.994	0.012
**average**	**1.006**	**0.994**	**0.012**

* Average value of dGC for each heterozygous region.
**Standard deviation for each heterozygous region
$ For the C/D chromosome pair, values refer to regions located on both sides of the reciprocal exchange.

Figure S5 (next). Bias in codon usage between Pγ and Pε subgenomes.

2. Analyses of protein coding genes

Protein coding gene models and spliceosomal introns were predicted and annotated using the same procedure as that used for the protoploid yeast genomes (Souciet *et al*., 2009). Additional steps and annotations were introduced for *P. sorbitophila* because of its hybrid status (Fig. S6). In each chromosomal pair, a protein coding gene is present in most cases in two copies coming either from both parents (in heterozygous regions) or from a sole parent (in homozygous regions). Allelic pairs were determined according to the synteny and homology (Fig. S6). As a result, 5,736 genes were identified in the genome from the 11,252 annotated loci. They are represented by two coding alleles for 5,465 genes, one coding allele and one pseudogene allele for 38 genes, two pseudogene alleles for 13 genes, a single coding allele for 207 genes and a single pseudogene allele for 13 genes. Spliceosomal introns were predicted in 735 gene alleles (Table S6). Most of these alleles contain only one intron (685 alleles) but multi-intronic gene alleles were also detected with up to 4 introns per gene (Table S6) leading to a total of 803 introns. Intron structure is very similar to that of *D. hansenii* (Bon *et al*., 2003) with a mean length of 142 nt and a short distance of about 5 nt between branch point (BP) and 3'-splice site (Fig. S7). Sequences at intron boundaries are highly conserved. The main 5'-splice site (5'ss) motif is GTAWGT with GTAAGT presents in 45.8% of the introns and GTATGT in 42.1% (Fig. S7). The consensus motif for the BP is TACTAAC as in *S. cerevisiae* (Lopez and Séraphin, 1999). In heterozygous regions, we observed that both alleles of a gene (182 genes) contain the same number of introns, almost identical in size. Nucleotide variations in introns between alleles (differences of up to 20 nt in size) correspond to internal insertions, mainly in S1.

Protein-coding genes were sorted out according to their location, the sequence divergence between alleles, the number of paralogs and the associated GO-term, using tools and methods described in Figure S8. Among all, 124 protein-coding alleles were predicted as « dubious » ORFs.

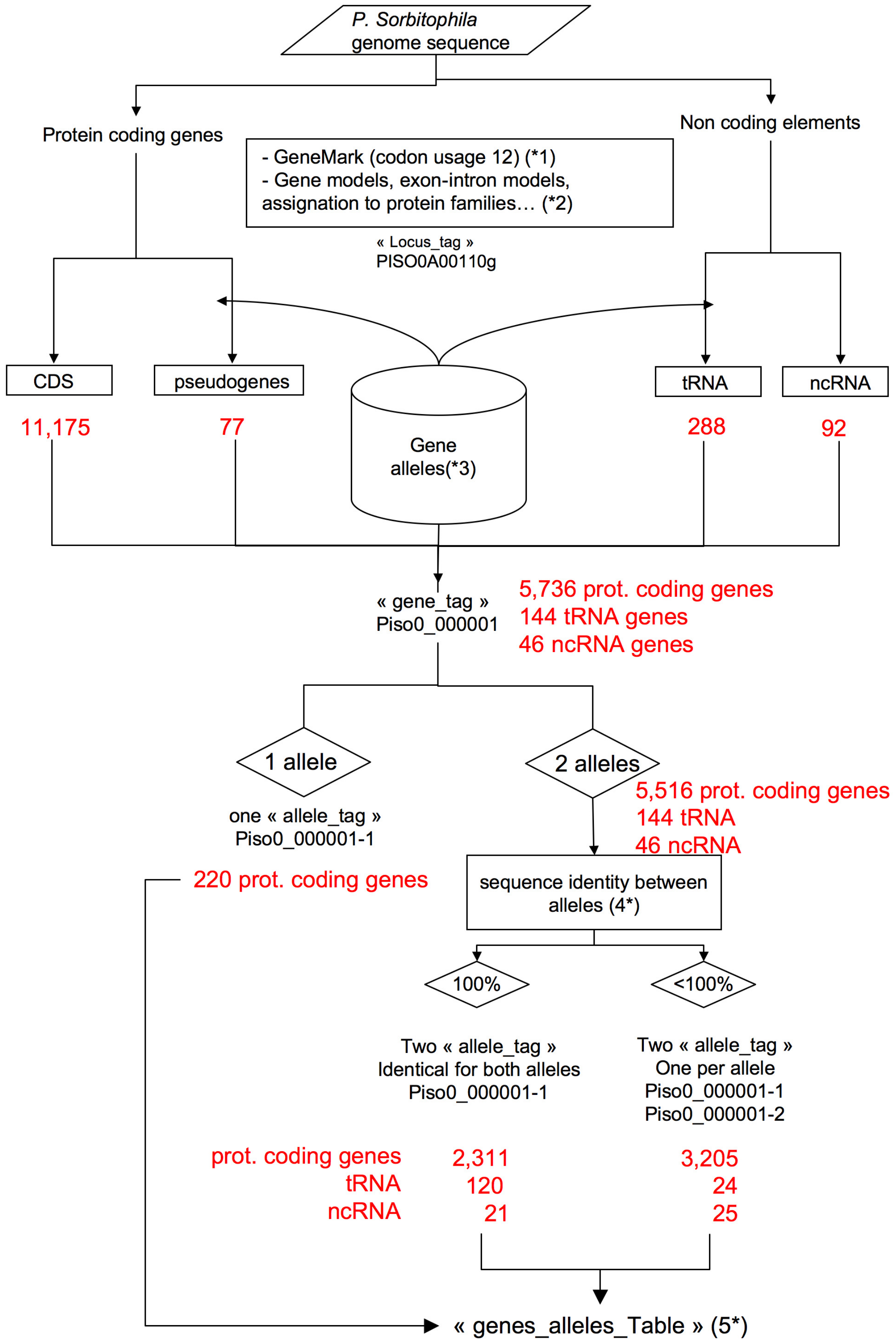
P. Sorbitophila
genome sequence
Protein coding genes
Non coding elements
- GeneMark (codon usage 12) (*1)
- Gene models, exon-intron models, assignation to protein families… (*2)
« Locus_tag »
PISO0A00110g
CDS
pseudogenes
Gene alleles(*3)
tRNA
ncRNA
11,175
77
288
92
« gene_tag »
Piso0_000001
5,736 prot. coding genes
144 tRNA genes
46 ncRNA genes
1 allele
2 alleles
one « allele_tag »
Piso0_000001-1
5,516 prot. coding genes
144 tRNA
46 ncRNA
220 prot. coding genes
sequence identity between alleles (4*)
100%
<100%
Two « allele_tag »
Identical for both alleles
Piso0_000001-1
Two « allele_tag »
One per allele
Piso0_000001-1
Piso0_000001-2
prot. coding genes
tRNA
ncRNA
2,311
120
21
3,205
24
25
« genes_alleles_Table » (5*)

Figure S6 Flowchart for the prediction and the annotation of each chromosomal feature in *P. sorbitophila* genome.
*1 Alternative Yeast Nuclear Code (http://www.ncbi.nlm.nih.gov/Taxonomy/Utils/wprintgc.cgi,transl_table=12)
*2 Gene detection and annotation according to the previously developed method (Souciet *et al*., 2009). Each feature is characterized by a unique "locus_tag" representing its chromosomal position, an example of "locus_tag" is given.
3 For protein-coding genes, tRNA and ncRNA, annotations in (2) were homogenized regarding to the gene synteny between chromosomes forming a pair. Each region of synteny loss was manually checked to assess the presence/absence of the considered gene/pseudogene. For each pair of chromosomes, we considered two syntenic genes/pseudogenes that share the same annotation as two variants of the same gene. They have the same "gene_tag" but can differed for the "allele_tag" (4*)
4* The two variants of a gene were compared at the nucleotide level along the whole length, using Needle from EMBOSS package (Rice *et al*., 2000). Same "alleles_tag" were attributed only in case of 100% identity.
*5 Relation between loci, gene and alleles are available in the "genes _alleles_table" at http://www.genolevures.org/

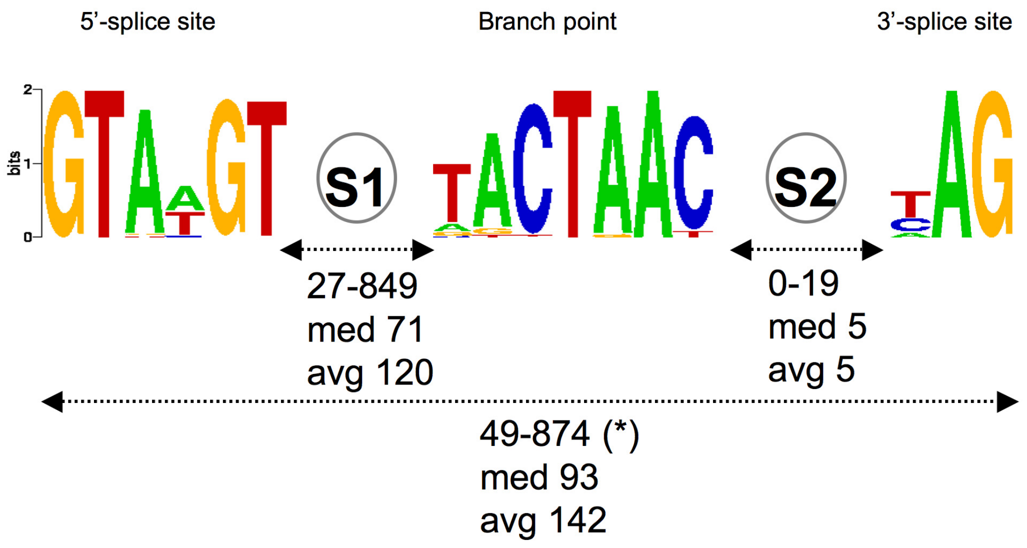

Figure S7 Splicing pattern determined for spliceosomal introns detected in *P. sorbitophila* genome. Minimal and maximal distances observed between exons (*), between the 5'-splice site and branch point (S1) and between the branch point and 3'-splice site (S2) are indicated, as well as the median (med) and the average (avg) values.

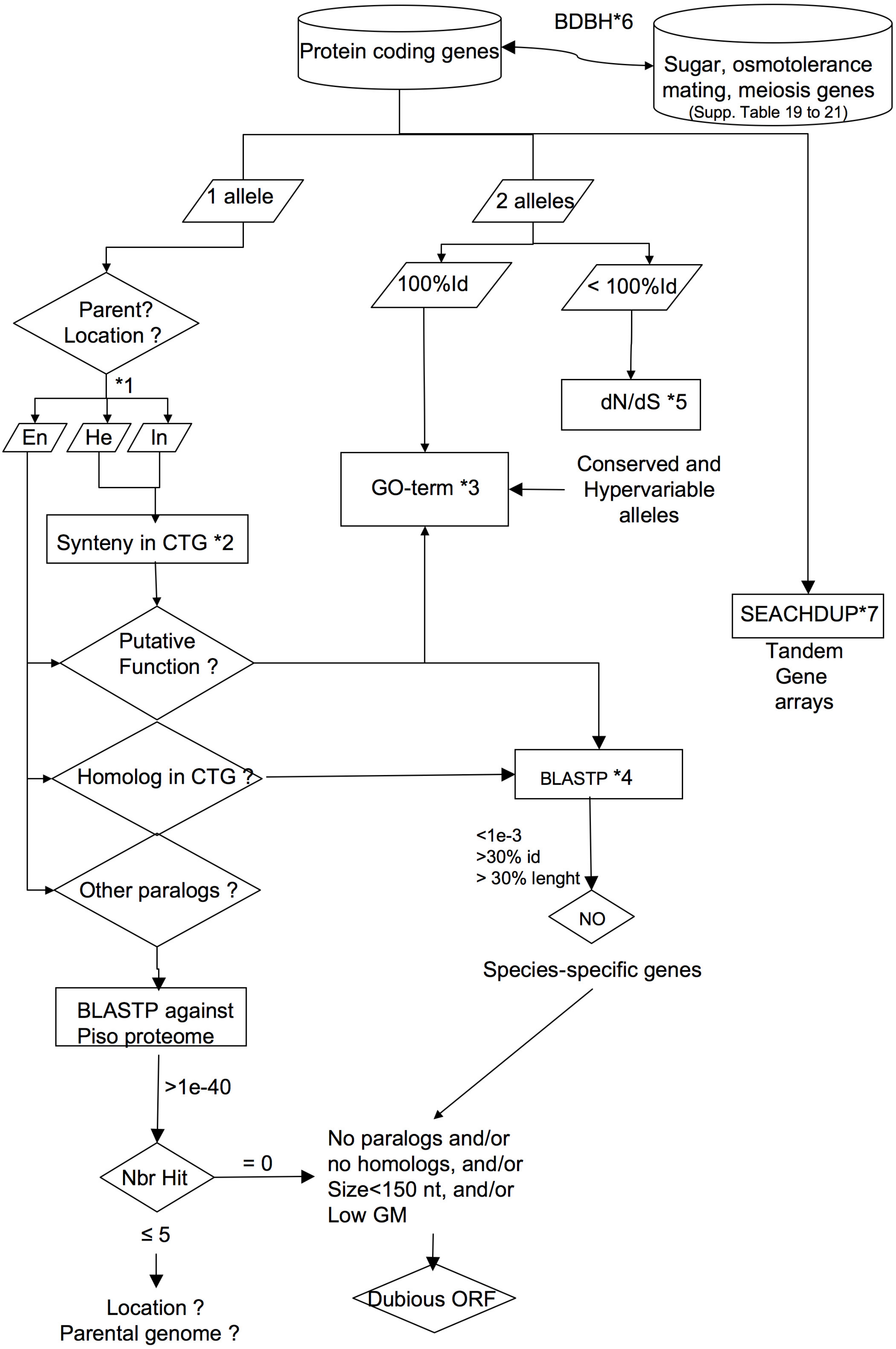
Protein coding genes
BDBH*6
Sugar, osmotolerance
mating, meiosis genes
(Supp. Table 19 to 21)
1 allele
2 alleles
Parent?
Location ?
*1
En
He
In
100%Id
< 100%Id
dN/dS *5
GO-term *3
Conserved and
Hypervariable
alleles
Synteny in CTG *2
SEACHDUP*7
Tandem
Gene
arrays
Putative
Function ?
Homolog in CTG ?
BLASTP *4
<1e-3
>30% id
> 30% lenght
NO
Other paralogs ?
Species-specific genes
BLASTP against
Piso proteome
>1e-40
Nbr Hit
= 0
No paralogs and/or
no homologs, and/or
Size<150 nt, and/or
Low GM
≤ 5
Location ?
Parental genome ?
Dubious ORF

Figure S8 Flowchart for gene analyses and comparisons.

*1 « he », « en » and « in » refer to **he**terozygous regions, **en**d of contig and E/F/I/J translocation breakpo**in**t, respectively. Location and parental subgenome attribution were determined using the table of genes and alleles.

*2 Synteny was analysed first by searching orthologs of neighbor genes in *D. hansenii* using protein families (Sherman *et al*., 2009) and the genome browser available for this species at http://www.genolevures.org/ (Souciet *et al*., 2009). Gene orders in *C. guilliermondii, P. stipitis* and *C. albicans* were obtained from the "Yeast Gene Order Browser" web site http://wolfe.gen.tcd.ie/ygob (Byrne and Wolfe, 2006) using *D. hansenii* genes as queries.

*3 GO-Slim terms were associated to *P. sorbitophila* genes on the basis of orthology with *S. cerevisiae* gene products, considering that all GO-Slim terms of a *S. cerevisiae* gene are transferable to its ortholog. In order to define the functions over or underrepresented in a given group of genes, GO-Slim frequencies for them and for all other genes were calculated and statistically compared.

*4 Species-specific genes were extracted from the whole set of *P. sorbitophila* genes using BLASTP (Altschul *et al*., 1990) against *P. stipitis, C. guilliermondii, C. lusitaniae, C. albicans*, *C. dubliniensis*, *C. tropicalis*, *C. parapsilosis*, *L. elongisporus* and *D. hansenii* proteomes with 1.e-3 as threshold for the expect value and 30% of identity for at least 30% of the query sequence as the threshold for the length of the alignment.

*5 The ratio of non-synonymous substitutions (dN) per synonymous substitutions (dS) was calculated for each gene located in heterozygous regions and having two coding alleles by the following procedure**:** protein sequences for each allelic pair were aligned using *ClustalW2* (Thompson *et al*., 2002). The nucleotide sequences were aligned with *tranalign* from the *EMBOSS* package (Rice *et al*., 2000) using the corresponding set of aligned protein sequences for each allele pair to obtain the nucleic acid sequence translation of the protein alignment, and using the alternative yeast nuclear genetic code (transl_table=12). The dN/dS ratio for the obtained alignment was performed using the *yn00* program from the *PAML package* (Yang, 2007) and calculated by the method of Yang and Nielsen (2000)

*6 Homologs in *P. sorbitophila* were identified by reciprocal blast searches according to the method described in Mushegian and Koonin (1996). Alignments for genes involved in sugar degradation and osmotolerance were manually curated.

*7 Tandem gene arrays were detected by analysing similarities between neighbor genes using the previously developed method described in Despons *et al*. (2010).

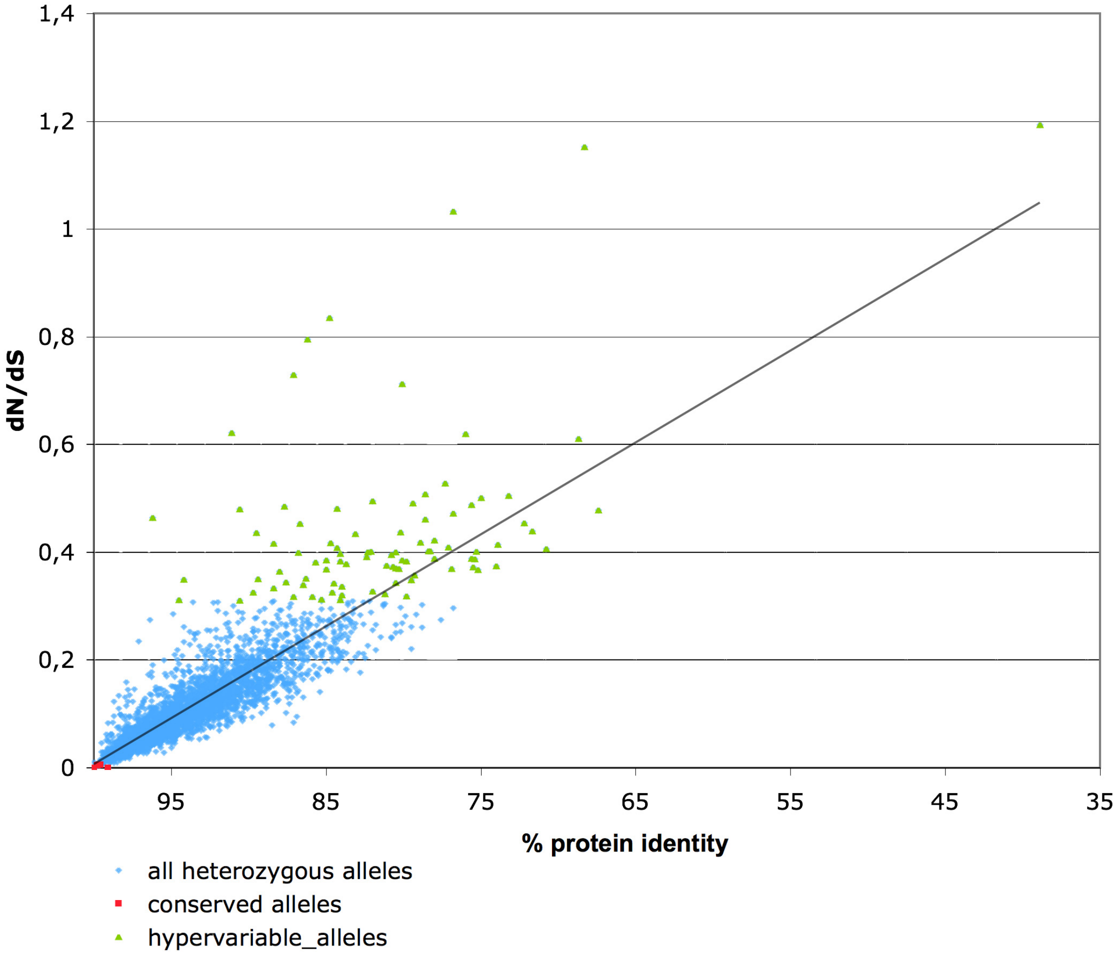

Figure S9 Distribution of dN/dS values according to the protein sequence identity. The dN/dS ration (Y-axe) was calculated for 3,111 allele pairs aligned without conflicts (see Supp Fig. 8 for method). Protein identity (X-axe) was calculated for the aligned part of the sequences. Best fit line is also indicated (R^2= 0.7311). dN/dS mean value = 0.121, median= 0.102 [0.063-0.156]. Three genes show a dN/dS value > to 1 but present also CDS size of 225 to 339 nt and have no other homologs. They likely correspond to dubious ORFs.

3. Ribosomal DNA

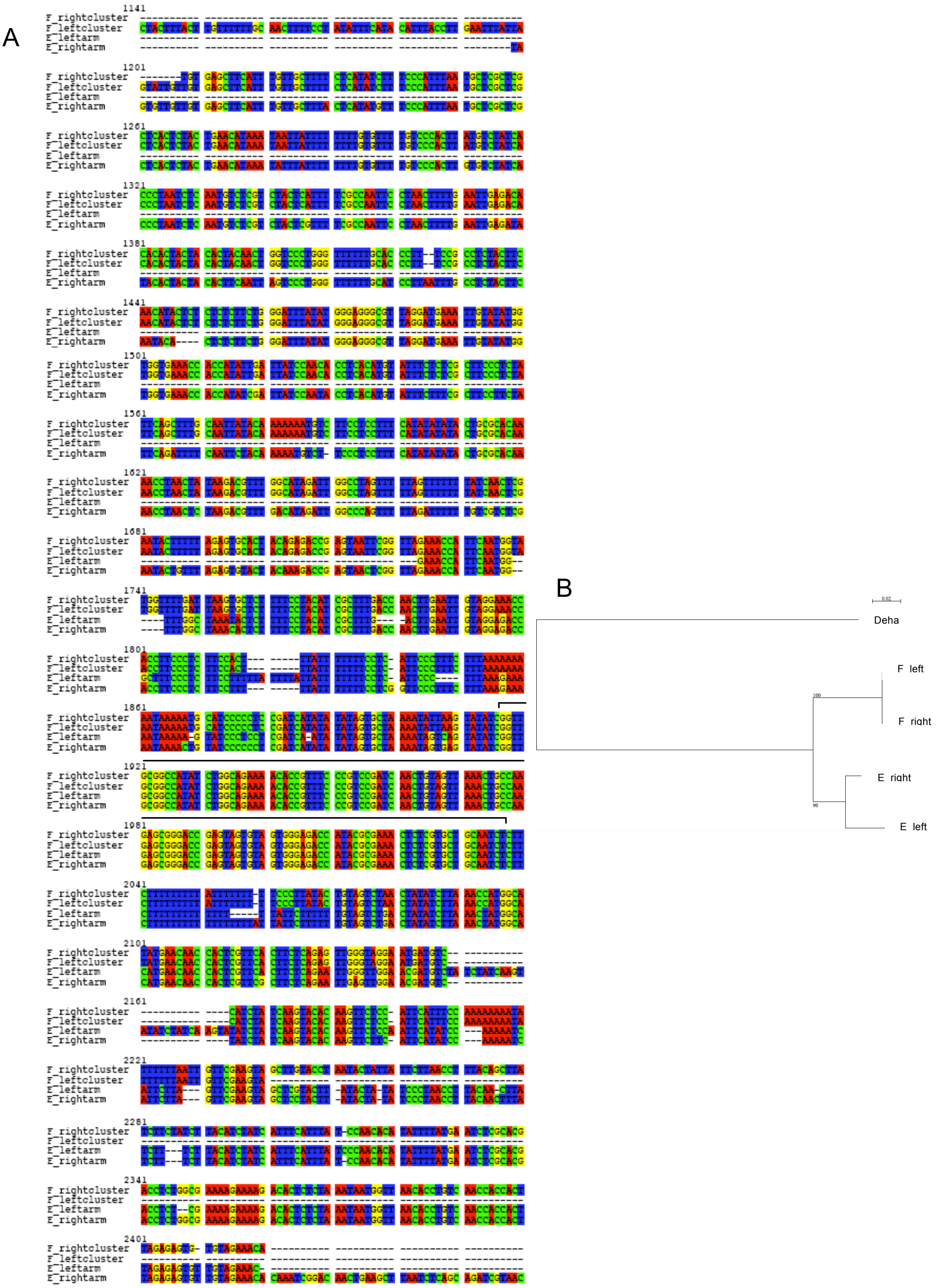

Figure S10 Comparison of 5S rDNA sequences. (A) Multiple alignment of the rDNA loci performed with Muscle software (Edgard, 2004) and visualized with Jalview (Waterhouse *et al*., 2009). E_leftarm and E_rightarm correspond to both rDNA loci on chr. E, F_leftcluster and F_rightcluster correspond to the 5S sequences flanking the 73 tandem repeats (Fig. 4). (B)

Phylogenetic tree calculated using the Bio-NJ distance method in Seaview (Gouy *et al*., 2010) for 100 replicata and D. hansenii as outgroup.

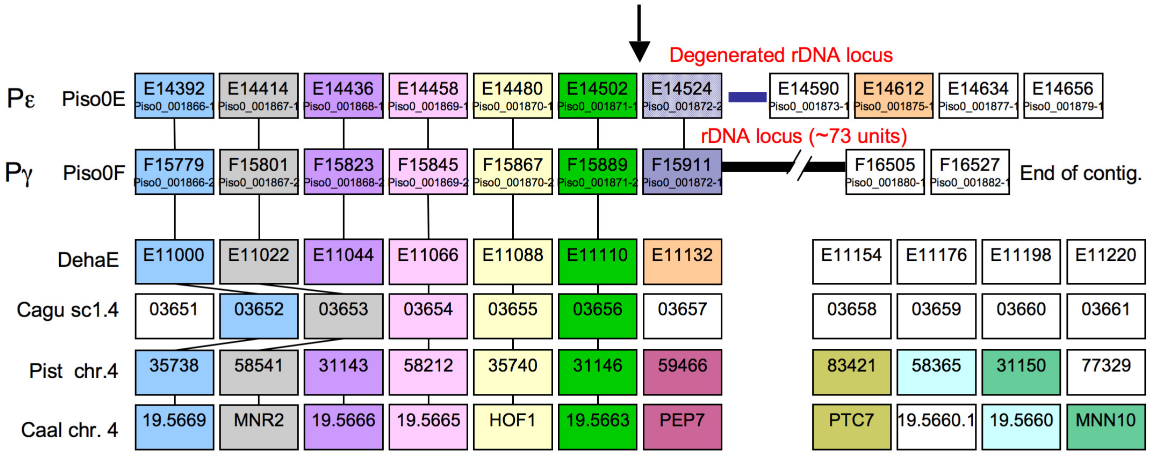

Figure S11 Synteny conservation around the rDNA clusters located on the left arm of *P. sorbitophila* chr E and F. Within the five CTG species, orthologous genes (or alleles for *P. sorbitophila*) are represented by same colors and line connected. The locus name is indicated for each species according to the published nomenclature. For *P. sorbitophila*, both loci and alleles are indicated (see also Figure S8). Hatched box corresponds to pseudogene. Synteny breakpoint is represented by an arrow.

4. Mitochondrial genome

A recent phylogenetic analysis of the CTG yeast mitochondrial genomes (mt) places the *P. sorbitophila* mt genome at the base of this group: *P. farinosa* (CBS 185), *P. stipitis*, *D. hansenii* and *P. guillermondii* mt genomes are more closely related to each other than to *P. sorbitophila* (Jung *et al*., 2010). Two facts suggest that the *P. sorbitophila* mt genome is inherited from the Pε parent: i) its mt genome is very distant from others *Pichia* species, and ii) *P. sorbitophila* mt *COX2* gene sequence diverges from the one sequenced in *P. farinosa* CBS 2001 (Mallet *et al*., in preparation), a strain very closely related to Pγ (Table S4).

5. tRNA and co-transcribed tRNA, tRNA gene usage

The *P. sorbitophila* genome contains a total of 144 tRNA genes (Table S14), all of them being represented by two alleles, 88 in heterozygous and 56 in homozygous regions. This value is significantly lower than for *D. hansenii* with 200 tRNA genes (Table S14). The 88 tRNA genes located in heterozygous regions show a total of 18 SNP between alleles (outside intronic and extra arm sequences), corresponding to less than 0.3% of sequence divergence. Groups of two neighbouring and co-oriented tRNA genes are present in one or four copies in the genome (Table S15). The neighbouring tRNAs genes are separated by only eight to 13 nucleotides suggesting that they are co-transcribed (Acker *et al*., 2008).

The codon and tRNA gene usages were also compared with *D. hansenii* (Table S16). In *P. sorbitophila*, as in the other yeasts of the CTG group (Santos *et al*., 1996, Perreau *et al*., 1999, Marck *et al*., 2006), the decoding properties of both tRNA-Leu (AAG, reading CTT, CTC and CTA codons) and tRNA-Ser (CAG, reading the usual CTG codon as Ser) are finely tuned by two unusual G nucleotides located 5' of the anticodon (G32 and G33, respectively, instead of the predominant C32 and U33).

6. Other noncoding RNA genes

We were able to identify 46 ncRNA genes (Supp. Table 17): the small nuclear RNAs (U1, U2, U4, U5 and U6); the RNA components of the RNase P; the signal recognition particle; 17 H/ACA and 35 C/D snoRNAs. All these ncRNA are evenly distributed over the 7 pairs of chromosomes including 17 in the homozygous chromosomal regions (essentially snoRNAs). The remaining 29 ncRNAs (found in heterozygous regions) are all represented by two highly similar alleles sharing on average 96.0 % of nucleotide identity. Compared to the average protein-coding genes identity (90.9 % in the coding sequence), ncRNA genes are therefore highly conserved.

7. Synteny breaks between Pγ and Pε subgenomes

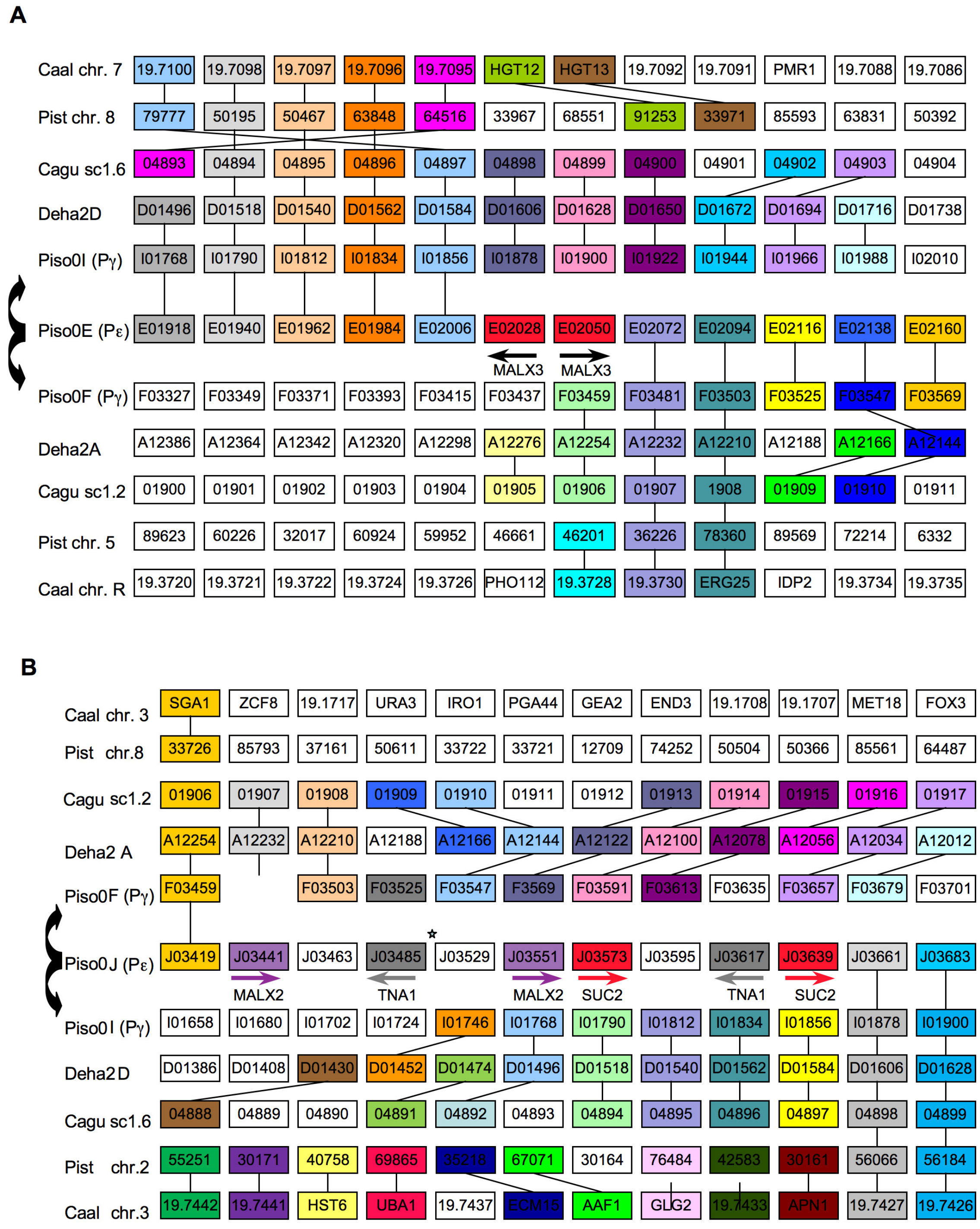

Figure S12 Comparison of synteny maps at the E/F/I/J reciprocal translocation between CTG yeasts. (A) Gene order on chr. E showing a synteny shift from chr. I to chr. F. (B) Gene order on chr. J showing a synteny shift from chr. F to chr. I. Tandemly duplicated genes and pseudogenes are indicated by arrows and star, respectively. Orthologs are represented by same color and line-connected.

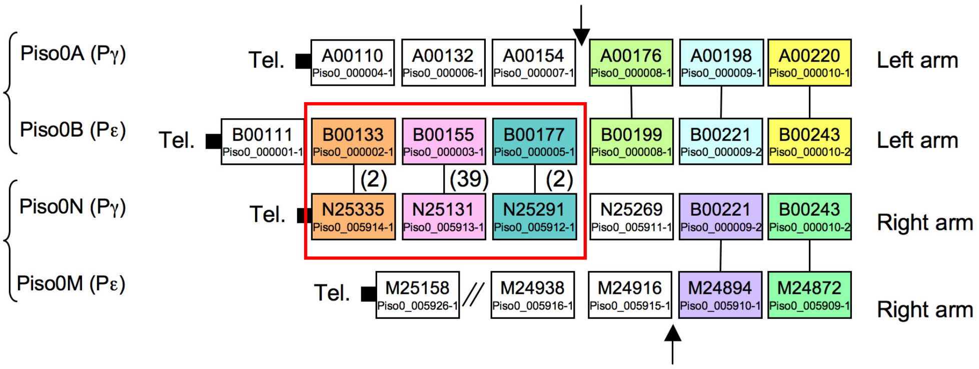

Figure S13 Gene location movement between two subtelomeric regions. Alleles are represented by connected boxes of same color. The comparison between subtelomeric regions of A/B and M/N chromosomal pairs shows breaks of synteny (arrows). The last three genes (toward the telomere) on chr. N (red box) have no equivalent allelic position on chr M. The second copy of these genes is found in the subtelomeric region of chr B, in same orientation. The number of paralogs identified for each gene is indicated in brackets. Among the three genes, two are represented by these only two copies in the genome of *P. sorbitophila*.

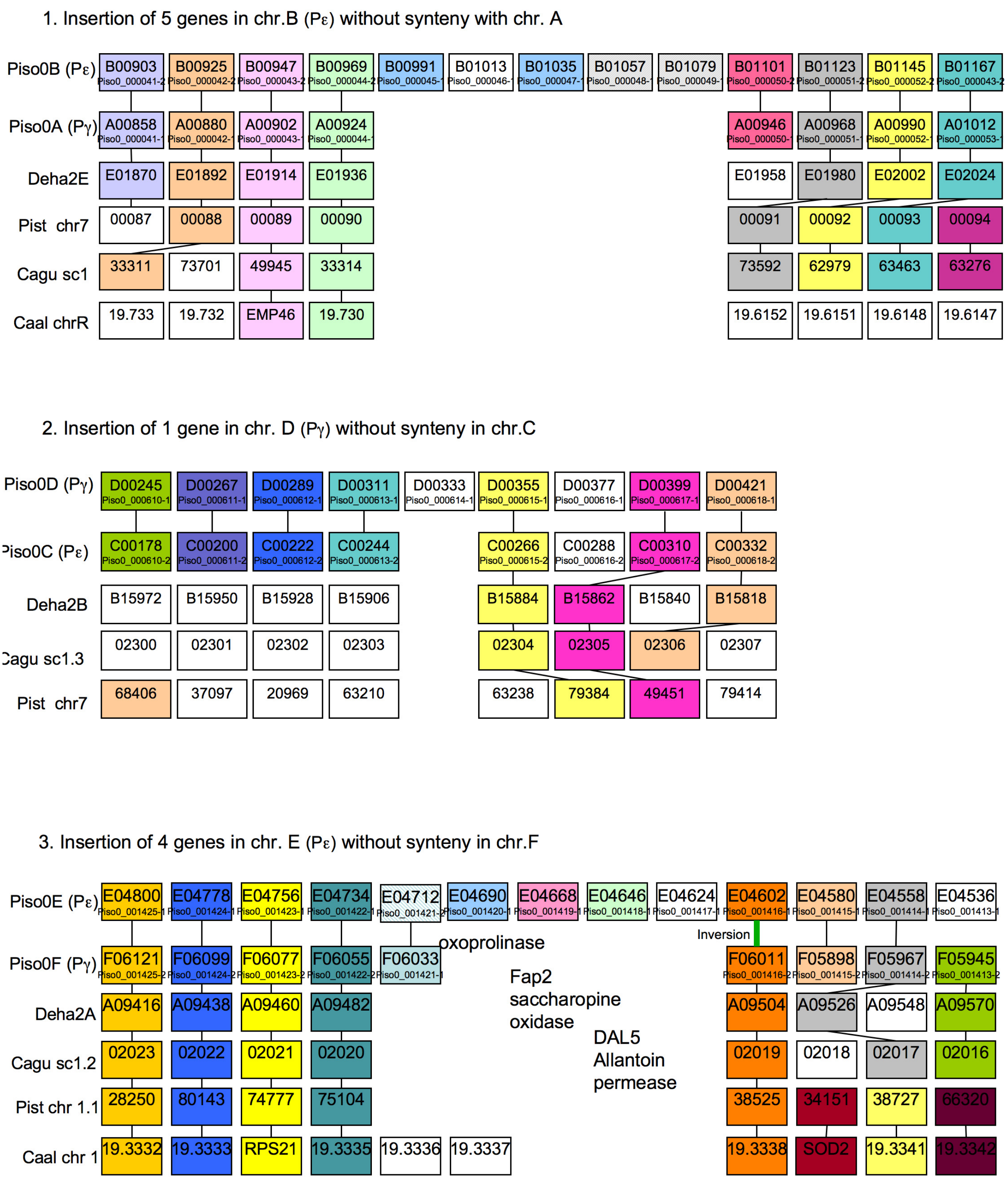

Figure S14 Comparison of synteny maps at single allele gene positions between CTG yeasts (seven studied cases). Alleles of genes (*P. sorbitophila*) and orthologs are represented by same color and line-connected. Hatched box corresponds to a pseudogene. One case of inversion is indicated by a green connector between alleles.

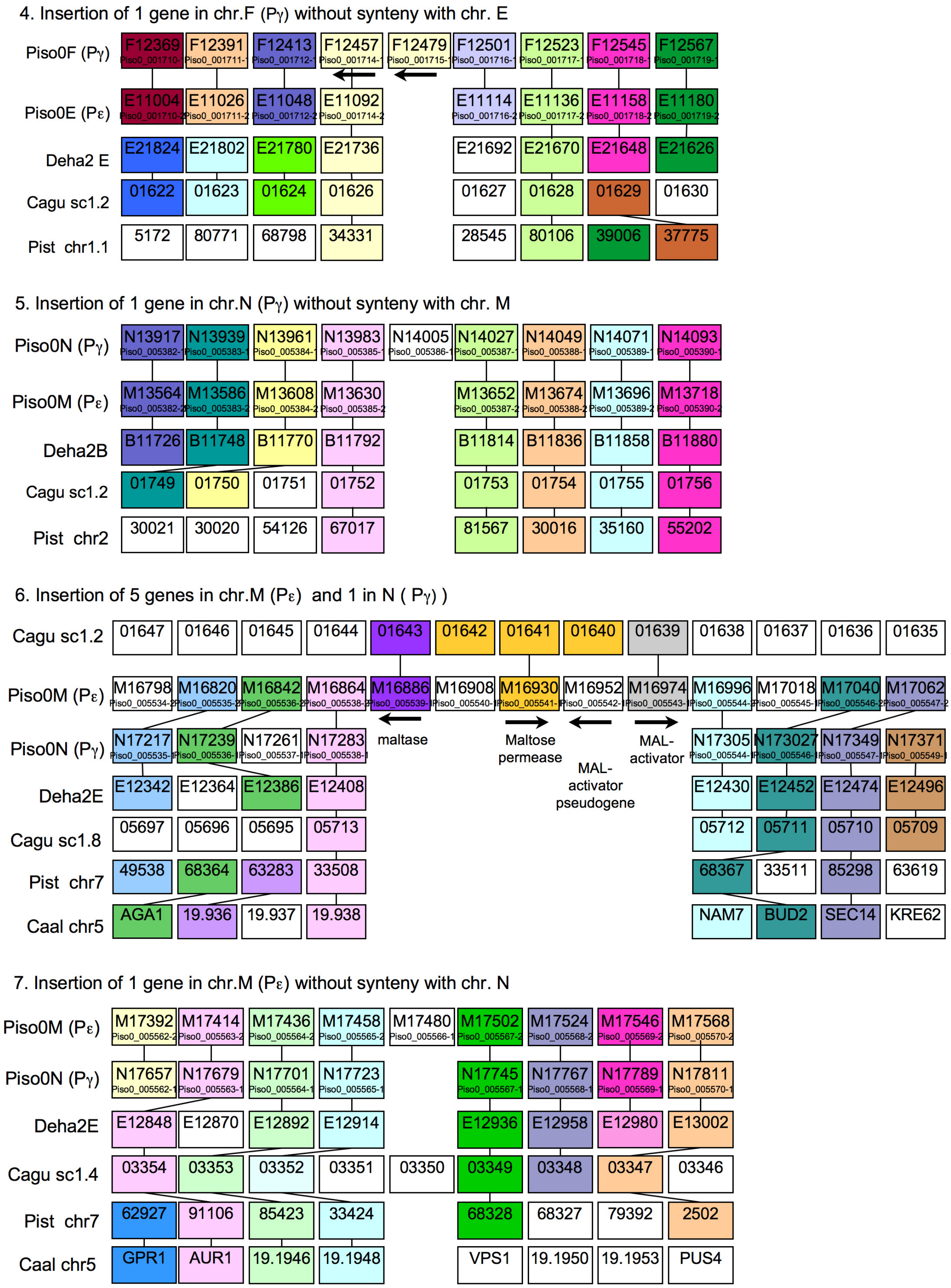

Figure S14 Comparison of synteny maps at single allele gene positions between CTG yeasts (seven studied cases), cases four to seven.

8. Mating type

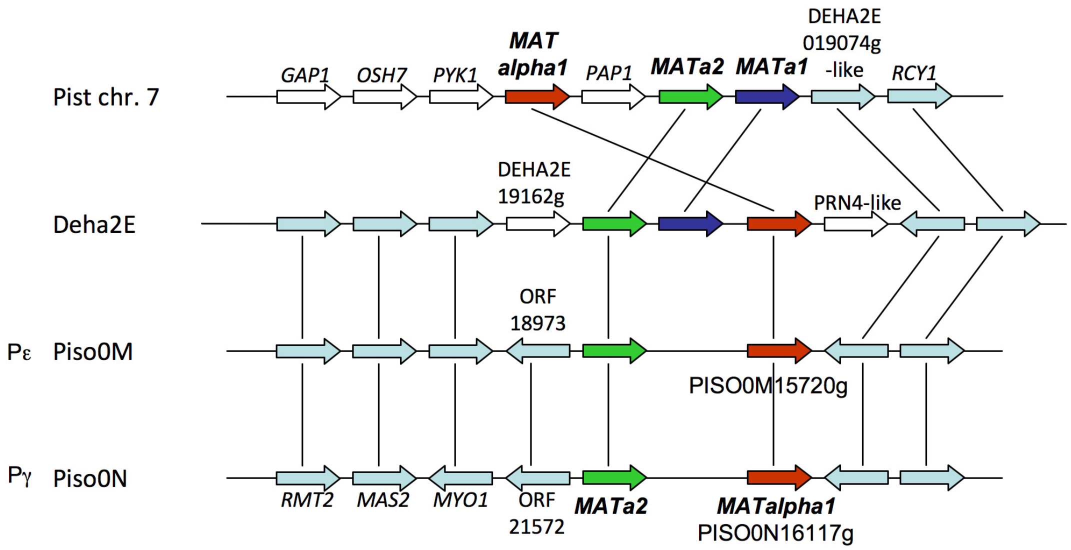

Figure S15 Comparative organization of the mating type locus (MTL) in *P. sorbitophila*, *D. hansenii* and *P. stipitis*. Conserved MTL genes are coloured: alpha idiomorph, in red; a1, in blue; a2, in green. Orthologous genes line connected. In *P. stipitis, MATa1* is annotated as *YOX2* and *MATalpha1* is not annotated.

References

- Acker, J. l. *et al*., 2008 Dicistronic tRNA-5S rRNA genes in *Yarrowia lipolytica*: an alternative TFIIIA-independent way for expression of 5S rRNA genes. *Nucleic Acids Res.* **36**: 5832-5844.
- Altschul, S. F., Gish, W., Miller, W., Myers, E. W. & Lipman, D. J., 1990 Basic local alignment search tool. *J Mol. Biol.* **215**: 403-410.
- Bon, E. *et al*., 2003 Molecular evolution of eukaryotic genomes: hemiascomycetous yeast spliceosomal introns. *Nucleic Acids Res.* **31**: 1121-1135.
- Brudno, M. *et al*., 2003 LAGAN and Multi-LAGAN: efficient tools for large-scale multiple alignment of genomic DNA. *Genome Res.* **13**: 721-731.
- Butler, G. *et al*., 2009 Evolution of pathogenicity and sexual reproduction in eight *Candida* genomes. Nature **459**: 657-62.
- Byrne, K. P. & Wolfe, K. H., 2006 Visualizing syntenic relationships among the hemiascomycetes with the Yeast Gene Order Browser. *Nucleic Acids Res.* **34**: D452-455.
- Crick, F. H., 1966 Codon anticodon pairing: the wobble hypothesis. *J. Mol. Biol.* **19**: 548-555.
- Despons, L. *et al*., 2010 Genome-wide computational prediction of tandem gene arrays: application in yeasts. *BMC Genomics* **11**: 56
- Dujon, B. *et al*., 2004 Genome evolution in yeasts. Nature **430**: 35-44.
- Edgar, R. C., 2004 MUSCLE: multiple sequence alignment with high accuracy and high throughput. *Nucleic Acids Res.* **32**: 1792-1797.
- Gouy, M., Guindon, S. p. & Gascuel, O., 2010 SeaView version 4: A multiplatform graphical user interface for sequence alignment and phylogenetic tree building. *Mol Biol Evol* **27**: 221-224.
- Griffiths-Jones, S., 2005 Annotating non-coding RNAs with Rfam. *Curr Protoc Bioinformatics.* Chapter 12, Unit 12.5.
- Jackson, A. P. *et al*., 2009 Comparative genomics of the fungal pathogens *Candida dubliniensis* and *Candida albicans*. *Genome Res.* **19**: 2231-2244.
- Jeffries, T. W. *et al*., 2007 Genome sequence of the lignocellulose-bioconverting and xylose-fermenting yeast *Pichia stipitis*. *Nat. Biotechnol.* **25**: 319-326.
- Jones, T. *et al*., 2004 The diploid genome sequence of *Candida albicans*. *Proc Nat Acad Sci U S A.* **101**: 7329-7334.
- Jung, P. P. *et al*., 2010 Complete mitochondrial genome sequence of the yeast *Pichia farinosa* and comparative analysis of closely related species. *Curr. Genet.* **56**: 507-515.
- Lopez, P. J. & Séraphin, B., 1999 Genomic-scale quantitative analysis of yeast pre-mRNA splicing: implications for splice-site recognition. *RNA.* **5**: 1135-1137.
- Lowe, T. M. & Eddy, S. R., 1997 tRNAscan-SE: a program for improved detection of transfer RNA genes in genomic sequence. *Nucleic Acids Res.* **25**: 955-964.
- Marck, C. & Grosjean, H., 2002 tRNomics: analysis of tRNA genes from 50 genomes of Eukarya, Archaea, and Bacteria reveals anticodon-sparing strategies and domain-specific features. *RNA.* **8**: 1189-1232.
- Marck, C. *et al*., 2006 The RNA polymerase III-dependent family of genes in hemiascomycetes: comparative RNomics, decoding strategies, transcription and evolutionary implications. *Nucleic Acids Res.* **34**: 1816-1835.
- Mattanovich, D. *et al*., 2009 Open access to sequence: browsing the *Pichia pastoris* genome. *Microb Cell Fact.* **8**: 53.
- Mushegian, A. R. & Koonin, E. V., 1996 A minimal gene set for cellular life derived by comparison of complete bacterial genomes. *Proc Nat Acad Sci U S A*. **93**: 10268-10273.
- Nawrocki, E. P., Kolbe, D. L. & Eddy, S. R., 2009 Infernal 1.0: inference of RNA alignments. *Bioinformatics*. **25**: 1335-1337.
- Ning, Z., Cox, A. J. & Mullikin, J. C., 2001 SSAHA: a fast search method for large DNA databases. *Genome Res.* **11**: 1725-1729.
- Perreau,V.M., *et al*., 1999 The *Candida albicans* CUG-decoding ser-tRNA has an atypical anticodon stem–loop structure. *J. Mol. Biol.*, **293** :1039–1053
- Rice, P., Longden, I. & Bleasby, A., 2000 EMBOSS: the European Molecular Biology Open Software Suite. *Trends Genet.* **16**: 276-277.
- Rolland, T. & Dujon, B., 2011 Yeasty clocks: dating genomic changes in yeasts. *C. R. Biologies*. in press.
- Sacerdot, C. *et al*., 2008 Promiscuous DNA in the nuclear genomes of hemiascomycetous yeasts. *FEMS Yeast Res.* **8**: 846-857.
- Santos,M. A., Perreau,V. M. & Tuite, M. F., 1996 Transfer RNA structural change is a key element in the reassignment of the CUG codon in *Candida albicans*. *EMBO J*. **15** : 5060–5068.
- Sherman, D. J. *et al*., 2009 Génolevures: protein families and synteny among complete hemiascomycetous yeast proteomes and genomes. *Nucleic Acids Res.* **37**: D550-554.
- Skrzypek, M. S. *et al*., 2010 New tools at the Candida Genome Database: biochemical pathways and full-text literature search. *Nucleic Acids Res.* **38**: D428-432.
- Souciet, J. L. *et al*., 2009 Comparative genomics of protoploid *Saccharomycetaceae*. *Genome Res*. **19**: 1696.
- Suzuki, C., Yoshida, N., Okano, E., Kawasumi, T. & Kashiwagi, Y., 2003 Cloning and chromosomal mapping of *URA3* genes of *Pichia farinosa* and *P. sorbitophila* encoding orotidine-5'-phosphate decarboxylase. *Yeast*. **20**: 905-912.
- Thompson, J. D., Gibson, T. J. & Higgins, D. G., 2002 Multiple sequence alignment using ClustalW and ClustalX. *Curr Protoc Bioinformatics*. Chapter 2, Unit 2.3.
- Vezinhet, F., Blondin, B. & Hallet, J.-N., 1990 Chromosomal DNA patterns and mitochondrial DNA polymorphism as tools for identification of enological strains of *Saccharomyces cerevisiae*. *Appl Microbiol Biotechnol.* **32:** 568-571.

- Waterhouse, A. M., Procter, J. B., Martin, D. M. A., Clamp, M. l. & Barton, G. J., 2009 Jalview Version 2--a multiple sequence alignment editor and analysis workbench. *Bioinformatics*. **25**: 1189-1191.
- Yang, Z. PAML 4: phylogenetic analysis by maximum likelihood. *Mol Biol Evol.* **24**, 1586-1591 (2007).
- Yang, Z. & Nielsen, R. Estimating synonymous and nonsynonymous substitution rates under realistic evolutionary models. *Mol Biol Evol.* **17**, 32-43 (2000).

Table S1 Heterozygous and homozygous parts of *P. sorbitophila* genome

chr. Pair*	chr.	Chr. Size (nt)	Size of homozygous region (nt)	Size of heterozygous region** (nt)	Homozygous Part (%)	Heterozygous Part †(%)	Size of hetero. reg. w/o ins/del√ (nt)	% seq. identity in hetero. regions†	% seq. identity in hetero. regions w/o ins/del¥	Telomeric repeats &
A/B	Chr. A	1,055,225	498,502	556,723	47.2	52.8	551,536	87.45	89.74	L and R
	Chr. B	1,063,002	498,502	564,500	46.9	53.1	550,398			R
C/D	Chr. C	1,095,816	467,624	628,192	42.7	57.3	622,613	87.18	88.75	R
	Chr. D	1,102,865	467,624	635,241	42.4	57.6	622,743			R
E/I	Chr. E	1,307,235	0	178,135	0	100	149,929	70.61	87.10	-
	Chr. I	1,666,063	0	162,406	0	100	151,514			-
E/F	Chr. E	1,307,235	0	1,129,100	0	100	1,068,026	85.07	89.47	L and R
	Chr. F	1,407,903	0	1,119,295	0	100	1,071,047			L
F/J	Chr. F	1,407,903	0	288,608	0	100	284,415	88.33	89.31	-
	Chr. J	1,803,284	0	285,346	0	100	283,178			-
I/J	Chr. I	1,666,063	0	1,503,657	0	100	1,202,358	88.63	89.14	R
	Chr. J	1,803,284	0	1,517,939	0	100	1,196,297			R
G/H	Chr. G	1,423,303	1,423,303	0	100	0	-	-	-	L and R
	Chr. H	1,423,303	1,423,303	0	100	0	-			L and R
K/L	Chr. K	1,933,849	1,933,849	0	100	0	-	-	-	L
	Chr. L	1,933,849	1,933,849	0	100	0	-			L
M/N	Chr. M	2,121,241	0	2,121,241	0	100	2,048,627	85.64	89.10	L and R
	Chr. N	2,122,704	0	2,122,704	0	100	2,049,178			L
**Total genome**		**21,459,642**	**8,646,556**	**12,813,086**	**40.3**	**59.7**	**11,851,959**	**84.70**	**89.16**	-

* according to identity (Figure S3). ** Size of the whole sequence of each heterozygous region, from telomeric repeats until the last SNP identified. † Calculated using the complete heterozygous regions **. √ Size of the heterozygous regions excluding areas of identity drop, i.e telomeric and subtelomeric non aligned sequences and internal insertions/deletions. ¥ percentage of identity calculated on the basis of the global alignment of regions presenting no drop of identity (√). Sequences were aligned using Stretcher from EMBOSS (Rice et al., 2000). The total percentage of identity is the sum of all identity in heterozygous regions divided by the sum of all aligned positions. & Telomeres were detected at the ends of contigs by searching repeats rich in C/A (inward orientation) and in G/T (outward orientation). They were found at both ends for chr. A, E, G, H and M, at the right end for chr. B, C, D, I and J, and at the left end for chr. F, K, L and N. The motif CCCAAACA is repeated without variation.

Table S2 Sequence polymorphism in homozygous regions of the *P. sorbitophila* genome

chr. pair	size_region of homozygosity per chr.	size of covered area in region of homozygosity	% of coverage in region of homozygosity	Nbr. SNP in covered area	Nbre of Indel in covered area	Average SNP/indel density
A/B	498,502	393,074	78.85	22	0	1/35,734
C/D	467,624	411,796	88.06	15	1	1/51,474
G/H	1,423,303	1,421,984	99.90	158	0	1/18,000
K/L	1,933,849	1,933,590	99.98	186	1	1/20,680

The SNP detection procedure is based on the mapping of the trimmed reads on the genome reference sequence with SSAHA2 (Ning *et al*., 2001). The variant positions were filtered according to the identified bases quality (Q>25), the number of reads containing the SNP and the local coverage.

Table S3 NUMTs in the nuclear genome of *Pichia sorbitophila*

Nb	Mitochondrial coordinates	Mitochondrial gene/intergene	orienta-tion	size (nt)	Percentage identity*	Chr.	Chromosomal coordinates	Parental subgenome	NUMT organization	Cluster type	Allele organization
p1	20708-20744	cox1 CDS ai5 ai4	>	37	97	Piso0A	984134-984170	Pe	single		homozygous (with NUMT p5)
p2	38123-38152	Nad2	>	30	96	Piso0A	1035013-1035042	Pe	cluster 1	mosaic	homozygous (with mosaic 2)
p3	19871-19922	cox1 CDS ai5 ai4	>	52	94	Piso0A	1035162-1035213	Pe	cluster 1	mosaic	
p4	19977-20063	cox1 CDS ai5 ai4	>	87	83	Piso0A	1035220-1035306	Pe	cluster 1	mosaic	
p5	20708-20744	cox1 CDS ai5 ai4	>	37	97	Piso0B	991911-991947	Pe	single		homozygous (with NUMT p1)
p6	38123-38152	Nad2	>	30	96	Piso0B	1042790-1042819	Pe	cluster 2	mosaic	homozygous (with mosaic 1)
p7	19871-19922	cox1 CDS ai5 ai4	>	52	94	Piso0B	1042939-1042990	Pe	cluster 2	mosaic	
p8	19977-20063	cox1 CDS ai5 ai4	>	87	83	Piso0B	1042997-1043083	Pe	cluster 2	mosaic	
p9	35906-35958	tRNA-Glu	<	53	100	Piso0E	81462-81514	Pe	single		hemizygous (absent on Piso0I)
p10	27200-27253	Cox1	>	53	90	Piso0E	310115-310167	Pe	cluster 3	mosaic	hemizygous (absent on Piso0F)
p11	33141-33180	Nad1	>	40	90	Piso0E	310167-310206	Pe	cluster 3	mosaic	
p12	36888-36929	Atp6	>	42	95	Piso0E	629315-629356	Pe	single		hemizygous (absent on Piso0F)
p13	14902-15036	Nad5	<	135	92	Piso0E	673200-673334	Pe	single		hemizygous (absent on Piso0F)
p14	21204-21227	cox1 CDS ai5 ai4	<	24	95	Piso0G	481070-481093	Pg	single		homozygous (with NUMT p15)
p15	21204-21227	cox1 CDS ai5 ai4	<	24	95	Piso0H	481070-481093	Pg	single		homozygous(withNUMT p14)
p16	33508-33552	Nad1	<	45	91	Piso0I	9413-9457	Pg	cluster	procession	hemizygous (absent on Piso0E)
p17	33267-33492	Nad1	<	224	86	Piso0I	9448-9671	Pg	cluster	procession	
p18	3957-3993	cob bi4 bi3 bi2	>	37	97	Piso0J	783025-783061	Pe	single		hemizygous (absent on Piso0I)
p19	34992-35101	intergene	>	110	98	Piso0J	1034596-1034705	Pe	single		hemizygous (absent on Piso0I)
p20	37866-37916	Nad2	>	51	90	Piso0J	1330982-1331032	Pe	cluster 4	mosaic	hemizygous (absent on Piso0I)
p21	17989-18033	cox1 CDS ai5 ai4 ai3 ai2	>	45	97	Piso0J	1331053-1331097	Pe	cluster 4	mosaic	
p22	20709-20743	cox1 CDS ai5 ai4	<	35	97	Piso0J	1331102-1331136	Pe	cluster 4	mosaic	
p23	10164-10195	tRNA-Phe	>	32	96	Piso0J	1331137-1331168	Pe	cluster 4	mosaic	
p24	28250-28291	Cox1	<	43	93	Piso0M	158431-158473	Pe	single		hemizygous (absent on Piso0N)

NUMTs (NUclear sequences of MiTochondrial origin) in *P. sorbitophila* genome were identified on the basis of the complete published sequence (39,107 bp) of the mitochondrial genome (Jung *et al*., 2010), using BLASTN searches (Altschul *et al*., 1990) and according to the previously developed method described by Sacerdot *et al (2008).* To test the hypothesis that some NUMTs present in the Pγ subgenome were not identified due to poor sequence identities with the *P. sorbitophila* mitochondrial genome, we re-iterated the procedure by using the mitochondrial genome of *P. farinosa* CBS 185, but without obtaining additional NUMTs insertion loci in Pγ. * percentage of identity between NUMTs and mtDNA

Table S4 Sequence identity between *P. farinosa CBS 2001* and *P. sorbitophila* subgenomes

region	feature	chr_1 *	position_1 $	locus_1	Identity_1 to P. farinosa CBS2001 &	subgenome attribution for locus _1 £	chr_2*	position_2 $	locus_2	Identity_2 to P. farinosa CBS2001 &	subgenome attribution for locus _2 £	accession number
heterozygous	gene	B	176663..177097	PISO0B02069g	93	Pε	A	168748..169182	PISO0A01892g	100	Pγ	FN994818
homozygous	gene	B	796873..797371	PISO0B09835g	94	Pε	A	789096..789594	PISO0A09768g	94	Pε	FN994814
homozygous	NUMT p1	B	992082..991685	PISO0B12453g-PISO0B12475g	73 (no NUMT)	Pε	A	983908..984305	PISO0A12386g-PISO0A12408g	73 (no NUMT)	Pε	JQ267632
heterozygous	gene	C	151119..151546	PISO0C01806g	92	Pε	D	158180..158610	PISO0D01895g	100	Pγ	FN994825
heterozygous	gene : Pγ-Pε exchange	C	383527..384113	PISO0C04512g	98-99	Pγ	D	391163..391749	PISO0D04623g	99-97	Pγ	JQ267644
homozygous	gene	C	868158..868568	PISO0C10474g	100	Pγ	D	875207..875617	PISO0D10541g	100	Pγ	FN994821
heterozygous	gene	E	99549..100005	PISO0E01214g	92	Pε	I	84142..84602	PISO0I01042g	100	Pγ	FN994836
heterozygous	gene	E	254393..254869	PISO0E02930g	93	Pε	F	361850..362326	PISO0F04339g	99	Pγ	FN994833
heterozygous	gene	E	1185601..1186066	PISO0E13534g	95	Pε	F	1289721..1290186	PISO0F14921g	100	Pγ	FN994829
heterozygous	gene	J	208409..208838	PISO0J02473g	93	Pε	F	211321..211750	PISO0F02513g	100	Pγ	FN994840
heterozygous	last gene in F/J syneny	J	283861..284508	PISO0J03419g	90	Pε	F	287111..287770	PISO0F03459g	100	Pγ	JQ267642
heterozygous	end of F/J syneny	J	285133..285342	PISO0J03419g	85	Pε	F	288395..288881	PISO0F03459g-PISO0F03481g	100	Pγ	JQ267639
heterozygous	begining of F/E synteny	E	181320..181550	PISO0E02050g-PISO0E02072g	91	Pε					Pγ	
heterozygous	last gene in I/E syneny	E	177149..177618	PISO0E02006g	91	Pε	I	161728..162198	PISO0I01856g	100	Pγ	JQ267640
heterozygous	end of I/E syneny	E	177685..177840	PISO0E02006g-PISO0E02028g	93	Pε	I	162265..162748	PISO0I01856g-PISO0I01878g	99	Pγ	JQ267643
heterozygous	begining of I/J synteny	J	305395..305708	PISO0J03661g	94	Pε					Pγ	
heterozygous	gene	J	516929..517387	PISO0J06081g	91	Pε	I	375063..375521	PISO0I04276g	100	Pγ	FN994853
heterozygous	gene	J	1521249..11521709	PISO0J18269g	93	Pε	I	1384553..1385013	PISO0I16508g	100	Pγ	FN994849
homozygous	gene	G	172080..172490	PISO0G02294g	100	Pγ	H	172080..172490	PISO0H02295g	100	Pγ	FN994846
homozygous	gene	G	301055..301682	PISO0G03856g	100	Pγ	H	301055..301682	PISO0H03857g	100	Pγ	JQ267635

homozygous	gene	G	301813..302440	PISO0G03856g	100	Pγ	H	301813..302440	PISO0H03857g	100	Pγ	JQ267641
homozygous	NUMTs p14-15	G	480960..481261	PISO0G06122g-PISO0G06144g	100	Pγ	H	480960..481261	PISO0H06123g-PISO0H06145g	100	Pγ	JQ267633
homozygous	Intergene	G	1083262..1084635	PISO0G13734g-PISO0G13712g	99	Pγ	H	1083262..1084635	PISO0H13735g-PISO0H13713g	99	Pγ	JQ267636
homozygous	gene	G	1236388..1236848	PISO0G15538g	100	Pγ	H	1236388..1236848	PISO0H15539g	100	Pγ	FN994843
homozygous	intergene	K	100922..101449	PISO0K01286g-PISO0K01264g	99	Pγ	L	100922..101449	PISO0L01287g-PISO0L01265g	99	Pγ	JQ267637
homozygous	gene	K	234563..234998	PISO0K02738g	100	Pγ	L	234563..234998	PISO0L02739g	100	Pγ	FN994860
homozygous	intergene	K	1063363..1063459	PISO0K12528g-PISO0K12506g	100	Pγ	L	1063363..1063459	PISO0L12529g-PISO0L12507g	100	Pγ	**
homozygous	intergene	K	1063482..1063698	PISO0K12528g-PISO0K12550g	100	Pγ	L	1063482..1063698	PISO0L12529g-PISO0L12551g	100	Pγ	JQ267645
homozygous	gene	K	1749204..1749606	PISO0K21350g	100	Pγ	L	1749204..1749606	PISO0L21351g	100	Pγ	FN556130
homozygous	intergene	K	1462003..1462275	PISO0K17830g-PISO0K17808g	100	Pγ	L	1462003..1462275	PISO0L17831g-PISO0L17809g	100	Pγ	JQ267638
heterozygous	gene	M	169354..169782	PISO0M02058g	96	Pε	N	188531..188959	PISO0N02191g	99	Pγ	FN994867
heterozygous	NUMT p24	M	158164..158608	PISO0M01926g-PISO0M01924g	75	Pε	N	177405..177814	PISO0N02059g-PISO0N02037g	100	Pγ	JQ267634
heterozygous	gene	M	1827135..1827615	PISO0M21550g	95	Pε	N	1837559..1838039	PISO0N21793g	100	Pγ	FN994863

The table indicates the position of selected markers in *P. sorbitophila* genome used in Mallet *et al*. (in preparation) for a taxonomical study of *P. farinosa* species (in blue) and used in these study (in black). These markers are present in two allelic versions (_1 and _2) either at a heterozygous or at a homozygous state (column "region").They correspond to part of genes, intergenes or NUMTs (column "feature"). Position of each allele is indicated(*). These markers were used for PCR amplifications and sequencing in *P. farninosa* CBS 2001 using oligonucleotides that hybridized on both alleles. Identity between the obtained *P. farinosa* sequences and each allele of *P. sorbitophila* are indicated ($). According to the sequence identities shared between *P. farinosa* and *P. sorbitophila* alleles, the belonging of each allele into one parental subgenome Pγ or Pε is proposed (£). These markers are plotted on Figure 1

**corresponding sequence : GATAATTATGATGTGTAAATCGAACTTGGAAATTCAACAAATACTGCAATTTTTTTCTACTCTTTTTTTCCAACTAGGACACTTCTTGGGCACAATG

Table S5 Databases for yeast species used in this study

species	strain	web site	Reference
Debaryomyces hansenii	CBS767	http://www.genolevures.org/deha.html#	The Génolevures Consortium, 2009 (Souciet *et al.*)
Yarrowia lipolytica	E150	http://www.genolevures.org/yali.html#	The Génolevures Consortium, 2009 (Souciet *et al.*)
Candida albicans	SC5314	http://www.candidagenome.org/download/sequence/Assembly21/current/	Jones *et al.*, 2004, Skrzypek *et al.*, 2009
Candida guilliermondii	ATCC6260	http://www.broadinstitute.org/annotation/genome/candida_albicans/MultiHome.html	Butler *et al.*, 2009
Candida lusitaniae	ATCC 42720	http://www.broadinstitute.org/annotation/genome/candida_albicans/MultiHome.html	Butler *et al.*, 2009
Candida parapsilosis	CDC 317	http://www.broadinstitute.org/annotation/genome/candida_albicans/MultiHome.html	Butler *et al.*, 2009
Candida tropicalis	MYA-3404	http://www.broadinstitute.org/annotation/genome/candida_albicans/MultiHome.html	Butler *et al.*, 2009
Lodderomyces elongisporus	NRRL YB-4239	http://www.broadinstitute.org/annotation/genome/candida_albicans/MultiHome.html	Butler *et al.*, 2009
Pichia pastoris	GS115	https://bioinformatics.psb.ugent.be/gdb/pichia/	Mattanovich *et al.*, 2009
Pichia stipitis	CBS 6054	http://genome.jgi-psf.org/Picst3/Picst3.home.html	Jeffries *et al.*, 2007
Candida dubliniensis	CD36	http://www.sanger.ac.uk/sequencing/Candida/dubliniensis/	Jackson *et al.*, 2009

Table S6 Distribution of introns in protein-coding genes

Chromosome	Intron-containing genes					Introns
	1 intron	2 introns	3 introns	4 introns	Total	
Piso0A	30	2	1	1	34	41
Piso0B	31	2	1	1	35	42
Piso0C	47	0	0	0	47	47
Piso0D	47	0	0	0	47	47
Piso0E	35	0	1	1	37	42
Piso0F	42	0	1	1	44	49
Piso0G	39	3	0	0	42	45
Piso0H	39	3	0	0	42	45
Piso0I	65	2	0	0	67	69
Piso0J	73	2	0	0	75	77
Piso0K	57	4	1	1	63	72
Piso0L	57	4	1	1	63	72
Piso0M	61	8	0	0	69	77
Piso0N	62	8	0	0	70	78
Genome	685	38	6	4	735	803

Table S7 Comparison of *P. sorbitophila* global genome features with other yeasts

	ploidy	Genome size (Mb)	Number of chromosomes	average GC content (%)	total CDS	average gene density (%)&	GC in cds (%)	average CDS size&
P. sorbitophila	*hybrid*	21.5	14	41.4	11,252	76.9 (74.5-78.1%)	42	1,476
P. Sorbitophila	*eq. haploid $*	10.75	7	-	5,736	-	-	-
D. hansenii	*haploid*	12.2	7	36.3	6,395	74.2	38.0	1,440
C. guilliermondii	*haploid*	10.6	8	43.8	5,920	-	-	1,402
P. stipitis	*haploid*	15.4	8	41.1	5,841	-	-	-
C. albicans	*diploid*	14.3*	8	33.4	6,107	-	-	1,468
C. tropicalis	*diploid*	14.5*	8	33.1	6,258	-	-	1,454
L. elongisporus	*diploid*	15.4*	8	37.0	5,802	-	-	1,530
C. parapsilosis	*diploid*	13.1*	8	38.7	5,733	-	-	1,533
C. lusitaniae	*haploid*	12.1	8	44.5	5,941	-	-	1,382
Y. lipolytica	*haploid*	20.5	6	49.0	6,580	46.0	53.8	1,470
S. cerevisiae £	*haploid*	12.1	16	38.3	5,769	70.0	40.3	1,467

Genome features of *P. sorbitophila* were compared with other yeasts of the CTG group, with *Yarrowia lipolytica* (*Dipodascaceae* group) and *Saccharomyces cerevisiae* (*Saccharomycetaceae* group). & Average gene density and size is the mean of the values obtained for each chromosome. $ eq. Equivalent in ploidy, total CDS corresponds to identified genes (Supporting Section 2). * Sizes of the haploid genomes given in published data (Table S5 for references) £ The Génolevures Consortium (Souciet *et al.*, 2009)

Table S8   Characteristics of tandemly duplicated gene arrays

species	Nbr. of TGA		total nbr. of CDSs	Nbr. of CDSs in TGA	proportion of CDSs in TGA (%)	Nbr. of TGA in direct orientation	Nbr. of TGA in convergent orientation	Nbr. of TGA in divergent orientation	Nbr. of TGA in mixte orientation
	total	with relic							
Saccharomyces cerevisiae	52	3	5859	111	1.895	42 (80.8)	5 (9.6)	4 (7.7)	1 (1.9)
Candida glabrata	44	1	5200	114	2.192	31 (70.5)	6 (13.6)	3 (6.8)	4 (9.1)
Zygosaccharomyces rouxii	47	8	4991	97	1.943	41 (87.2)	3(6.4)	2 (4.3)	1 (2.1)
Kluyveromyces thermotolerans	37	6	5092	77	1.512	30 (81.1)	4 (10.8)	2 (5.4)	1 (2.7)
Kluyveromyces lactis	36	2	5075	72	1.419	27 (75.0)	4 (11.1)	4 (11.1)	1 (2.8)
Debaryomyces hansenii	128	19	6264	273	4.358	110 (85.9)	8 (6.3)	9 (7.0)	1 (0.8)
Pichia sorbitophila hybrid	**75**	**2**	**11252**	**153**	**1.359**	**51 (68.0)**	**4 (5.3)**	**18 (24.0)**	**2 (2.7)**
eq. haploid*	**38**	**1**	**5736**	**77**	**1.359**	**25**	**2**	**9**	**1**
Yarrowia lipolytica	43	7	6426	80	1.245	31 (72.1)	4 (9.3)	8 (18.6)	0 (0.0)

*The number of TGA and genes identified in *P. sorbitophila* genome (hybrid) are converted into an equivalent haplotype for a best comparison with the other haploid hemiascomycetous yeasts described.

Numbers in brackets indicate the percentage of each orientation

Table S9 Gene ontology categories for conserved alleles

Molecular_function	Other_genes	Conserved_alleles	Freq. for other genes	Freq. for conserved_alleles	Freq conserved/Freq other
signal transducer activity	56	4	0.01	0.04	5.43
nucleotidyltransferase activity	114	6	0.01	0.06	4.00
structural molecule activity	470	22	0.06	0.21	3.56
RNA binding	328	14	0.04	0.13	3.24
DNA binding	427	12	0.05	0.12	2.14
transcription regulator activity	389	10	0.05	0.10	1.95
peptidase activity	211	4	0.03	0.04	1.44
hydrolase activity	1343	14	0.17	0.13	0.79
protein binding	786	6	0.10	0.06	0.58
ligase activity	286	2	0.04	0.02	0.53
transferase activity	1179	8	0.15	0.08	0.52
transporter activity	686	2	0.09	0.02	0.22
oxidoreductase activity	516	0	0.07	0.00	0.00
lyase activity	148	0	0.02	0.00	0.00
protein kinase activity	206	0	0.03	0.00	0.00
motor activity	18	0	0.00	0.00	0.00
enzyme regulator activity	307	0	0.04	0.00	0.00
lipid binding	126	0	0.02	0.00	0.00
helicase activity	116	0	0.01	0.00	0.00
translation regulator activity	8	0	0.00	0.00	0.00
isomerase activity	99	0	0.01	0.00	0.00
phosphoprotein phosphatase activity	86	0	0.01	0.00	0.00
Total	7905	104	1.00	1.00	1.00
**Cellular_component**	**Other_genes**	**Conserved_alleles**	**Freq. for other genes**	**Freq. for conserved_alleles**	**Freq conserved/Freq other**
ribosome	488	22	0.03	0.10	3.69
chromosome	444	16	0.02	0.07	2.95
cellular bud	267	8	0.01	0.04	2.45
nucleolus	410	12	0.02	0.06	2.39
site of polarized growth	344	8	0.02	0.04	1.90

Golgi apparatus	316	6	0.02	0.03	1.55
peroxisome	108	2	0.01	0.01	1.51
mitochondrial envelope	506	8	0.03	0.04	1.29
cytoskeleton	265	4	0.01	0.02	1.23
nucleus	2761	38	0.15	0.17	1.13
cell cortex	180	2	0.01	0.01	0.91
cytoplasmic membrane-bounded vesicle	193	2	0.01	0.01	0.85
cytoplasm	5420	52	0.30	0.24	0.78
mitochondrion	1746	16	0.10	0.07	0.75
plasma membrane	486	4	0.03	0.02	0.67
membrane fraction	290	2	0.02	0.01	0.56
membrane	1863	12	0.10	0.06	0.53
vacuole	365	2	0.02	0.01	0.45
endoplasmic reticulum	614	2	0.03	0.01	0.27
cell wall	96	0	0.01	0.00	0.00
microtubule organizing center	78	0	0.00	0.00	0.00
endomembrane system	553	0	0.03	0.00	0.00
extracellular region	41	0	0.00	0.00	0.00
Total	17834	218	1.00	1.00	1.00

See Figure S8 for method

Table S10 Hypervariable alleles in heterozygous regions

type_1	locus_1	lenght_1	orient.	Orthologs*	%idADN	%idProt	dN/dS	type_2	locus_2	lenght_2	orient.
CDS	PISO0C04424g	867	1	1	85,2	77,1	0,4097	CDS	PISO0D04535g	867	1
CDS	PISO0E04294g	834	-1	1	83,3	80,8	0,3954	CDS	PISO0F05703g	738	-1
CDS	PISO0M22232g	264	-1	1	86,4	80,5	0,344	CDS	PISO0N22475g	264	-1
CDS	PISO0C01894g	1527	-1	1	84,7	78	0,4229	CDS	PISO0D01983g	1530	-1
CDS	PISO0C01124g	1734	1	1	86,1	79,5	0,3492	CDS	PISO0D01213g	1731	1
CDS	PISO0C00486g	576	-1	1	92,4	89,5	0,4371	CDS	PISO0D00575g	576	-1
CDS	PISO0C00420g	1041	-1	1	90,7	87,6	0,3452	CDS	PISO0D00509g	1050	-1
CDS	PISO0E00576g	2349	1	1	84,6	79,8	0,3193	CDS	PISO0I00404g	2292	1
CDS	PISO0I18708g	1089	-1	1	92,7	90,6	0,3115	CDS	PISO0J20469g	1089	-1
CDS	PISO0M24828g	2454	-1	1	83,4	75,2	0,3677	CDS	PISO0N25159g	2454	-1
CDS	PISO0I04078g	930	1	1	90,5	87,1	0,3178	CDS	PISO0J05883g	930	1
CDS	PISO0I04188g	450	1	1	90	85,9	0,3182	CDS	PISO0J05993g	450	1
CDS	PISO0A06710g	651	1	1	88,4	84,1	0,3837	CDS	PISO0B06777g	645	1
CDS	PISO0A00660g	561	-1	1	88,4	80,1	0,7127	CDS	PISO0B00727g	561	-1
CDS	PISO0M20406g	318	1	1	92,1	86,7	0,4538	CDS	PISO0N20649g	318	1
CDS	PISO0M21374g	672	1	1	86,6	77,3	0,5284	CDS	PISO0N21617g	663	1
CDS	PISO0M15170g	2325	1	1	86	79,3	0,3576	CDS	PISO0N15567g	2388	1
CDS	PISO0E11620g	2007	-1	1	85,9	78,4	0,403	CDS	PISO0F13007g	2016	-1
CDS	PISO0E11422g	375	-1	1	91,7	87,1	0,7301	CDS	PISO0F12809g	375	-1
CDS	PISO0E11312g	453	-1	1	84,3	74	0,3748	CDS	PISO0F12699g	453	-1
CDS	PISO0I10678g	1575	-1	1	89,7	86,5	0,3403	CDS	PISO0J12483g	1575	-1
CDS	PISO0I10612g	609	-1	1	92,4	90,6	0,481	CDS	PISO0J12439g	621	-1
CDS	PISO0I06630g	1044	1	1	86,3	80,7	0,3736	CDS	PISO0J08457g	1047	1
CDS	PISO0M02740g	549	-1	1	88,2	84,1	0,312	CDS	PISO0N02873g	561	-1
CDS	PISO0E07198g	627	-1	1	89,5	84,1	0,3981	CDS	PISO0F08541g	627	-1
CDS	PISO0E07814g	1293	-1	1	87,9	82,3	0,4013	CDS	PISO0F09157g	1290	-1
CDS	PISO0E08518g	2262	-1	1	86,9	80,5	0,401	CDS	PISO0F09861g	2253	-1
CDS	PISO0A03190g	276	-1	1	89,5	84,6	0,3256	CDS	PISO0B03301g	276	-1
CDS	PISO0I02054g	2067	1	1	84,3	75,4	0,3875	CDS	PISO0J03837g	2064	1
CDS	PISO0M20890g	675	-1	1	86,4	81,2	0,3232	CDS	PISO0N21133g	675	-1

CDS	PISO0M00474g	912	1	1	83,4	75,5	0,3734	CDS	PISO0N00629g	909	1
CDS	PISO0M14400g	390	-1	1	92,8	88,4	0,417	CDS	PISO0N14797g	390	-1
CDS	PISO0M12706g	354	-1	1	91,8	89,7	0,3258	CDS	PISO0N13015g	354	-1
CDS	PISO0F01237g	819	1	1	88	83,1	0,4351	CDS	PISO0J01219g	819	1
CDS	PISO0I14484g	4455	1	1	88,7	85,3	0,313	CDS	PISO0J16289g	4458	1
CDS	PISO0M11870g	831	-1	1	91,5	88,4	0,3338	CDS	PISO0N12179g	831	-1
CDS	PISO0M13212g	1101	1	1	88,6	84,3	0,4086	CDS	PISO0N13521g	1035	1
CDS	PISO0E13578g	680	-1	0	97,3	96,2	0,4647	CDS	PISO0F14965g	693	-1
CDS	PISO0F01567g	330	1	0	75,8	38,9	1,1931	CDS	PISO0J01549g	273	1
CDS	PISO0M10440g	225	1	0	83,9	68,3	1,1526	CDS	PISO0N10727g	192	1
CDS	PISO0C00992g	339	-1	0	93	76,8	1,0331	CDS	PISO0D01081g	300	-1
CDS	PISO0E06032g	339	-1	1	93,2	91,1	0,6224	CDS	PISO0F07353g	342	-1
CDS	PISO0I05816g	2091	-1	0	80	68,7	0,6116	CDS	PISO0J07599g	2220	-1
CDS	PISO0I12636g	768	-1	1	89,2	82	0,4956	CDS	PISO0J14463g	753	-1
CDS	PISO0A00946g	642	1	0	85	75,6	0,4882	CDS	PISO0B01101g	642	1
CDS	PISO0C04006g	261	1	0	90	67,4	0,479	CDS	PISO0D04095g	273	1
CDS	PISO0M05380g	1008	1	0	83,3	72,2	0,4547	CDS	PISO0N05535g	876	1
CDS	PISO0E11114g	1098	-1	0	90,2	84,7	0,4175	CDS	PISO0F12501g	1119	-1
CDS	PISO0E08892g	1380	1	0	82	70,8	0,4069	CDS	PISO0F10235g	1407	1
CDS	PISO0M05358g	1173	1	0	88,2	82,1	0,4014	CDS	PISO0N05513g	1179	1
CDS	PISO0I05508g	582	-1	1	85,5	83,7	0,3793	CDS	PISO0J07313g	573	-1
CDS	PISO0M11892g	357	-1	1	86,8	80,5	0,3709	CDS	PISO0N12201g	357	-1
CDS	PISO0A05522g	660	1	1	89,4	86,3	0,3517	CDS	PISO0B05589g	660	1
CDS	PISO0E12060g	690	1	1	82,5	78,6	0,5083	CDS	PISO0F13447g	762	1
CDS	PISO0M24542g	1023	-1	1	89,2	86,8	0,3996	CDS	PISO0N24829g	1044	-1
CDS	PISO0C01388g	1743	-1	1	84,2	76,9	0,3699	CDS	PISO0D01477g	1749	-1
CDS	PISO0A04642g	1074	1	1	90,4	85,7	0,3815	CDS	PISO0B04731g	1074	1
CDS	PISO0F01457g	1272	1	1	88,4	82,4	0,3918	CDS	PISO0J01439g	1233	1
CDS	PISO0I10348g	954	1	1	91,8	87,7	0,4854	CDS	PISO0J12175g	954	1
CDS	PISO0I03242g	1185	-1	1	95,6	94,2	0,35	CDS	PISO0J05025g	1185	-1
CDS	PISO0M00672g	1428	-1	1	90,9	88	0,3652	CDS	PISO0N00827g	1404	-1
CDS	PISO0M04060g	363	-1	1	92	78,3	0,403	CDS	PISO0N04193g	375	-1
CDS	PISO0E12808g	1827	-1	1	86,1	78,9	0,4186	CDS	PISO0F14195g	1824	-1
CDS	PISO0I08632g	2853	-1	1	86,7	81,1	0,376	CDS	PISO0J10459g	2859	-1
CDS	PISO0I12394g	2328	-1	1	86,9	80,2	0,4374	CDS	PISO0J14221g	2304	-1
CDS	PISO0E05812g	2379	1	1	82,6	75,6	0,3886	CDS	PISO0F07133g	2220	1

CDS	PISO0E08232g	786	1	1	91,1	86,2	0,7959	CDS	PISO0F09575g	786	1
CDS	PISO0I11074g	3219	1	1	86,5	80,1	0,3858	CDS	PISO0J12879g	3243	1
CDS	PISO0I17058g	495	1	1	95,4	94,5	0,3123	CDS	PISO0J18819g	438	1
CDS	PISO0M04830g	201	1	0	91,5	84,8	0,8357	CDS	PISO0N04941g	201	1
CDS	PISO0M03774g	366	-1	1	83,6	76	0,6206	CDS	PISO0N03907g	384	-1
CDS	PISO0E03128g	1155	-1	0	83,8	73,2	0,506	CDS	PISO0F04537g	1155	-1
CDS	PISO0M14906g	732	1	0	84	75	0,5017	CDS	PISO0N15303g	687	1
CDS	PISO0M14862g	438	1	1	87,8	76,8	0,4723	CDS	PISO0N15259g	378	1
CDS	PISO0M05182g	3654	-1	0	82,2	71,7	0,4399	CDS	PISO0N05337g	3609	-1
CDS	PISO0M00430g	2364	1	0	83,6	75,3	0,4023	CDS	PISO0N00585g	2364	1
CDS	PISO0M10088g	2898	1	0	85,8	78	0,3887	CDS	PISO0N10397g	2898	1
CDS	PISO0M09076g	1413	-1	0	86,7	79,8	0,3842	CDS	PISO0N09275g	1446	-1
CDS	PISO0M13630g	963	-1	1	87	80,3	0,3699	CDS	PISO0N13983g	966	-1
CDS	PISO0I15320g	966	-1	1	90,1	85	0,3694	CDS	PISO0J17125g	966	-1
CDS	PISO0I13142g	1125	1	1	88,4	84,5	0,3428	CDS	PISO0J14947g	1125	1
CDS	PISO0M22122g	369	-1	1	86,2	82	0,328	CDS	PISO0N22365g	372	-1
CDS	PISO0M20296g	303	-1	1	88,4	84	0,3215	CDS	PISO0N20539g	303	-1
CDS	PISO0I15210g	324	1	1	89,5	85	0,3857	CDS	PISO0J17015g	324	1
CDS	PISO0M21264g	945	-1	1	83,4	73,9	0,4148	CDS	PISO0N21507g	933	-1
CDS	PISO0I18796g	1203	1	1	88,5	84	0,3375	CDS	PISO0J20557g	1227	1
CDS	PISO0C01630g	1284	-1	1	89,1	84,3	0,4819	CDS	PISO0D01719g	1308	-1
CDS	PISO0I05442g	399	1	1	91,7	89,4	0,3506	CDS	PISO0J07247g	399	1
CDS	PISO0E01654g	498	-1	1	85,1	79,4	0,4916	CDS	PISO0I01482g	498	-1
CDS	PISO0M20098g	1374	-1	1	86,5	78,6	0,4618	CDS	PISO0N20341g	1386	-1

Both alleles of a gene (_1 and _2) are on the same line. They were compared for their identity in DNA (%idADN), in protein (%idProt), for the dN/dS ratio and the presence of at least one ortholog in the CTG group* (if yes=1). Percentages of identity were calculated for the aligned part of the sequences

Table S11 Gene ontology categories for hypervariable alleles

Molecular_function	Other_genes	Hypervariable_alleles	Freq. for other genes	Freq. for hypervariable alleles	Freq hypervariable/Freq other
phosphoprotein phosphatase activity	84	2	0.01	0.05	4.87
structural molecule activity	486	6	0.06	0.15	2.52
isomerase activity	98	1	0.01	0.03	2.09
DNA binding	435	4	0.05	0.10	1.88
hydrolase activity	1347	10	0.17	0.26	1.52
ligase activity	286	2	0.04	0.05	1.43
enzyme regulator activity	305	2	0.04	0.05	1.34
RNA binding	340	2	0.04	0.05	1.20
protein binding	788	4	0.10	0.10	1.04
oxidoreductase activity	514	2	0.06	0.05	0.80
transferase activity	1183	4	0.15	0.10	0.69
signal transducer activity	60	0	0.01	0.00	0.00
peptidase activity	215	0	0.03	0.00	0.00
lyase activity	148	0	0.02	0.00	0.00
transcription regulator activity	399	0	0.05	0.00	0.00
protein kinase activity	206	0	0.03	0.00	0.00
motor activity	18	0	0.00	0.00	0.00
transporter activity	688	0	0.09	0.00	0.00
lipid binding	126	0	0.02	0.00	0.00
helicase activity	116	0	0.01	0.00	0.00
translation regulator activity	8	0	0.00	0.00	0.00
nucleotidyltransferase activity	120	0	0.02	0.00	0.00
Total	7970	39	1.00	1.00	1.00

Cellular_component	Other_genes	Hypervariable_alleles	Freq. for other genes	Freq. for hypervariable alleles	Freq hypervariable/Freq other
extracellular region	37	4	0.00	0.03	16.72
cell wall	90	6	0.01	0.05	10.31
microtubule organizing center	76	2	0.00	0.02	4.07
endoplasmic reticulum	606	10	0.03	0.09	2.55
endomembrane system	545	8	0.03	0.07	2.27
membrane fraction	288	4	0.02	0.03	2.15
Ribosome	504	6	0.03	0.05	1.84

cell cortex	180	2	0.01	0.02	1.72
Vacuole	363	4	0.02	0.03	1.70
Nucleolus	418	4	0.02	0.03	1.48
Cytoskeleton	267	2	0.01	0.02	1.16
cellular bud	273	2	0.02	0.02	1.13
Nucleus	2779	20	0.15	0.17	1.11
Golgi apparatus	320	2	0.02	0.02	0.97
Cytoplasm	5440	32	0.30	0.28	0.91
site of polarized growth	350	2	0.02	0.02	0.88
Membrane	1871	4	0.10	0.03	0.33
Mitochondrion	1760	2	0.10	0.02	0.18
Peroxisome	110	0	0.01	0.00	0.00
Chromosome	460	0	0.03	0.00	0.00
cytoplasmic membrane-bounded vesicle	195	0	0.01	0.00	0.00
plasma membrane	490	0	0.03	0.00	0.00
mitochondrial envelope	514	0	0.03	0.00	0.00
Total	17936	116	1.00	1.00	1.00

See Figure 8 for method

Table S12   Gene ontology categories for pseudogenes-CDS pairs of genes

Table S12. Gene ontology categories for pseudogenes-CDS pairs of genes

Molecular_function	Other_genes	Pseudogenes	Freq. for other genes	Freq. for pseudogenes	Freq pseudo/Freq other
oxidoreductase activity	514	2	0.06	0.40	6.23
transporter activity	686	2	0.09	0.40	4.67
transcription regulator activity	398	1	0.05	0.20	4.02
signal transducer activity	60	0	0.01	0.00	0.00
DNA binding	439	0	0.05	0.00	0.00
peptidase activity	215	0	0.03	0.00	0.00
lyase activity	148	0	0.02	0.00	0.00
structural molecule activity	492	0	0.06	0.00	0.00
transferase activity	1187	0	0.15	0.00	0.00
protein kinase activity	206	0	0.03	0.00	0.00
motor activity	18	0	0.00	0.00	0.00
hydrolase activity	1357	0	0.17	0.00	0.00
enzyme regulator activity	307	0	0.04	0.00	0.00
lipid binding	126	0	0.02	0.00	0.00
helicase activity	116	0	0.01	0.00	0.00
translation regulator activity	8	0	0.00	0.00	0.00
ligase activity	288	0	0.04	0.00	0.00
nucleotidyltransferase activity	120	0	0.01	0.00	0.00
isomerase activity	99	0	0.01	0.00	0.00
phosphoprotein phosphatase activity	86	0	0.01	0.00	0.00
RNA binding	342	0	0.04	0.00	0.00
protein binding	792	0	0.10	0.00	0.00
Total	8004	5	1.00	1.00	1.00
**Cellular_component**	**Other_genes**	**Pseudogenes**	**Freq. for other genes**	**Freq. for pseudogenes**	**Freq pseudo/Freq other**
plasma membrane	488	2	0.03	0.14	5.28
mitochondrial envelope	513	1	0.03	0.07	2.51
Membrane	1872	3	0.10	0.21	2.06
Mitochondrion	1760	2	0.10	0.14	1.46
Nucleus	2796	3	0.16	0.21	1.38
Cytoplasm	5469	3	0.30	0.21	0.71
endoplasmic reticulum	616	0	0.03	0.00	0.00
site of polarized growth	352	0	0.02	0.00	0.00

Nucleolus	422	0	0.02	0.00	0.00
cell wall	96	0	0.01	0.00	0.00
Peroxisome	110	0	0.01	0.00	0.00
microtubule organizing center	78	0	0.00	0.00	0.00
Chromosome	460	0	0.03	0.00	0.00
Golgi apparatus	322	0	0.02	0.00	0.00
cytoplasmic membrane-bounded vesicle	195	0	0.01	0.00	0.00
endomembrane system	553	0	0.03	0.00	0.00
cellular bud	275	0	0.02	0.00	0.00
Ribosome	510	0	0.03	0.00	0.00
membrane fraction	292	0	0.02	0.00	0.00
Cytoskeleton	269	0	0.01	0.00	0.00
Vacuole	367	0	0.02	0.00	0.00
extracellular region	41	0	0.00	0.00	0.00
cell cortex	182	0	0.01	0.00	0.00
Total	18038	14	1.00	1.00	1.00

See Figure 8 for method

Table S13 Gene ontology categories for single allele genes

Molecular_function	Other_genes	Single_alleles	Freq. for other genes	Freq. for single_alleles	Freq single/Freq other
transporter activity	664	24	0.08	0.39	4.71
oxidoreductase activity	506	10	0.06	0.16	2.58
transcription regulator activity	392	7	0.05	0.11	2.33
DNA binding	435	4	0.05	0.07	1.20
hydrolase activity	1346	11	0.17	0.18	1.06
peptidase activity	214	1	0.03	0.02	0.61
protein binding	790	2	0.10	0.03	0.33
transferase activity	1185	2	0.15	0.03	0.22
signal transducer activity	60	0	0.01	0.00	0.00
lyase activity	148	0	0.02	0.00	0.00
structural molecule activity	492	0	0.06	0.00	0.00
protein kinase activity	206	0	0.03	0.00	0.00
motor activity	18	0	0.00	0.00	0.00
enzyme regulator activity	307	0	0.04	0.00	0.00
lipid binding	126	0	0.02	0.00	0.00
helicase activity	116	0	0.01	0.00	0.00
translation regulator activity	8	0	0.00	0.00	0.00
ligase activity	288	0	0.04	0.00	0.00
nucleotidyltransferase activity	120	0	0.02	0.00	0.00
isomerase activity	99	0	0.01	0.00	0.00
phosphoprotein phosphatase activity	86	0	0.01	0.00	0.00
RNA binding	342	0	0.04	0.00	0.00
Total	7948	61	1.00	1.00	1.00
**Cellular_component**	**Other_genes**	**Single_alleles**	**Freq. for other genes**	**Freq. for single_alleles**	**Freq single/Freq other**
extracellular region	38	3	0.00	0.03	12.00
plasma membrane	471	19	0.03	0.16	6.13
cell wall	94	2	0.01	0.02	3.23
Membrane	1851	24	0.10	0.20	1.97
membrane fraction	289	3	0.02	0.03	1.58
cytoplasmic membrane-bounded vesicle	193	2	0.01	0.02	1.57
Vacuole	364	3	0.02	0.03	1.25
Mitochondrion	1748	14	0.10	0.12	1.22
Ribosome	506	4	0.03	0.03	1.20

Cytoplasm	5442	30	0.30	0.25	0.84
cellular bud	274	1	0.02	0.01	0.55
endoplasmic reticulum	614	2	0.03	0.02	0.50
Nucleus	2790	9	0.16	0.08	0.49
mitochondrial envelope	513	1	0.03	0.01	0.30
endomembrane system	552	1	0.03	0.01	0.28
site of polarized growth	352	0	0.02	0.00	0.00
Nucleolus	422	0	0.02	0.00	0.00
Peroxisome	110	0	0.01	0.00	0.00
microtubule organizing center	78	0	0.00	0.00	0.00
Chromosome	460	0	0.03	0.00	0.00
Golgi apparatus	322	0	0.02	0.00	0.00
Cytoskeleton	269	0	0.01	0.00	0.00
cell cortex	182	0	0.01	0.00	0.00
Total	17934	118	1.00	1.00	1.00

See Figure 8 for method

Table S14 tDNA numbers per chromosome in *P. sorbitophila* genome

chr.	Nbr per chr.			in eq. haploid
	Heterozygous	Homozygous	Total	
A	11	10	21	21
B	11	10	21	
C	6	4	10	10
D	6	4	10	
I (I/E)	0	0	0	0
E (I/E)	0	0	0	
J (J/F)	4	0	4	4
F (J/F)	4	0	4	
E (E/F)	17	0	17	17
F (E/F)	17	0	17	
I (I/J)	25	0	25	25
J (I/J)	25	0	25	
G	0	17	17	17
H	0	17	17	
K	0	25	25	25
L	0	25	25	
M	25	0	25	25
N	25	0	25	
**Total**	**176**	**112**	**288**	**144**

Genes encoding tRNAs were searched with tRNAScan-SE (Lowe and Eddy, 1997) and obtained sequences were analyzed for possible exceptions to the eukaryotic cloverleaf model, as described in Marck and Grosjean (2002). Initiator Met tRNA genes were distinguished from the elongator sequences thanks to the "GGG" sequence in positions 29-31. For chr. E/F/I/J, the syntenic heterozygous pairs considered are mentionned in brackets.

Table S15 Pairs of potentially co-transcribed tRNA genes in *P. sorbitophila*

tDNA pairs and intervals in genome	nb of occurrences	
tDNA-Asp (GTC)-{10 or 11 nt}-tDNA-Gly (GCC)	x4	*
tDNA-Ile (AAT)-{11 or 13 nt}-tDNA-Ala (AGC)	x4	*
tDNA-Asn (GTT)-{9 nt}-tDNA-Thr (AGT)	x1	
tDNA-Val (AAC)-{8 nt}-tDNA-Gly (GCC)	x1	*

This table lists the pairs of neighbouring and co-oriented tRNA genes. The tRNA type and distance between tRNA genes (number of nucleotides between base 73 of the first tRNA gene and base 1 of the following gene) are indicated. These very short intervals suggest that the two tandem tDNAs are co-transcribed (Dujon *et al*., 2004). * Pairs also present in the genome of *Y. lipolytica.*

Table S16 Codon and tRNA gene usages in *P. sorbitophila* and *D. hansenii*

	AA	C	AC	*P.sorbitophila*		*D. hansenii*	
F	Phe	TTT	---	2.27	-	2.64	-
F	Phe	TTC	(GAA)	2.14	5+	1.82	8+
L	Leu	TTA	(TAA)	2.19	2	3.64	9
L	Leu	TTG	(CAA)	3.32	7	3.11	4
L	Leu	CTT	(AAG)	1.77	2	1.21	2
L	Leu	CTC	(GAG)	1.13	-	0.47	-
L	Leu	CTA	(TAG)	0.90	-	0.78	-
S	Ser	CTG	(CAG)	0.66	1	0.41	1
I	Ile	ATT	(AAT)	2.37	6	3.45	9
I	Ile	ATC	(GAT)	1.82	-	1.53	-
I	Ile	ATA	(TAT)	2.15	2+	2.07	2+
M	Met	ATG	(CAT)	1.73	3+	0.21	4+
m	iMet	ATG	(CAT)	0.21	2	1.79	3
V	Val	GTT	(AAC)	1.94	6	2.38	10
V	Val	GTC	---	1.27	-	0.94	-
V	Val	GTA	(TAC)	1.13	1+	1.14	1+
V	Val	GTG	(CAC)	1.41	2	0.96	1
S	Ser	TCT	(AGA)	2.23	5	2.16	6
S	Ser	TCC	---	1.21	-	0.99	-
S	Ser	TCA	(TGA)	1.73	2	2.13	3
S	Ser	TCG	(CGA)	1.27	1	1.31	1+
P	Pro	CCT	(AGG)	1.55	1	1.33	1
P	Pro	CCC	---	0.79	-	0.43	-
P	Pro	CCA	(TGG)	1.48	4+	1.99	7+
P	Pro	CCG	(CGG)	0.45	-	0.49	-
T	Thr	ACT	(AGT)	1.62	5	1.95	8
T	Thr	ACC	---	1.14	-	1.09	-
T	Thr	ACA	(TGT)	1.60	2	1.66	2
T	Thr	ACG	(CGT)	0.84	1	0.78	1
A	Ala	GCT	(AGC)	1.99	6	1.95	7

A	Ala	GCC	---	1.24	-	0.92	-
A	Ala	GCA	(TGC)	1.71	4	1.73	4
A	Ala	GCG	(CGC)	0.77	1	0.56	1
Y	Tyr	TAT		1.77	-	2.24	-
Y	Tyr	TAC	(GTA)	1.76	5+	1.36	6+
*	Och	TAA	---		-		-
*	Amb	TAG	---		-		-
H	His	CAT	---	1.28	-	1.46	-
H	His	CAC	(GTG)	0.91	4	0.63	5
Q	Gln	CAA	(TTG)	1.91	4	2.68	7
Q	Gln	CAG	(CTG)	1.72	2	1.13	1
N	Asn	AAT	---	3.08	-	4.44	-
N	Asn	AAC	(GTT)	2.65	6	2.22	8
K	Lys	AAA	(TTT)	3.47	3+	3.92	7+
K	Lys	AAG	(CTT)	3.82	5+	3.47	10+
D	Asp	GAT	---	3.46	-	4.33	-
D	Asp	GAC	(GTC)	2.59	7	1.85	9
E	Glu	GAA	(TTC)	3.84	4	4.83	9
E	Glu	GAG	(CTC)	2.86	4	1.75	1
C	Cys	TGT	---	0.66	-	0.81	-
C	Cys	TGC	(GCA)	0.53	2	0.35	4
*	Opa	TGA	---		-		-
W	Trp	TGG	(CCA)	1.00	3	0.99	4
R	Arg	CGT	(ACG)	0.59	2+	0.53	4+
R	Arg	CGC	---	0.38	-	0.13	-
R	Arg	CGA	(TCG)	0.39	-	0.27	-
R	Arg	CGG	(CCG)	0.26	1+	0.17	1+
S	Ser	AGT	---	1.35	-	1.53	-
S	Ser	AGC	(GCT)	1.34	3+	0.77	3+
R	Arg	AGA	(TCT)	2.09	5	2.24	9
R	Arg	AGG	(CCT)	0.89	1	0.68	1
G	Gly	GGT	---	1.97	-	2.30	-
G	Gly	GGC	(GCC)	1.10	8	0.69	11

G	Gly	GGA	(TCC)	1.67	3	1.52	4
G	Gly	GGG	(CCC)	0.67	1	0.69	1

The first column (AA) indicates the charged amino acid (in one- and three-letter codes), the second one (C) the codon and the third one (AC) the anticodon found (between brackets). For each genome, the first value is the codon usage (in % of the 61 sense codons) and the second one the number of tRNA genes ("-" no tRNA gene). The "+" signs denote genes with intron. The number of tRNA indicated refers to the theoretical haploid genome. The codon usage refers to the complete diploid genome in order to average the heterozygosity. Data for *Debaryomyces hansenii* are revised data from Dujon *et al*., 2004.

Table S17 List of ncRNA genes

ncRNA class	Gene_name	Allele_1				%identity*	Allele_2			Area**
		Allele1	Subgenome	Locus1	Annotation		Allele2	Subgenome	Locus2	
snRNA	Piso0_001482	Piso0_001482-1	Pε	Piso0E06043r	snRNA SNR19 (U1)	84.6	Piso0_001482-2	Pγ	PISO0F07375r	he
	Piso0_003856	Piso0_003856-1	Pγ	PISO0K04080r	snRNA SNR20 (U2)	100.0	Piso0_003856-1	Pγ	PISO0L04081r	hm
	Piso0_005802	Piso0_005802-1	Pε	PISO0M22672r	snRNA SNR14 (U4)	98.2	Piso0_005802-2	Pγ	PISO0N22915r	he
	Piso0_005825	Piso0_005825-1	Pε	PISO0M23134r	snRNA SNR7 (U5)	98.4	Piso0_005825-2	Pγ	PISO0N23421r	he
snoRNA	Piso0_003217	Piso0_003217-1	Pγ	PISO0G07464r	snoRNA snR5	100.0	Piso0_003217-1	Pγ	PISO0H07465r	hm
	Piso0_004120	Piso0_004120-1	Pγ	PISO0K09888r	snoRNA SNR8	100.0	Piso0_004120-1	Pγ	PISO0L09889r	hm
	Piso0_000301	Piso0_000301-1	Pγ	PISO0A06336r	snoRNA SNR10	98.3	Piso0_000301-2	Pε	PISO0B06403r	he
	Piso0_003392	Piso0_003392-1	Pγ	PISO0G11314r	snoRNA SNR128	100.0	Piso0_003392-1	Pγ	PISO0H11315r	hm
	Piso0_005688	Piso0_005688-1	Pε	PISO0M20164r	snoRNA SNR17	98.9	Piso0_005688-2	Pγ	PISO0N20407r	he
	Piso0_003431	Piso0_003431-1	Pγ	PISO0G12172r	snoRNA SNR18	100.0	Piso0_003431-1	Pγ	PISO0H12173r	hm
	Piso0_000022	Piso0_000022-1	Pγ	PISO0A00440r	snoRNA SNR191	92.5	Piso0_000022-2	Pε	PISO0B00507r	he
	Piso0_004900	Piso0_004900-1	Pε	PISO0M03422r	snoRNA SNR24	100.0	Piso0_004900-1	Pγ	PISO0N03555r	he
	Piso0_000550	Piso0_000550-1	Pε	PISO0A11814r	snoRNA SNR32	100.0	Piso0_000550-1	Pε	PISO0B11881r	hm
	Piso0_002702	Piso0_002702-1	Pγ	PISO0I15936r	snoRNA SNR35	99.0	Piso0_002702-2	Pε	PISO0J17741r	he
	Piso0_005801	Piso0_005801-1	Pε	PISO0M22650r	snoRNA SNR36	95.6	Piso0_005801-2	Pγ	PISO0N22893r	he
	Piso0_005896	Piso0_005896-1	Pε	PISO0M24652r	snoRNA SNR37	97.9	Piso0_005896-2	Pγ	PISO0N24939r	he
	Piso0_001254	Piso0_001254-1	Pε	PISO0E01060r	snoRNA SNR38	91.0	Piso0_001254-2	Pγ	PISO0I00888r	he
	Piso0_005580	Piso0_005580-1	Pε	PISO0M17788r	snoRNA SNR40	100.0	Piso0_005580-1	Pγ	PISO0N18031r	he
	Piso0_005631	Piso0_005631-1	Pε	PISO0M18910r	snoRNA SNR41	97.9	Piso0_005631-2	Pγ	PISO0N19153r	he
	Piso0_002238	Piso0_002238-1	Pγ	PISO0I05871r	snoRNA SNR42	95.6	Piso0_002238-2	Pε	PISO0J07665r	he
	Piso0_001592	Piso0_001592-1	Pε	PISO0E08430r	snoRNA SNR43	94.7	Piso0_001592-2	Pγ	PISO0F09773r	he
	Piso0_002636	Piso0_002636-1	Pγ	PISO0I14506r	snoRNA SNR47	100.0	Piso0_002636-1	Pε	PISO0J16311r	he
	Piso0_003338	Piso0_003338-1	Pγ	PISO0G10126r	snoRNA SNR49	100.0	Piso0_003338-1	Pγ	PISO0H10127r	hm
	Piso0_005632	Piso0_005632-1	Pε	PISO0M18932r	snoRNA SNR51	98.9	Piso0_005632-2	Pγ	PISO0N19175r	he
	Piso0_001150	Piso0_001150-1	Pγ	PISO0C11992r	snoRNA SNR54	100.0	Piso0_001150-1	Pγ	PISO0D12059r	hm
	Piso0_002436	Piso0_002436-1	Pγ	PISO0I10128r	snoRNA SNR55	96.1	Piso0_002436-2	Pε	PISO0J11955r	he
	Piso0_004621	Piso0_004621-1	Pγ	PISO0K20910r	snoRNA SNR56	100.0	Piso0_004621-1	Pγ	PISO0L20911r	hm
	Piso0_002437	Piso0_002437-1	Pγ	PISO0I10150r	snoRNA SNR57	91.3	Piso0_002437-2	Pε	PISO0J11977r	he
	Piso0_002435	Piso0_002435-1	Pγ	PISO0I10106r	snoRNA SNR61	98.9	Piso0_002435-2	Pε	PISO0J11933r	he
	Piso0_000459	Piso0_000459-1	Pε	PISO0A09812r	snoRNA SNR62	100.0	Piso0_000459-1	Pε	PISO0B09879r	hm
	Piso0_001366	Piso0_001366-1	Pε	PISO0E03502r	snoRNA SNR66	93.1	Piso0_001366-2	Pγ	PISO0F04911r	he
	Piso0_005068	Piso0_005068-1	Pε	PISO0M07030r	snoRNA SNR67	98.9	Piso0_005068-2	Pγ	PISO0N07163r	he
	Piso0_005140	Piso0_005140-1	Pε	PISO0M08504r	snoRNA SNR71	96.6	Piso0_005140-2	Pγ	PISO0N08681r	he
	Piso0_003377	Piso0_003377-1	Pγ	PISO0G10984r	snoRNA SNR73	100.0	Piso0_003377-1	Pγ	PISO0H10985r	hm
	Piso0_003376	Piso0_003376-1	Pγ	PISO0G10962r	snoRNA SNR74	100.0	Piso0_003376-1	Pγ	PISO0H10963r	hm

	Piso0_003375	Piso0_003375-1	Pγ	PISO0G10940r	snoRNA SNR75	100.0	Piso0_003375-1	Pγ	PISO0H10941r	hm
	Piso0_003374	Piso0_003374-1	Pγ	PISO0G10918r	snoRNA SNR76	100.0	Piso0_003374-1	Pγ	PISO0H10919r	hm
	Piso0_003373	Piso0_003373-1	Pγ	PISO0G10896r	snoRNA SNR77	100.0	Piso0_003373-1	Pγ	PISO0H10897r	hm
	Piso0_003372	Piso0_003372-1	Pγ	PISO0G10874r	snoRNA SNR78	100.0	Piso0_003372-1	Pγ	PISO0H10875r	hm
	Piso0_005706	Piso0_005706-1	Pε	PISO0M20560r	snoRNA SNR79	98.9	Piso0_005706-2	Pγ	PISO0N20803r	he
	Piso0_000435	Piso0_000435-1	Pε	PISO0A09284r	snoRNA SNR80	100.0	Piso0_000435-1	Pε	PISO0B09351r	hm
Polymerase III transcripts										
snRNA	Piso0_001264	Piso0_001264-1	Pε	PISO0E01280r	SNR6 (U6 RNA)	100.0	Piso0_001264-1	Pγ	PISO0I01108r	he
SRP RNA	Piso0_004927	Piso0_004927-1	Pε	PISO0M04016r	SCR1 (SRP RNA)	98.0	Piso0_004927-2	Pγ	PISO0N04149r	he
RNaseP RNA	Piso0_005211	Piso0_005211-1	Pε	PISO0M09890r	RPR1 (RNase P RNA)	94.4	Piso0_005211-2	Pγ	PISO0N10199r	he
Rnase MRP RNA	Piso0_001365	Piso0_001365-1	Pε	PISO0E03480r	RNase_MRP	93.0	Piso0_001365-2	Pγ	PISO0F04889r	he
snoRNA	Piso0_001993	Piso0_001993-1	Pγ	PISO0F02403r	SNR52	97.1	Piso0_001993-2	Pε	PISO0J02363r	he

Non-coding RNA (ncRNA) genes were detected using the following bioinformatics tools: 1) ncRNA sequences from the Genolevures database (Sherman *et al*., 2009) were used as queries for BLAST (Altschul *et al*., 1990) searches and hits with an e-value smaller than 0.1 were retained for validation. 2) covariance models found in RFam (Griffiths-Jones, 2009) database were used to perform Infernal searches (Nawrocki *et al*., 2009). All hits with an e-value smaller than 0.5 were retained for validation. All retained hits were manually checked. Hits were accepted as candidates if: i) the sequence agrees with known structural features, guiding sequences (for snoRNAs) and conserved sequence motifs for homologous molecules or ii) known synteny. * % of identity between both alleles of a ncRNA gene. ** position in heterozygous (he) or homozygous (hm) regions

Table S18 ***P. sorbitophila*** **genes in osmotic stress**

transporter type	transporter name	locus_1*	Subgenome_1	locus_2*	Subgenome_2
K+ and Na+ efflux	ENA1	PISO0C05656g	Pγ	PISO0D05745g	Pε
	ENA2	-		-	
K+/H+ antiporter	KHA1	PISO0M11650g	Pε	PISO0N11959g	Pγ
Na+/H+ antiporter	NHA1	PISO0I14000g	Pγ	PISO0J15805g	Pε
K+ and Na+ influx	TRK1	PISO0E03788g	Pε	PISO0F05197g	Pγ
	HAK1	PISO0K12792g	Pγ	PISO0L12793g	Pγ
P-Type ATPase	ACU1	PISO0A12650g	Pε	PISO0B12717g	Pε
H+-ATPase	PMA1	PISO0E05438g	Pε	PISO0F06759g	Pγ
	VMA2	PISO0E12390g	Pε	PISO0F13799g	Pγ
	PHO89	-		-	
H+/glycerol symport	STL1	PISO0A00352g	Pγ	PISO0B00419g	Pε
Glycerol	GPD	Piso0I12878g	Pγ	Piso0J14683g	Pε
	Gup1	PISO0A06402g	Pγ	PISO0B06469g	Pε
Intracellular Na+/K+ concentration	HAL1	-		-	
Membrane Pεrmeability to K+	TOK1	PISO0I13780g	Pγ	PISO0J15585g	Pε
Aquaglyceroporin	FPS1	-		-	
Na+ transport to vacuole	NHX1	PISO0C09154g	Pγ	PISO0D09221g	Pγ
Water flux	AQY1	PISO0G17386g	Pγ	PISO0H17387g	Pγ
HOG pathway	SLN1	PISO0A11462g	Pε	PISO0B11529g	Pε
	SHO1	PISO0L19899g	Pγ	PISO0K19898g	Pγ
	YPD1	PISO0G11886g	Pγ	PISO0H11887g	Pγ
	SSK1	PISO0M05886g	Pε	PISO0N06041g	Pγ
	SSK2	PISO0M09142g	Pε	PISO0N09363g	Pγ
	STE20	PISO0E12786g	Pε	PISO0F14173g	Pγ
	STE50	PISO0A09240g	Pε	PISO0B09307g	Pε
	STE11	PISO0A07018g	Pε	PISO0B07085g	Pε
	PBS2	PISO0E12962g	Pε	PISO0F14349g	Pγ
	HOG1	PISO0E11840g	Pε	PISO0F13227g	Pγ
	SKO1	PISO0K05598g	Pγ	PISO0L05599g	Pγ
	HOT1	-		-	
	RCK2	PISO0G15846g	Pγ	PISO0H15847g	Pγ
	MSN2/4	PISO0E05768g	Pε	PISO0F07089g	Pγ
	SMP1	-		-	
	MSN1	-		-	
	SGD1	PISO0G09928g	Pγ	PISO0H09929g	Pγ
	SKN7	PISO0M12882g	Pε	PISO0N13191g	Pγ
	TUP1	PISO0A11044g	Pε	PISO0B11111g	Pε

	SSN6	PISO0J01747g	Pε	PISO0F01787g	Pγ
Calcineurin	CNA1	PISO0K05268g	Pγ	PISO0L05269g	Pγ
	CNB1	PISO0G03218g	Pγ	PISO0H03219g	Pγ
	CMD1	PISO0K04432g	Pγ	PISO0L04433g	Pγ
	RCN1	-		-	
	CRZ1	PISO0E04118g	Pε	PISO0F05527g	Pγ
	PPZ1	PISO0K14706g	Pγ	PISO0L14707g	Pγ
	HAL3	PISO0I00778g	Pγ	PISO0E00950g	
	HAL4	PISO0E07858g	Pε	PISO0F09201g	Pγ
stress respons and nutrient-controlled signalling	SNF1	PISO0M19570g	Pε	PISO0N19813g	Pγ
	SNF4	PISO0M12244g	Pε	PISO0N12553g	Pγ
	GAL83	PISO0E03392g	Pε	PISO0F04801g	Pγ
	MLG1	PISO0A10670g	Pε	PISO0B10737g	Pε
	SD1	PISO0I19610g	Pγ	PISO0J21371g	Pε
	MTH1	-		-	
	SNF3	PISO0C01740g	Pε	PISO0D01829g	Pγ
	SNF12	PISO0A11528g	Pε	PISO0B11595g	Pε
	SNF2	PISO0C11310g	Pγ	PISO0D11377g	Pγ
	TOR1/2	PISO0G15538g	Pγ	PISO0H15539g	Pγ
	GCN4	PISO0C02158g	Pε	PISO0D02247g	Pγ
	RIM101	PISO0J01571g	Pε	PISO0F01589g	Pγ
	GLN3	PISO0A01408g	Pγ	PISO0B01585g	Pε
	GAT1	PISO0A00814g	Pγ	PISO0B00881g	Pε
	GAT2	PISO0N06899g	Pγ	PISO0M06766g	Pε

* Locus_1 and _2 indicated the locus name of allele in one chromosome forming a pair.

Table S19   Conservation of mating and meiosis genes¥

	S. cerevisiae	*D. hansenii**	*D. hansenii_2***	*P.sorbitophila_1*	*P.sorbitophila_2*
**Mating**					
a-factor	YDR461W/YNL145W	No	No	No	No
alpha-factor	YPL187W/YGL089C	DEHA0F20900g	DEHA2F19580g	PISO0G09378g	PISO0H09379g
RAM2	YKL019W	DEHA0F12034g	DEHA2F11022g	PISO0K09932g	PISO0L09933g
KEX1	YGL203C	yes	DEHA2F22352g	PISO0N03137g	PISO0M02982g
KEX2	YNL238W	DEHA0C11308g	DEHA2C10296g	PISO0G11380g	PISO0H11381g
STE13	YOR219C	DEHA0G24486g	DEHA2G23144g	PISO0I16002g	PISO0J17807g
STE23	YLR389C	DEHA0A05236g	DEHA2A05214g	PISO0D03589g	PISO0C03500g
RCE1	YMR274C	DEHA0F08041g	DEHA2F07260g	PISO0K06126g	PISO0L06127g
STE24	YJR117W	DEHA0F06820g	DEHA2F06248g	PISO0I14264g	PISO0J16069g
RAM1	YDL090C	DEHA0G18007g	DEHA2G16918g	PISO0G06122g	PISO0H06123g
STE14	YDR410C	DEHA0G06017g	DEHA2G05368g	PISO0C09066g	PISO0D09133g
STE6	YKL209C	DEHA0F18667g	DEHA2F17226g	PISO0I06388g	PISO0J08193g
STE2	YFL026W	DEHA0A11110g	DEHA2A10736g	PISO0F04823g	PISO0E03414g
STE3	YKL178C	DEHA0D04708g	DEHA2D04004g	PISO0I04254g	PISO0J06059g
GPA1	YHR005C	DEHA0D14894g	DEHA2D13618g	PISO0K01594g	PISO0L01595g
SST2	YLR452C	DEHA0E10692g	DEHA2E10164g	PISO0K11252g	PISO0L11253g
STE4	YOR212W	DEHA0C17600g	DEHA2C16368g	PISO0K21680g	PISO0L21681g
STE5	YDR103W	DEHA0G13684g	DEHA2G12826g	PISO0F10873g	PISO0E09508g
STE18	YJR086W	yes	DEHA2G24024g	PISO0I16882g	PISO0J18643g
STE20	YHL007C	DEHA0E23529g	DEHA2E22220g	PISO0F14173g	PISO0E12786g
STE11	YLR362W	DEHA0B04895g	DEHA2B05016g	PISO0A07018g	PISO0B07085g
STE7	YDL159W	DEHA0F15719g	DEHA2F14498g	PISO0I08896g	PISO0J10723g
STE12	YHR084W	DEHA0F27445g	DEHA2F25894g	PISO0N06877g	PISO0M06744g
FAR1	YJL157C	DEHA0F14905g	DEHA2F13728g	PISO0I09622g	PISO0J11427g
STE50	YCL032W	DEHA0D19019g	DEHA2D17490g	PISO0A09240g	PISO0B09307g
DIG1/2	YDR480W	DEHA0D17083g	DEHA2D15686g	PISO0I01196g	PISO0E01368g
CDC24	YAL041W	DEHA0E12452g	DEHA2E11836g	PISO0N16799g	PISO0M16402g
CDC42	YLR229C	DEHA0G15037g	DEHA2G14168g	PISO0F12083g	PISO0E10718g
BEM1	YBR200W	DEHA0E22924g	DEHA2E21670g	PISO0F12523g	PISO0E11136g
FUS3	YBL016W	DEHA0E04290g	DEHA2E03586g	PISO0K16774g	PISO0L16775g
		DEHA0E21219g	DEHA2E20086g	PISO0N15083g	PISO0M14686g
BNI1	YNL271C	DEHA0E14938g	DEHA2E14366g	PISO0N19065g	PISO0M18822g
PFY1	YOR122C	DEHA0G15807g	DEHA2G14784g	PISO0G07794g	PISO0H07795g
ACT1	YFL039C	DEHA0D06193g	DEHA2D05412g	PISO0A06226g	PISO0B06293g
BUD6	YLR319C	DEHA0C12034g	DEHA2C11022g	PISO0G12018g	PISO0H12019g

SPA2	YLL021W	DEHA0E17325g	DEHA2E16588g	PISO0N21199g	PISO0M20956g
PEA2	YER149C	DEHA0G12474g	DEHA2G11792g	PISO0F09883g	PISO0E08540g
AXL1	YPR122W	DEHA0E03179g	DEHA2E02464g	PISO0A01364g	PISO0B01541g
BIM1	YER016W	DEHA0D15554g	DEHA2D14278g	PISO0N01619g	PISO0M01464g
HO	YDL227C	No	No	No	No
KAR1	YNL188W	No	No	No	No
KAR2	YJL034W	DEHA0A01749g	DEHA2A01364g	PISO0F00489g	PISO0J00471g
KAR3	YPR141C	DEHA0B02068g	DEHA2B02068g	PISO0N23795g	PISO0M23530g
KAR4	YCL055W	DEHA0E24156g	DEHA2E22770g	PISO0N08659g	PISO0M08482g
KAR5	YMR065W	DEHA0E22396g	DEHA2E21208g	PISO0F12963g	PISO0E11576g
KAR9	YPL269W	DEHA0D05797g	DEHA2D05016g	PISO0A06578g	PISO0B06645g
**Meiosis**					
CDC27	YBL084C	DEHA0F21934g	DEHA2F20614g	PISO0G10456g	PISO0H10457g
APC4	YDR118W	DEHA0B08327g	DEHA2B08316g	PISO0G03702g	PISO0H03703g
CDC16	YKL022C	DEHA0C07876g	DEHA2C07062g	PISO0I11602g	PISO0J13429g
APC1	YNL172W	DEHA0D09867g	DEHA2D08932g	PISO0K05862g	PISO0L05863g
APC5	YOR249C	DEHA0C09548g	DEHA2C08668g	PISO0N09099g	PISO0M08900g
CDC23	YHR166C	DEHA0E02805g	DEHA2E02156g	PISO0A01122g	PISO0B01277g
CDC26	YFR036W	No	No	No	No
CDH1	YGL003C	DEHA0A06963g	DEHA2A06864g	PISO0C05018g	PISO0D05107g
CDC20	YGL116W	DEHA0E22132g	DEHA2E20966g	PISO0F13205g	PISO0E11818g
SWM1	YDR260C	DEHA0G13827g	DEHA2G12958g	PISO0F10983g	PISO0E09618g
CDC10	YCR002C	DEHA0F18557g	DEHA2F17116g	PISO0I06476g	PISO0J08303g
CDC3	YLR314C	DEHA0D13794g	DEHA2D12540g	PISO0K02518g	PISO0L02519g
CDC14	YFR028C	DEHA0F16632g	DEHA2F15356g	PISO0I07972g	PISO0J09799g
CDC5	YMR001C	DEHA0F10923g	DEHA2F09966g	PISO0K08832g	PISO0L08833g
CLB1/2 (WGD gene pair)		DEHA0A02189g	DEHA2A01760g	PISO0F00797g	PISO0J00779g
CLB3/4 (WGD gene pair)		DEHA0G25520g	DEHA2G24134g	PISO0I17014g	PISO0J18775g
CLB5/6 (WGD gene pair)		No	No	No	No
IME1	YJR094C	No	No	No	No
IME2	YJL106W	DEHA0B00770g	DEHA2B00748g	PISO0N24499g	PISO0M24212g
IME4	YGL192W	DEHA0B04491	DEHA2B04598g	PISO0A07392g	PISO0B07459g
IDS2	YJL146W	No	No	No	No
RIM4	YHL024W	DEHA0F03850g	DEHA2F03476g	PISO0A10582g	PISO0B10649g
RME1	YGR044C	DEHA0F21098g	DEHA2F19778g	PISO0G09554g	PISO0H09555g
NDT80	YHR124W	DEHA0A07392g	DEHA2A07282g	PISO0C05414g	PISO0D05503g
		DEHA0F22594g	DEHA2F21230g	PISO0N04215g	PISO0M04082g
SUM1	YDR310C	No	No	No	No
RIM11	YMR139W	DEHA0F09647g	DEHA2F08756g	PISO0K07556g	PISO0L07557g
RIM13	YMR154C	DEHA0G22297g	DEHA2G20988g	PISO0A02486g	PISO0B02597g
RIM15	YFL033C	DEHA0F24013g	DEHA2F22572g	PISO0N02983g	PISO0M02850g
RIM101	YHL027W	DEHA0D05577g	DEHA2D04796g	PISO0F01589g	PISO0J01571g

UME3	YNL025C	DEHA0A10131g	DEHA2A09878g	PISO0F05615g	PISO0E04206g
UME6	YDR207C	DEHA0G16236g	DEHA2G15202g	PISO0N20869g	PISO0M20626g
		DEHA0E16929g	DEHA2E16236g	PISO0N20869g	PISO0M20626g
MCK1	YNL307C	DEHA0E05819g	DEHA2E05060g	PISO0K15630g	PISO0L15631g
SPO12/BNS1 (WGD gene pair)	YHR152W	No	No	No	No
SPO11	YPR007C	DEHA0B13156g	DEHA2B13112g	PISO0D02621g	PISO0C02554g
SPO13	YHR014W	No	No	No	No
SPO22	YIL073C	No	No	No	No
REC102	YLR329W	DEHA0A06699g	DEHA2A06622g	PISO0C04754g	PISO0D04865g
MRE11	YMR224C	DEHA0F17116g	DEHA2F15818g	PISO0I07598g	PISO0J09425g
RAD50	YNL250W	DEHA0D18799g	DEHA2D17314g	PISO0A09042g	PISO0B09109g
RAD1	YPL022W	DEHA0F09823g	DEHA2F08932g	PISO0K07710g	PISO0L07711g
MUS81	YDR386W	DEHA0G05852g	DEHA2G05214g	PISO0C09220g	PISO0D09287g
MER3	YGL251C	DEHA0F08877g	DEHA2F07986g	PISO0A01892g	PISO0B02069g
RAD54	YGL163C	DEHA0D06248g	DEHA2D05456g	PISO0A06182g	PISO0B06249g
MLH1	YMR167W	DEHA0G23320g	DEHA2G22022g	PISO0A03410g	PISO0B03565g
MLH2	YLR035C	No	No	No	No
MLH3	YPL164C	DEHA0E11066g	DEHA2E10472g	PISO0F15295g	PISO0E13908g
PMS1	YNL082W	DEHA0A11297g	DEHA2A10868g	PISO0F04713g	PISO0E03304g
REC8	YPR007C	DEHA0D07843g	DEHA2D07106g	PISO0A04884g	PISO0B04973g
REC114	YMR133W	No	No	No	No
CDC31	YOR257W	DEHA0F16082g	DEHA2F14784g	PISO0I08654g	PISO0J10481g
CSM1	YCR086W	DEHA0A13904g	DEHA2A13486g	PISO0A11132g	PISO0B11199g
CSM3	YMR048W	DEHA0F05522g	DEHA2F05038g	PISO0I13120g	PISO0J14925g
SMC1	YFL008W	DEHA0F21296g	DEHA2F20020g	PISO0G09884g	PISO0H09885g
SMC2	YFR031C	DEHA0B15268g	DEHA2B15136g	PISO0D01059g	PISO0C00970g
SMC3	YJL074C	DEHA0G01958g	DEHA2G01606g	PISO0N02037g	PISO0M01904g
SMC4	YLR086W	No	**DEHA2B07920g**	**PISO0N13059g**	**PISO0M12750g**
SMC5	YOL034W	No	**DEHA2A12606g**	**PISO0F03261g**	**PISO0J03221g**
SMC6	YLR383W	DEHA0A05346g	DEHA2A05324g	PISO0D03721g	PISO0C03632g
SCC1	YDL003W	DEHA0A14476g	DEHA2A14058g	PISO0N22189g	PISO0M21946g
SCC3	YIL026C	DEHA0D05203g	DEHA2D04488g	PISO0F01303g	PISO0J01285g
PDS5	YMR076C	DEHA0F20966g	DEHA2F19646g	PISO0G09444g	PISO0H09445g
RAD51	YER095W	DEHA0C17952g	DEHA2C16698g	PISO0K22098g	PISO0L22099g
RAD52	YML032C	DEHA0G05786g	DEHA2G05148g	PISO0C09264g	PISO0D09331g
RAD55	YDR076W	No	No	No	No
RAD57	YDR004W	DEHA0B13068g	DEHA2B13024g	PISO0D02687g	PISO0C02620g
REC104	YHR157W	No	No	No	No
DMC1	YER179W	DEHA0E17479g	DEHA2E16742g	PISO0N21353g	PISO0M21110g
MND1	YGL183C	DEHA0E04994g	DEHA2E04246g	PISO0K16290g	PISO0L16291g
HOP2	YGL033W	DEHA0D13442g	DEHA2D12210g	PISO0K02804g	PISO0L02805g
MMS4	YBR098W	DEHA0F16434g	DEHA2F15158g	PISO0I08280g	PISO0J10107g

MSH4	YFL003C	No	No	No	No
MSH5	YDL154W	No	No	No	No
MSH2	YOL090W	DEHA0B16005g	DEHA2B15818g	PISO0D00421g	PISO0C00332g
MSH6	YDR097C	DEHA0E16566g	yes	PISO0N20495g	PISO0M20252g
SAE2	YGL175C	DEHA0C15290g	DEHA2C14212g	PISO0G14900g	PISO0H14901g
MEI5	YPL121C	DEHA0G24640g	DEHA2G23298g	PISO0I16156g	PISO0J17961g
SAE3	YHR079C-A	No	No	No	No
HOP1	YIL072W	DEHA0D16060g	DEHA2D14740g	PISO0N01245g	PISO0M01090g
RED1	YLR263W	No	No	No	No
MEK1	YOR351C	DEHA0F24684g	DEHA2F23254g	PISO0G15362g	PISO0H15363g
ZIP1	YDR285W	No	No	No	No
ZIP2	YGL249W	No	No	No	No
MND2	YIR025W	DEHA0F21802g	DEHA2F20482g	PISO0G10346g	PISO0H10347g
MAM1	YER106W	No	No	No	No
ZIP3	YLR394W	No	No	No	No
STU1	YBL034C	DEHA0F27423g	DEHA2F25872g	PISO0N06855g	PISO0M06722g
TID3	YIL144W	DEHA0E10065g	DEHA2E09548g	PISO0K11824g	PISO0L11825g
UBC11	YOR339C	No	No	No	No
RAD23	YEL037C	DEHA0B04092g	DEHA2B04180g	PISO0A07810g	PISO0B07877g
EXO1	YOR033C	DEHA0E16071g	DEHA2E15444g	PISO0N20055g	PISO0M19812g
HRR25	YPL204W	DEHA0C04884g	DEHA2C04290g	PISO0K19392g	PISO0L19393g
HUL4	YJR036C	DEHA0F12749g	DEHA2F11704g	PISO0K10592g	PISO0L10593g
LEE1	YPL054W	DEHA0D17303g	DEHA2D15928g	PISO0I00976g	PISO0E01148g
ENA2	YDR039C	DEHA0G09878g	DEHA2G09108g	PISO0C05656g	PISO0D05745g
PMC1	YGL006W	DEHA0A09295g	DEHA2A09086g	PISO0F06473g	PISO0E05152g
CMK2	YOL016C	DEHA0F10197g	DEHA2F09284g	PISO0K08172g	PISO0L08173g
CHS1	YNL192W	DEHA0D04620g	DEHA2D03916g	PISO0I04144g	PISO0J05949g
ISA1	YLL027W	DEHA0A06787g	DEHA2A06710g	PISO0C04842g	PISO0D04953g
HTZ1	YOL012C	DEHA0E06501g	DEHA2E05720g	PISO0K14992g	PISO0L14993g
ATG8	YBL078C	DEHA0D04664g	DEHA2D03960g	PISO0I04210g	PISO0J06015g
BAG7/SAC7 (WGD)	YOR134W	DEHA0G12881g	DEHA2G12122g	PISO0F10191g	PISO0E08848g
ROM2	YLR371W	DEHA0G06094g	DEHA2G05456g	PISO0C08978g	PISO0D09045g
RAS2	YNL098C	DEHA0F10351g	DEHA2F09438g	PISO0K08348g	PISO0L08349g
GNA1	YFL017C	DEHA0B05005g	DEHA2B05126g	PISO0A06974g	PISO0B07041g
SGA1	YIL099W	DEHA0A12705g	DEHA2A12254g	PISO0F03459g	PISO0J03419g
CLG1	YGL215W	DEHA0C15642g	DEHA2B03630g	PISO0A08250g	PISO0B08317g
CYB2	YML054C	DEHA0D06325g	DEHA2D05522g	PISO0A06116g	PISO0B06183g
ECM4	YKR076W	DEHA0C17798g	DEHA2C16566g	PISO0K21922g	PISO0L21923g
TOS7	YOL019W	DEHA0G09702g	DEHA2G08932g	PISO0C05832g	PISO0D05899g

ARN2	YHL047C	No	No	No	No
GTT1	YIR038C	DEHA0D17677g	DEHA2D16280g	PISO0I00624g	PISO0E00796g
RIB5	YBR256C	DEHA0D15202g	DEHA2D13926g	PISO0N01861g	PISO0M01706g
CHO1	YER026C	DEHA0B15851g	DEHA2B15686g	PISO0D00553g	PISO0C00464g
XKS1	YGR194C	DEHA0C07788g	DEHA2C06974g	PISO0I11690g	PISO0J13517g
PCT1	YGR202C	DEHA0G22858g	DEHA2G21560g	PISO0A03036g	PISO0B03169g
ELC1	YPL046C	DEHA0D14322g	DEHA2D13046g	PISO0K02144g	PISO0L02145g
SYF2	YGR129W	DEHA0C03894g	DEHA2C03366g	PISO0B10099g	PISO0A10032g
PGM2	YMR105C	DEHA0C05940g	DEHA2C05258g	PISO0K18534g	PISO0L18535g
RKI1	YOR095C	DEHA0A09537g	DEHA2A09328g	PISO0F06231g	PISO0E04910g
SUR4	YLR372W	DEHA0G06116g	DEHA2G05478g	PISO0C08956g	PISO0D09023g
PIB1	YDR313C	DEHA0E08569g	DEHA2E08030g	PISO0K13144g	PISO0L13145g
PIN3	YPR154W	DEHA0B14575g	DEHA2B14476g	PISO0D01609g	PISO0C01520g
SSO2	YMR183C	DEHA0B09790g	DEHA2B09570g	PISO0G02668g	PISO0H02669g
FBP1	YLR377C	DEHA0F01309g	DEHA2F01100g	PISO0C12652g	PISO0D12719g
GLG1/GLG2 (WGD)	YKR058W	DEHA0F18579g	DEHA2F17138g	PISO0N00959g	PISO0M00804g
ARE2	YNR019W	DEHA0F25652g	DEHA2F24222g	PISO0A11748g	PISO0B11815g
GDI1	YER136W	DEHA0F26620g	DEHA2F25102g	PISO0N06129g	PISO0M05974g
PDC1	YLR044C	DEHA0B03784g	DEHA2B03872g	PISO0A08052g	PISO0B08119g
OXR1	YPL196W	DEHA0G01661g	DEHA2G01320g	PISO0K01242g	PISO0L01243g
KGD1	YIL125W	DEHA0F19217g	DEHA2F17798g	PISO0N14709g	PISO0M14312g
DAP1	YPL170W	DEHA0F16170g	DEHA2F14872g	PISO0I08566g	PISO0J10393g
SPO7	YAL009W	DEHA0C17336g	DEHA2C16104g	PISO0K21416g	PISO0L21417g
GSG1	YDR108W	DEHA0E17578g	DEHA2E16830g	PISO0N21419g	PISO0M21176g
RMD5	YDR255C	DEHA0D14300g	DEHA2D13024g	PISO0K02166g	PISO0L02167g
EMI1	YDR512C	DEHA0C07315g	DEHA2C06512g	PISO0K17390g	PISO0L17391g
RMD8	YFR048W	DEHA0F24420g	DEHA2F22990g	PISO0N02587g	PISO0M02454g
RMD11	YHL023C	DEHA0E02695g	DEHA2E02046g	PISO0A01034g	PISO0B01189g
NEM1	YHR004C	DEHA0B03003g	DEHA2B02970g	PISO0N23113g	PISO0M22848g
SPO16	YHR153C	No	No	No	No
EMI5	YOL071W	DEHA0E14311g	DEHA2E13750g	PISO0N18537g	PISO0M18294g
MUM2	YBR057C	DEHA0G13068g	DEHA2G12298g	PISO0F10367g	PISO0E09024g
MUS81	YDR386W	DEHA0G05852g	DEHA2G05214g	PISO0C09220g	PISO0D09287g
NAM8	YHR086W	DEHA0F24706g	DEHA2F23276g	PISO0G15384g	PISO0H15385g
PFS1	YHR185C	DEHA0G22770g	DEHA2G21472g	PISO0A02948g	PISO0B03081g
ADY3/CNM67 (WGD)	YDL239C	No	No	No	No
ADY4	YLR227C	Yes	No	No	No
AMA1	YGR225W	DEHA0B14591g	DEHA2B14498g	PISO0D01587g	PISO0C01498g
CDA1/CDA2 (Gene duplication pair)	YLR307W	DEHA0C12815g	DEHA2C11836g	PISO0G12612g	PISO0H12613g
CRR1	YLR213C	No	No	No	No
DIT1	YDR403W	No	No	PISO0A12408g	PISO0B12475g

DIT2	YDR402C	No	No	PISO0A12386g	PISO0B12453g
DON1/CUE5 (WGD)	YDR273W	DEHA0E07755g	DEHA2E07260g	PISO0K13738g	PISO0L13739g
DTR1	YBR180W	DEHA0A10670g	DEHA2A10362g	PISO0I02736g	PISO0J04519g
ISC10	YER180C	No	No	No	No
MNN4	YKL201C	DEHA0B04675g	DEHA2B04796g	PISO0A07172g	PISO0B07239g
MPC54	YOR177C	No	No	No	No
MSO1	YNR049C	DEHA0F11682g	DEHA2F10692g	PISO0K09602g	PISO0L09603g
MUM3	YOR298W	DEHA0D18832g	DEHA2D17336g	PISO0A09064g	PISO0B09131g
NDT80	YHR124W	DEHA0A07392g	DEHA2A07282g	PISO0C05414g	PISO0D05503g
OSW1	YOR255W	No	No	No	No
OSW2	YLR054C	No	No	No	No
SMA1	YPL027W	No	No	No	No
SMA2	YML066C	Yes	**DEHA2F06072g**	**PISO0I14088g**	**PISO0J15893g**
SMK1	YPR054W	Yes	DEHA2E18348g	PISO0N05799g	PISO0M05644g
SPO14	YKR031C	DEHA0C03311g	DEHA2C02926g	PISO0I18224g	PISO0J19985g
SPO20	YMR017W	No	No	No	No
SPO21/YSW1 (WGD)	YOL091W	No	No	No	No
SPO71	YDR104C	DEHA0G13662g	DEHA2G12804g	PISO0F10851g	PISO0E09486g
SPO73	YER046W	DEHA0A13761g	DEHA2A13332g	PISO0A10956g	PISO0B11023g
SPO74	YGL170C	No	No	No	No
SPO75	YLL005C	DEHA0G18480g	DEHA2G17292g	PISO0G05550g	PISO0H05551g
SPO77	YLR341W	No	No	No	No
SPS1	YDR523C	DEHA0E16115g	DEHA2E15488g	PISO0N20099g	PISO0M19856g
SPS4	YOR313C	DEHA0G07007g	DEHA2G06336g	PISO0C08032g	PISO0D08099g
SSP1	YHR184W	DEHA0E08371g	DEHA2E07854g	PISO0K13342g	PISO0L13343g
SSP2	YOR242C	DEHA0C13277g	DEHA2C12320g	PISO0G13206g	PISO0H13207g
TEP1	YNL128w	DEHA0B05984g	DEHA2B06116g	PISO0N11497g	PISO0M11188g
YAL018C	YAL018c	No	DEHA2C15598g	PISO0K20954g	PISO0L20955g
YEL023C	YEL023c	No	No	PISO0K10636g	PISO0L10637g
SPO1	YNL012W	DEHA0F25861g	DEHA2F24420g	PISO0A11946g	PISO0B12013g
Spombe mug66	No	DEHA0B08591g	DEHA2B08580g	PISO0G03460g	PISO0H03461g

¥ *S. cerevisiae* and *D. hansenii* (version_1) genes were extracted from the supplementary tables 34 and 35 from Butler et al. 2009. *D hansenii* genome : * version_1 from Dujon et al. (2004), ** version_2 (open access at http://www.genolevures.org/deha.html#)The Génolevures Consortium (Souciet *et al*., 2009), In bold, orthologs in *D. hansenii* version_2; in grey, orthologs in *P. sorbitophila* absent in *D. hansenii* version_2
